# Making multi-axis Gaussian graphical models scalable to millions of cells

**DOI:** 10.1093/bioinformatics/btag523

**Published:** 2026-07-17

**Authors:** Bailey Andrew, Erica L Harris, James A Poulter, David R Westhead, Luisa Cutillo

**Affiliations:** School of Computing, University of Leeds, West Yorkshire LS2 9JT, Leeds, United Kingdom; School of Medicine, University of Leeds, West Yorkshire LS2 9JT, Leeds, United Kingdom; School of Medicine, University of Leeds, West Yorkshire LS2 9JT, Leeds, United Kingdom; School of Molecular and Cellular Biology, University of Leeds, West Yorkshire LS2 9JT, Leeds, United Kingdom; School of Mathematics, University of Leeds, West Yorkshire LS2 9JT, Leeds, United Kingdom

## Abstract

**Motivation:**

Networks underlie the generation and interpretation of many biological datasets: gene networks shed light on the regulatory structure of the genome, and cell networks can capture structure of the tumor micro-environment. However, most methods that learn such networks make the faulty “independence assumption”; to learn the gene network, they assume that no cell network exists. “Multi-axis” methods, which do not make this assumption, fail to scale beyond a few thousand cells or genes. This limits their applicability to only the smallest datasets.

**Results:**

We develop a multi-axis method, which learns conditional dependency networks, capable of processing million-cell datasets within minutes. This was previously impossible, and unlocks the use of such methods on modern scRNA-seq datasets, as well as more complex datasets. We apply the method to a new scRNA-seq dataset for neuronal cell development, and compare the result to an existing state of the art method, hdWGCNA. We demonstrate that the new method yields gene networks that have a more focused biological interpretation and that the simultaneously learned cell network has advantages over a conventional kNN-based clustering. Further, our method yields novel biological insights by identifying long non-coding RNAs that potentially have a role in neuronal development.

**Availability and implementation:**

Our methodology is available as a Python package GmGM on PyPI (https://pypi.org/project/GmGM/0.5.3/). The code for all experiments performed in this article is available on GitHub (https://github.com/BaileyAndrew/GmGM-Bioinformatics) and Zenodo (10.5281/zenodo.20384566).

## 1 Introduction

Learning a network (“graph”) that describes a dataset can improve understanding of the systems that generated it. A common example is learning a gene association network from a single-cell RNA-sequencing (scRNA-seq) dataset. These datasets typically come in the form of a cells×genes matrix, with the individual matrix entries of gene expression values; learning a gene network from this type of data gives insight into the regulatory pathways governing cell behavior. There are many methods to learn networks, such as the graphical lasso (Friedman *et al.* 2008), WGCNA and its single-celled variant hdWGCNA (Langfelder and Horvath 2008, Morabito *et al.* 2023), and neighborhood selection (Meinshausen and Bühlmann 2006).

However, these methods all make the “independence assumption”; to learn the gene network, they must assume that the cells do not interact. This is clearly false; cells come from a highly interactive micro-environment, and come in a variety of cell types—all of this information is useful for the construction of gene networks, and should not be thrown away. In this article, we propose a scalable method to learn gene networks while still capturing cellular heterogeneity; as a byproduct, our method also produces a cell network that can be used for cell type inference. By learning both networks simultaneously, one can expect to get better results than learning them sequentially—not only does our method not make the independence assumption, but it can share information between the networks when learning them, leading to better estimates of both.

Our method is a type of “multi-axis” method [which are often called “Kronecker-separable” methods (Wang *et al.* 2022)], named so because they consider the dependencies within all *axes* of a dataset; in contrast, methods that make the independence assumption are “single-axis.” In general, for any matrix-variate dataset there are two potential *kinds of dependencies* one must consider: sample (“row,” “cell”) and feature (“column,” “gene”) dependencies. This concept, and our method, can be extended to more complex (“tensor-variate”) datasets, which occasionally show up in bioinformatics (Example 3).

**Example 1**. *scRNA-seq datasets have two axes (cells, genes).*

**Example 2**. *scATAC-seq datasets have two axes (cells, peaks).*

**Example 3**. *A longitudinal bulk RNA study of multiple patients has three axes (timepoints, patients, genes).*

Also in scope are datasets with “shared axes.” A shared axis is an axis that appears in two or more matrices at a time. The goal is to learn a network describing each axis, without making an independence assumption on any.

**Example 4**. *Paired scRNA-seq/scATAC-seq datasets have three axes (genes, peaks, and a cells axis shared between the two modalities).*

**Example 5**. *A 5 patient scRNA-seq dataset has six axes (one for the cells of each patient, and a gene axis shared between them all).*

The standard independence assumption is that there are no row dependencies (i.e. there are no dependencies between cells in the scRNA-seq case), which yields efficient algorithms but is inaccurate. One could instead allow “full dependence,” i.e. every matrix element $\left(i_{1},j_{1}\right)$ could potentially be dependent on any other matrix element $\left(i_{2},j_{2}\right)$. This would allow very granular statements to be made about the data, such as “gene A in cell X affects gene B in cell Y.” Sadly, inference of such a network is statistically intractable: a d×d matrix would have $O(d^{4})$ potential dependencies, and only one “sample” (the matrix itself) from which to learn such dependencies.

To strike a balance between model accuracy and model tractability, the independence assumption is replaced with a weaker, more realistic assumption about how the axes may interact. In this article, we make the **Cartesian product assumption**, which is easiest to describe in terms of a concrete scRNA-seq example: “a gene can only be (conditionally) dependent on the expression of *other genes within the same cell*, or the *same gene in other cells*.” For other datasets, one may replace “gene” and “cell” with the relevant row/column variable. This assumption is weaker than the independence assumption, yet like the independence assumption only a more tractable $O(d^{2})$ possible dependencies need be considered.

The networks considered in this article are *conditional dependence* networks; two genes are conditionally dependent, given the rest of the genes in the dataset, if they are statistically dependent on one another after removing the effect of the other genes (Koller and Friedman 2009). Conditional dependence captures the concept of “direct correlation”; if *A* and *B* are only correlated indirectly due to their mutual correlation with another gene *C*, then they would be conditionally independent. Thus, conditional dependency networks tend to be sparser and more meaningful than co-expression networks. For Gaussian data, the inverse covariance matrix Ψ encodes the conditional dependency graph as follows:


$$\Psi_{ij}=0\Leftrightarrow \text{gene}\;i\;\text{is}\;\text{conditionally}\;\text{independent}\;\text{from}\;\text{gene}\;j$$

This convenient relationship allows our assumptions about the data to be expressed as a probabilistic model, where $\Psi^{\left(\mathrm{cells}\right)},\Psi^{\left(\mathrm{genes}\right)}$ are the cell and gene conditional dependency matrix and $d_{1},d_{2}$ are the numbers of cells and genes, respectively. In doing so, our model is amenable to maximum likelihood estimation (MLE). Letting X be our dataset, I and 0 be the identity matrix and zero matrix, vec be the operation that flattens matrices into vectors, and ⊗ be the “Kronecker product” operation, traditional single-axis models and our model can be expressed as follows (with N(μ,Σ) being the normal distribution with mean μ and covariance Σ):


$$\text{vec}\left[X\right]∼N\left(0_{d_{1}d_{2}},{\left(\Psi_{d_{2}\times d_{2}}^{\left(\mathrm{genes}\right)}⊗I_{d_{1}\times d_{1}}+I_{d_{2}\times d_{2}}⊗\Psi_{d_{1}\times d_{1}}^{\left(\mathrm{cells}\right)}\right)}^{-1}\right)\;\left(\text{Cartesian}\;\text{product}\;\left[\text{our}\;\text{method}\right]\right)$$

$$\text{vec}\left[X\right]∼N\left(0_{d_{1}d_{2}},{\left(\Psi_{d_{2}\times d_{2}}^{\left(\mathrm{genes}\right)}⊗I_{d_{1}\times d_{1}}\right)}^{-1}\right)\;\left(\text{Independence}\;\text{of}\;\text{cells}\;\left[\text{glasso},\;\text{hdWGCNA},\;\text{etc}.\right]\right)$$

The operation F⊗I+I⊗G is common enough that it is known as the “Kronecker sum” F⊕G. For two graphs F,G encoded as matrices F,G, the Kronecker sum F⊕G encodes the Cartesian product of F and G.

A Gaussian assumption would not be correct for most biological datasets, particularly those comprising of binary or count data. However, this assumption can be replaced by the much weaker “Gaussian copula” assumption, which allows the data to follow any distribution as long as the genes “interact Gaussianly”; in particular, if genes were to follow a negative binomial or Poisson distribution, they can still fit the Gaussian copula assumption. This is discussed further in Section 2.2.

This article is not the first to consider the Cartesian product assumption; there are several methods to learn such graphs. The most scalable include TeraLasso (Greenewald *et al.* 2019), which was the first to generalize to tensor-variate data, and GmGM (Andrew *et al.* 2024), which further generalized the scenario to the “shared axis” case. [Other methods that work with shared axes in multi-modal datasets do exist, but they all make the independence assumption on one of the axes; for example, the group graphical lasso and fused graphical lasso (Danaher *et al.*, 2014, Cai *et al.*, 2016), and Gaussian chain graphical models (McCarter and Kim 2014).] However, TeraLasso is only scalable to hundreds of cells, and GmGM to low thousands. Modern scRNA-seq datasets have hundreds of thousands of cells. In fact, there are 11 Human Cell Atlas datasets with more than a million cells (Regev *et al.* 2017). Extrapolating their runtimes to a million-cell dataset, TeraLasso would take 15 000 years to run and GmGM would take 200. Both would require at least 16 terabytes of memory just to store the output using single-precision floats. Even with access to a high-performance-computing (HPC) cluster, the usability of these algorithms on such datasets is dubious.

Due to these scalability constraints, multi-axis models cannot be applied to modern scRNA-seq datasets. In this article, we make significant alterations to the GmGM algorithm to improve its scalability, resulting in an algorithm that can run on million-cell datasets in a matter of minutes. Our contributions unlock the use of multi-axis models on modern datasets.

## 2 Materials and methods

### 2.1 Notation

**Figure 1:**
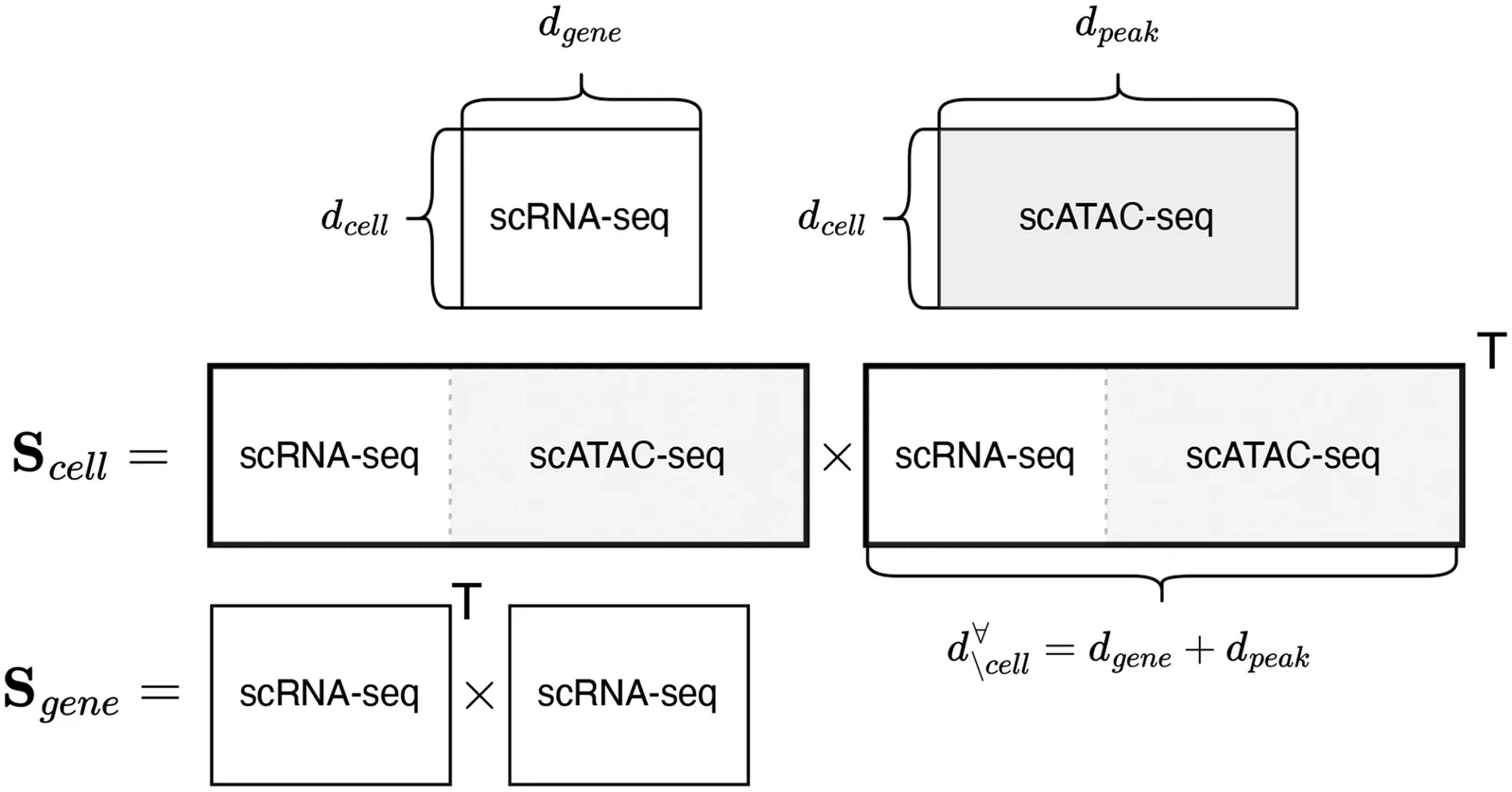
An example two-modality dataset with a cell axis shared between modalities. The top row shows data matrices and their dimensions $d_{\ell }$. The second modality is given a grey background; for single-modality data, such as scRNA-seq, it would not be present. $S_{\ell }$ denotes empirical covariance matrices. The first row of the image shows two matrices, labeled scRNA-seq and scATAC-seq, respectively. The first has its rows and columns labeled$d_{cells}$ and $d_{genes}$ , respectively. The second has its rows and columns labeled $d_{cells}$ and $d_{peaks}$, respectively. The next row contains a $d_{cells}\;\times (d_{genes}+d_{peaks})$dataset formed by concatenating the first two. Multiplying that matrix by its transpose produces $S_{cell}$. The last row shows that multiplying the the transpose of the scRNA-seq matrix by itself produces $S_{gene}$.

Because our model is built to work on a wide variety of datasets (including those with shared axes and more than two axes), the notation can be quite dense; in particular, many variables of the form $d_{y}^{x}$ are defined below. For most users, only the matrix-variate case (which includes scRNA-seq) will be of interest, for which the notification can be drastically simplified. For those interested in how the definitions of various values change when working with tensor-variate data, we refer the reader to Appendix A, available as supplementary data at *Bioinformatics* online. In Fig. 1, we give a graphical summary of some of the notation introduced in this section.

#### 2.1.1 Matrix-variate data

There are only two axes in matrix-variate data, with sizes $d_{1}$ and $d_{2}$. From the perspective of learning the $d_{1}\times d_{1}$ row graph, we have $d_{\backslash 1}=d_{2}$ samples, and, from the perspective of learning the $d_{2}\times d_{2}$ column graph, we have $d_{\backslash 2}=d_{1}$ samples. There are $d_{\forall }=d_{1}d_{2}$ matrix entries in total.

We will refer to generic datasets as X. Subscripts typically represent a choice of axis, often denoted ℓ. For example, $\Psi_{\ell }$ will represent the precision matrix for axis ℓ.

${\text{det}}^{†}$ represents the *pseudo-determinant* and vec represents vectorization. The $d_{\ell }\times d_{\ell }$ empirical covariance matrix $S_{\ell }$ is either $XX^{T}$ or $X^{T}X$ depending on whether ℓ represents the rows or columns of the dataset. $S_{\ell }$ has a rank-$r_{\ell }$ eigendecomposition with eigenvectors $V_{\ell }^{\left(r_{\ell }\right)}$ and eigenvalues $E_{\ell }^{\left(r_{\ell }\right)}$.

#### 2.1.2 Multi-modal matrix data

When the dataset is multi-modal, we will use a superscript γ to denote which modality in the dataset we are referring to. For example, if we have a 1000-cell, 2000-gene scRNA-seq dataset paired with a 1000-cell, 100 000-peak scATAC-seq dataset, we have $d_{\text{cell}}=1000$, $d_{\text{gene}}=2000$, and $d_{\text{peak}}=100\;000$, with $d_{\backslash \text{cell}}^{\text{scRNA}}=2000$, $d_{\backslash \text{cell}}^{\text{scATAC}}=10\;000$, and $d_{\forall }^{\text{scRNA}}=2\;000\;000$. In this case, $d_{\backslash \ell }^{\forall }=\sum_{\gamma }^{\Gamma }d_{\backslash \ell }^{\gamma }$ represents the total number of samples in the whole dataset, from the perspective of axis ℓ. We will use the shorthand ℓ∈γ to represent all axes that appear in a modality γ—e.g. cell∈scRNA but peak∉scRNA as that axis only appears in the scATAC modality.

The definition of $S_{\ell }$ also changes slightly, as shown in Fig. S1, available as supplementary data at *Bioinformatics* online. In this case, $S_{\text{cell}}=\left[\begin{array}{cc} X^{\text{scRNA}} & X^{\text{scATAC}} \end{array}\right]{\left[\begin{array}{cc} X^{\text{scRNA}} & X^{\text{scATAC}} \end{array}\right]}^{T}$.

### 2.2 Assumptions

The five assumptions of this work are listed below, citing where they have been used for multi-axis modeling before.

Assumption 1. *The data’s dependencies are Cartesian-product-structured. This means that a matrix element (such as a cell-gene pair)* $\left(c_{1},g_{1}\right)$  *may be conditionally dependent on* $\left(c_{1},g_{2}\right)$  *and* $\left(c_{2},g_{1}\right)$  *but not* $\left(c_{2},g_{2}\right)$*. First used by Kalaitzis et al. (2013).*

Assumption 2. *The data has a Gaussian copula, also called the “nonparanormality assumption.” Used by Li et al. (2022). This assumption allows the data to have any marginal distribution (such as negative binomial), as long as the genes interact “Gaussian-ly.”*

Assumption 3. *Conditional dependency graphs with d vertices have* O(d)  *edges ,i.e. are “linearly sparse.” Follows from Assumption A4 of Greenewald et al. (2019).*

Assumption 4. *The largest nonzero eigenvalue of* $\Psi_{\ell }$  *is at least* $\epsilon_{\ell }$*, for some small* $\epsilon_{\ell }$*. Follows from Assumption A2 of Greenewald et al. (2019).*

Assumption 5 (New). *The input data and true graphs are well-approximated by low-rank matrices. This assumption is common throughout data science (see Appendix C, available as supplementary data at Bioinformatics online) but has not been applied to improve performance for multi-axis models.*

Our method inherits the nonparanormality assumption from prior work. Intuitively, this corresponds to a multivariate distribution with arbitrary marginals but whose variables interact with each other “like Gaussian variables.” This is done by only considering rank statistics of gene expression, rather than the exact expression values. See Liu *et al.* (2012) for more details. To our knowledge, there has been no Kronecker-structured work that does not make this or a stronger assumption. If there were, it would likely lack the crucial properties of the Gaussian copula that make it efficient to scale (namely the relationship between Ψ and the graph of conditional dependencies)—although it is certainly a worthwhile avenue for future work to explore.

The main bottleneck to scalability in the prior version of GmGM was that its memory complexity was $O(\sum_{\ell }d_{\ell }^{2})$. Without making any additional assumptions, this memory complexity is optimal; a set of fully dense $d_{\ell }\times d_{\ell }$ precision matrices require exactly $\sum_{\ell }\frac{d_{\ell }^{2}+d_{\ell }}{2}$ values to specify. However, when working with conditional dependencies, the output graphs will typically be sparse. The “linearly sparse” assumption makes it at least theoretically possible to achieve $O(\sum d_{\ell })$ memory complexity. Real world networks are often linearly sparse. Consider the case of a social network; no matter how many people *p* there are in the world, there is (sadly) a constant upper bound on the possible number of friends someone can make during their life. This yields a graph with O(p) edges.

While linear sparsity makes a linear memory cost possible, all prior work relies on eigendecompositions. This entails the use of the matrix of eigenvectors, one for each axis, requiring $\sum_{\ell }d_{\ell }^{2}$ elements. To achieve scalable memory usage, this must be avoided. Thankfully, if the dataset is well-approximated by a low-rank matrix, then only the first few eigenvectors are actually meaningful! The full eigendecomposition is not needed. This assumption is easily verifiable on real data; PCA is a standard part of many data analysis pipelines, with there being many techniques to estimate the number of principal components (i.e. eigenvectors) required to capture most of the variance in the data.

### 2.3 The model

Our model is similar in structure to TeraLasso and the previous version of GmGM in that they both are based on a Gaussian distribution with a Kronecker-sum-structured precision matrix; however, to take advantage of Assumption 5 a “*singular*” Gaussian probability distribution is used instead; see Appendix E, available as supplementary data at *Bioinformatics* online for an explanation and derivation. Letting $r_{\ell }$ be the rank chosen for each axis, and $r_{\forall }^{\gamma }$ be the product of ranks of all axes occurring in modality γ, our model can be expressed as follows:


(1) $${\text{pdf}}_{\text{normal}}(X^{\gamma })=\frac{\sqrt{{\text{det}}^{†}\left(\underset{\ell \in \gamma }{⊕}\Psi_{\ell }\right)}}{{\left(2\pi \right)}^{\frac{r_{\forall }^{\gamma }}{2}}}e^{-\frac{1}{2}\sum_{\ell \in \gamma }\text{tr}\left[S_{\ell }^{\gamma }\Psi_{\ell }\right]}$$

When given a multi-modal dataset, the model is:


$${\text{pdf}}_{\text{normal}}\left(\left\{X^{\gamma }\right\}\right)=\prod_{\gamma }\frac{\sqrt{{\text{det}}^{†}\left(\underset{\ell \in \gamma }{⊕}\Psi_{\ell }\right)}}{{\left(2\pi \right)}^{\frac{r_{\forall }^{\gamma }}{2}}}e^{-\frac{1}{2}\sum_{\ell \in \gamma }\text{tr}\left[S_{\ell }^{\gamma }\Psi_{\ell }\right]}$$

Above, the data are assumed to be Gaussian (x∼N(·,·)). To achieve the weaker “Gaussian copula” assumption (Assumption 2), which states that the dataset is a *monotonic transformation f* of a Gaussian dataset ($f^{-1}(x)∼N(\cdot ,\cdot )$), we follow Li *et al.* (2022) in using the Nonparanormal Skeptic (Liu *et al.* 2012) to first compute the sufficient statistics $\left\{S_{\ell }\right\}$, before applying our model. The pseudocode for the algorithm we develop to fit this model is given in Appendix B, available as supplementary data at *Bioinformatics* online.

### 2.4 Theory

The aim of this article is to find the maximum likelihood estimator (MLE) of Equation 1 efficiently. A major bottleneck in prior work was the need to re-compute eigenvectors every iteration. Appendix I, available as supplementary data at *Bioinformatics* online contains the proof for the following theorem:

Theorem 1. *Let the rank-* $r_{\ell }$ *partial eigendecomposition of* $S_{\ell }$  *be* $V_{\ell }^{\left(r_{\ell }\right)}E_{\ell }^{\left(r_{\ell }\right)}V_{\ell }^{(r_{\ell }),T}$*. Then* $V_{\ell }^{\left(r_{\ell }\right)}$  *are also eigenvectors of the maximum likelihood estimate* ${\hat{\Psi }}_{\ell }$.

This reduces the problem to finding the eigenvalues, which lack an exact solution but can be solved for iteratively due to the convexity of the problem. The next theorem shows that this is a well-founded problem:

Theorem 2. *There exists a unique MLE that estimates the precision matrix of the singular Kronecker-sum-structured normal distribution, provided* $r_{\ell }\leq \text{rank}\left[S_{\ell }\right]$.

*Note that* $r_{\ell }\leq d_{\backslash \ell }^{\forall }$  *suffices. For matrix-variate data, it suffices to choose ranks* $\leq \text{min}\left(d_{\text{rows}},d_{\text{cols}}\right)$.

Kronecker-structured models often suffer identifiability issues; in Appendix F, available as supplementary data at *Bioinformatics* online it is shown that there exists an identifiable parameterization. In Appendix K, available as supplementary data at *Bioinformatics* online, a hypothesis testing procedure is derived to allow one to deduce the statistically significant edges.

Theorem 3. *Under the null hypothesis* $\underset{\ell \in \gamma }{⊕}\Psi_{\ell }=\frac{1}{{\left(\sigma^{\gamma }\right)}^{2}}I_{d_{\forall }^{\gamma }}$*, the following distribution holds for each* $\psi_{\ell_{ij}}$  *independently:*
 

$$\sqrt{\frac{\sum_{\gamma |\ell \in \gamma }d_{\forall }^{\gamma }{\left(\sigma^{\gamma }\right)}^{4}}{d_{\ell }}}\frac{\psi_{\ell_{ij}}}{2}\;\dot{∼}\;N\left(0,1\right)$$

In practice, we have found this test to be quite weak: unless the dataset is very large, or one axis is much larger than the other, there are few statistically significant edges found. Thresholding may be preferred, especially if the graph is used as a preprocessing step (such as for clustering) rather than an end in and of itself. For more information on choosing thresholding parameters, we refer the reader to Appendix D, available as supplementary data at *Bioinformatics* online.

## 3 Results

The scalability of our method is demonstrated in Section 3.1. In Section 3.2, we present an exploration of the role of long non-coding RNA (lncRNA) in neural progenitor cells. In biology, the ground truth is often not known; in Appendix N, available as supplementary data at *Bioinformatics* online, there is an additional comparison on a non-biological real-world toy dataset with known ground truth to validate our methodology.

### 3.1 Scalability

**Figure 2:**
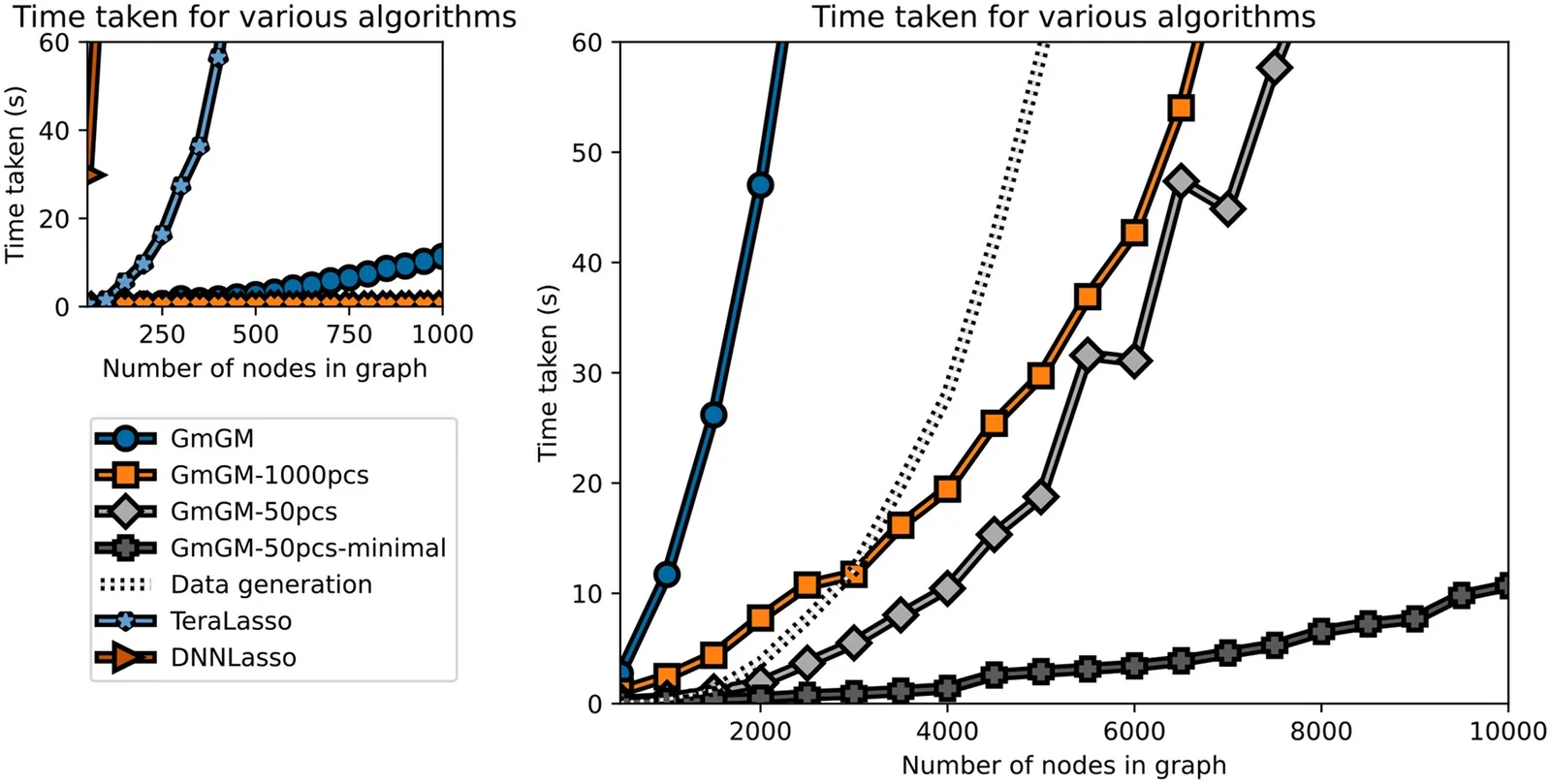
The running time of various multi-axis algorithms on synthetic data as the number of nodes in the graph increases. The synthetic datasets were generated from a Kronecker-sum-structured normal distribution The top-left focuses in on the graphs with <2000 nodes to be able to show TeraLasso (Greenewald *et al*. 2019) and DNNLasso (Lin and Zhang 2024). “GmGM” refers to the old GmGM (Andrew *et al*. 2024), whereas GmGM-Xpcs refers to our modifications, using only *X* principal components. GmGM-50pcs-minimal corresponds to the case in which the number of edges in the graph is proportional to the number of nodes (Assumption 3); for the other models, the full graph was kept.

In this section, the scalability of our method is demonstrated, comparing it to two other multi-axis methods on in-distribution synthetic data. (See Appendix N, available as supplementary data at *Bioinformatics* online for precision/recall comparisons on said data.) The synthetic datasets were generated from a Kronecker-sum-structured normal distribution, and the ground truth networks to be learned were generated from a Barabasi-Albert distribution (Barabási and Albert 1999). The Barabasi-Albert distribution was chosen as it is a power law distribution; many real world networks approximately follow a power law. Figure 2 shows how much more scalable our modification makes GmGM, with it being the only method that can feasibly run on datasets of several thousand cells. Table 1 presents estimated memory usage of our algorithm and prior work.

**Table 1:** The raw memory size requirement of various input datasets when running our methodology without the Nonparanormal Skeptic.^a^

Shape	Average vertex degree	Rank	Double-precision floats	Bytes	Naive bytes
(50, 50)	5	50	2×50×50 + 4×50 + 2×5×50 = 5700	45.6 kB	161 kB
			Eigenvectors Eigenvalue Output graphs		
(50, 50)	5	10	2×10×50 + 4×10 + 2×5×50 = 1540	12.3 kB	161 kB
			Eigenvectors Eigenvalue Output graphs		
(50, 50)	20	50	2×50×50 + 4×50 + 2×20×50=7200	57.6 kB	161 kB
			Eigenvectors Eigenvalue Output graphs		
(1000, 50)	5	50	57 950	463 kB	32 MB
(1000, 1000)	5	50	110 200	882 kB	64 MB
(1 248 980, 2200)	5	2200	2.76 billion	22 GB	50 TB
(1 248 980, 2200)	5	50	69 million	551 MB	50 TB

a The Nonparanormal Skeptic will require an additional memory overhead equivalent to the size of the dataset in memory. Some subroutines, such as eigendecomposition, may require a small constant multiple (2 or 3) of additional memory on top of this, but this is implementation-dependent. The “Naive bytes” column shows the memory usage if one used previous algorithms, which stored $S_{\ell },V_{\ell },E_{\ell },\;\text{and}\;\Psi_{\ell }$ without taking advantage of sparsity or low-rank approximations. For this, the vertex degree and rank columns are ignored.

To emphasize this point, our method was run on a 1 248 980 cell PBMC dataset by Yazar *et al.* (2022), keeping the top 2200 highly variable genes, top 50 principal components (∼50% of the variance) and only the statistically significant edges (5890 of them). Our method ran in two minutes on a personal computer (see Section N, available as supplementary data at *Bioinformatics* online for more details and a sanity-checking of the results). It should be noted that prior multi-axis work is fundamentally not scalable to problems of this size, *even with access to high performance computing clusters*.

On a theoretical level, the scalability of our method is achievable in large part due to its lower computational complexity. For a dataset of O(d) genes and cells, prior work required an $O(d^{3})$ runtime and $O(d^{2})$ space use; in contrast, ours requires $O(d^{2})$ time and O(d) space. A derivation of the computational complexity, as well as its full statement for tensor-variate and shared-axis datasets, is given in Appendix G, available as supplementary data at *Bioinformatics* online.

### 3.2 Application to single cell neuronal differentiation data

The previous section proved the scalability of our method; in this section, we will demonstrate that our method leads to real biological insights, comparing to the single-axis hdWGCNA (Morabito *et al.* 2023) methodology, which identifies gene co-expression networks. As hdWGCNA is single-axis, it cannot be used to identify cell networks. Method comparison for biological networks is difficult, as there is no ground truth; however, we will see that GmGM leads to networks with different structure than hdWGCNA, and that these differences result in new biological insights. In particular, GmGM yields gene modules that are more specific to the relevant biology in our dataset. Both methods are applied to a scRNA-seq dataset of induced pluripotent stem cells (iPSCs), measured at 4 different timepoints as they develop into neural progenitor cells; the dataset consists of 6876 cells and 29 324 genes. See Appendix O, available as supplementary data at *Bioinformatics* online for details on how the data was collected.

To generate the GmGM network, the top 100 principal components were kept and the Nonparanormal Skeptic was used. The top 5 strongest edges per gene/cell were retained. To generate gene clusters (“modules”) from the learned gene network, the Leiden (Traag *et al.* 2019) algorithm was used with a “resolution” parameter of 3 (chosen to give at least 100 modules). For the hdWGCNA network, “soft power” of 4 was determined to be optimal by the hdWGCNA package’s TestSoftPower’s method. We followed hdWGCNA’s guidelines to ensure that the network and clustering obtained were the best possible, as defined by the authors of hdWGCNA. The hdWGCNA package’s recommended clustering was used for the modules (only 8 modules); a finer clustering was attempted with Leiden, although this resulted in many singletons and very small clusters. GmGM learns conditional dependency and hdWGCNA learns co-expression; these types of networks have fundamentally different topologies which is the likely cause of this difference. We did not remove lowly variable genes, as we were interested in analyzing the entire gene network.

The networks were evaluated by analyzing clusterings on them. We identified 119 gene modules on the GmGM network, labeled m0, m1, m2, and so on. A short description of each is given in Appendix S, available as supplementary data at *Bioinformatics* online (Table 10, available as supplementary data at *Bioinformatics* online). The modules range from having 37 genes (m118) to 600 genes (m2), with the exception of m1 with 1756 genes and m0 with 9047. hdWGCNA identified 7 gene modules (ranging from 241 to 6399 genes each)—an eighth “unnassigned” module contained the 14 405 genes that were not assigned to any module by hdWGCNA. A short description of each is given in Appendix S, available as supplementary data at *Bioinformatics* online (Table 9, available as supplementary data at *Bioinformatics* online). To limit the potentially confounding effects of cluster quantity on our analysis, an additional clustering was produced for each method. These were an 8 module clustering on the GmGM network (“Coarse GmGM”) and a 119 module clustering on the hdWGCNA network (“Fine hdWGCNA”).

The networks and clusterings considered had very different properties. The hdWGCNA network contained many singletons (the genes in the unassigned module) whereas GmGM contained none. The GmGM network also had a higher transitivity (the tendency for connected genes to have neighbors in common) than hdWGCNA (0.055 versus 0.002). For a network with the same total edge count as GmGM and hdWGCNA, but with i.i.d. random edges (i.e. “Erdos-Renyi”), the transitivity will on average be equal to the network density (0.0003). The GmGM, coarse GmGM, fine hdWGCNA, and hdWGCNA clusterings achieved modularities (the tendency for a clustering to have more within-cluster than between-cluster edges) of 0.569, 0.600, 0.347, and 0.220, respectively. On ten independent Erdos-Renyi random graphs based on the GmGM network, using Leiden clustering with the same parameters used for the GmGM results, we found all modularities between 0.270 and 0.272; when based on the coarse GmGM results, the modularities were all identically 0. For hdWGCNA, these values were between 0.469 and 0.475, and for fine hdWGCNA they were between 0.442 and 0.450.

To compare methods in terms of their ability to extract biologically relevant information, we evaluated how well their clusterings aligned with a set of curated GO terms. The GO terms were picked to either represent a fundamental biological process (such as the cell cycle or DNA replication) or the development of a key component of neurons (axons, dendrites, synapses, the myelin sheath, and more broadly nervous system development in general). Appendix Q, available as supplementary data at *Bioinformatics* online (Table 7, available as supplementary data at *Bioinformatics* online) contains the GO terms chosen as well as the rationale behind the choice.

**Figure 3:**
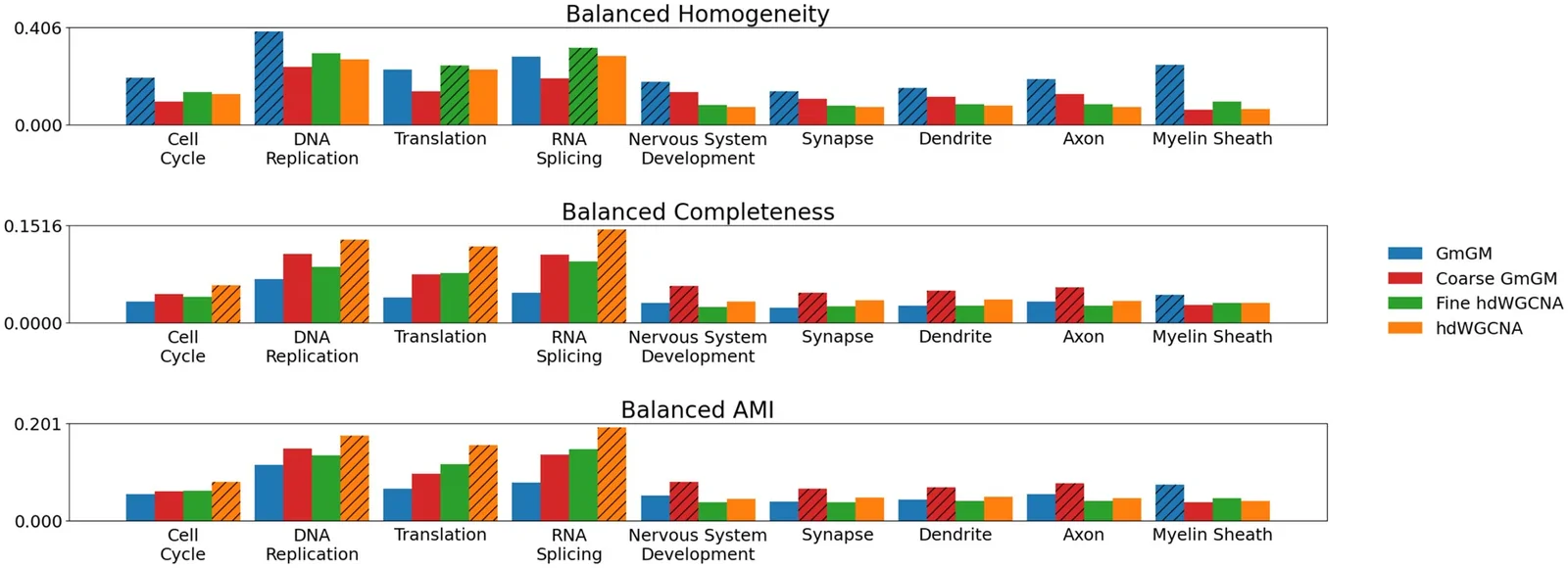
A comparison of the homogeneity and completeness of clusterings our method (GmGM) and hdWGCNA across various metrics over different sets of GO terms (see Appendix Q, available as supplementary data at *Bioinformatics* online for GO ids). Homogeneity measures the tendency for modules to contain only genes of a given set of GO terms, whereas completeness measures the tendency for a set of GO terms to all be contained in a single module. AMI stands for adjusted mutual information. “Coarse GmGM” refers to a clustering on GmGM with 7 modules (the same as hdWGCNA), and “fine hdWGCNA” refers to a clustering on hdWGCNA with 119 modules (the same as GmGM), most of which were singletons. These metrics were adjusted for chance following Romano *et al*. (2016).

The GO term comparison is given in Fig. 3. It shows that in all 5 neuron-specific GO terms considered, our method achieved higher homogeneity, completeness, and AMI, while hdWGCNA performed better across all 4 non-specific GO terms, except for homogeneity for the cell cycle and DNA replication GO terms. This is unsurprising; when we qualitatively inspect hdWGCNA modules (Appendix S’s Table 7, available as supplementary data at *Bioinformatics* online), only two of the eight modules are associated with GO terms specific to neurons (the “blue” and “red” clusters); GmGM, in contrast, had many (Appendix S’s Table 10, available as supplementary data at *Bioinformatics* online).

In general, a qualitative analysis shows that every module of hdWGCNA (except the “unassigned” module) clearly relates to some biological process, although typically a broad process such as RNA binding. In contrast, only 39 of GmGM’s 119 modules show something of immediate biological interest. However, those that do tend to be more specific than hdWGCNA; GmGM can capture biological processes that are more relevant to the domain at hand (in this case, neural development). For a module-by-module breakdown, see the tables of Appendix S, available as supplementary data at *Bioinformatics* online.

#### 3.2.1 The cell network

Another benefit of GmGM is its ability to simultaneously learn a cell network; this is not available to hdWGCNA, which only learns the gene graph. This cell network was clustered (Leiden, resolution = 1) and each cluster was associated with a cell type based on its statistical association with a set of marker genes (Appendix P, Table 6, available as supplementary data at *Bioinformatics* online). The resulting clustering is shown in Fig. 7, available as supplementary data at *Bioinformatics* online.

All cell clusters were statistically significantly associated with gene modules (Table 8, available as supplementary data at *Bioinformatics* online); e.g. the cortical neuron cells significantly express the modules m3, m13, and m27. The relation of these clusters to cortical neurons is confirmed by their associated GO terms (including “forebrain development” [GO:0030900, *P* = 2.3 × 10^−4^], “synaptic signalling” [GO:0099536, *P* = 3 × 10^−23^], and “dendrite” [GO:0030425, *P* = 1.6 × 10^−18^]—see Table 10, available as supplementary data at *Bioinformatics* online) and their dominant genes [the m27 sub-network is dominated by *TBR1*, which is a key transcription factor for early cortical neurogenesis (den Hoed *et al.* 2018, Crespo *et al.* 2022)].

While traditional methods like hdWGCNA cannot be used to learn the cell and gene networks simultaneously, a typical workflow to produce cell clusters is to use Leiden clustering on the k-nearest-neighbors (kNN) graph. Picking a Leiden resolution on the kNN graph to produce the same amount of clusters as GmGM (resolution = 2), we followed the same workflow as GmGM to label each cell with a cell type (Fig. 7, available as supplementary data at *Bioinformatics* online). The networks broadly agreed, with some notable differences. Firstly, kNN was less able to distinguish astrocytes from ectoderms (6% of cells being labeled “astro/ecto”) than GmGM (3% of cells).

Secondly, GmGM identified cells at the second and third timepoints that were not NPCs (“early differentiators”), whereas kNN identified all such cells as NPCs. We found that non-early-differentiator NPCs were statistically significantly associated with several NPC marker genes (*NES*, *PAX6*, *VIM*), whereas early differentiators only associated with *VIM*. Furthermore, they were statistically significantly associated with the *MAP2* cortical neuron marker. This supports the theory that they represent cells that have begun differentiating before the fourth timepoint. This group is heterogeneous; there are 267 such cells, 196 of which GmGM labeled as neural ectoderm and 44 of which GmGM labeled as cortical neurons. There is also a group of NPC cells (labeled by GmGM) in the fourth timepoint that are not labeled as such by kNN, although we were unable to conclusively show that these represented “late differentiators.”

It is not surprising that kNN missed the early differentiators; kNN is based on expression-distance relationships between cells, which is why the clusters look so spatially coherent in the UMAP plot (Fig. 7, available as supplementary data at *Bioinformatics* online), as UMAP is also based on expression-distance. The timepoint that a cell comes from seems to exert a strong signal in expression-distance relationships, such that the UMAP plot can be cleanly divided into four regions representing the timepoints (Fig. 6, available as supplementary data at *Bioinformatics* online). kNN is more susceptible to this signal, and hence it is more inclined to associate cells in the same timepoints together. GmGM is not based on expression-distance, and thus avoids this.

#### 3.2.2 Qualitative analysis of the gene network

We conclude this section with a brief exploration of selected modules of the GmGM network. The m90 module was especially interesting, as it had GO terms indicating a role in neurogenesis (GO:0022008, *P* = 1.5 × 10^−4^) and transcription factors (GO:0003700, *P* = 1.6 × 10^−3^). The most central genes for this module were *NFIA* and *NFIB—*known to be crucial for nervous system development (Deneen *et al.* 2006, Piper *et al.* 2010)—and the unnamed lncRNA *ENSG00000243620*. *ENSG00000243620* and another unnamed lncRNA (*ENSG00000286757*) were found in the GmGM network to be strongly connected to the *ZIC1*, *ZIC2*, and *ZIC4* transcription factors, also known to be important in neural development (Aruga 2004). They lie on chromosomes 3 and 13, respectively; *ENSG00000243620* is only 50 kilobases from *ZIC1* and *ZIC4*, whereas *ENSG00000286757* is only 20 kilobases from *ZIC2* (and *ZIC5*, not in this module). The closeness on the genome may indicate a regulatory relationship between *ZIC* genes and the unnamed lncRNAs.

By their nature of being unnamed lncRNA, *ENSG00000243620* and *ENSG00000286757* have been sparsely studied, with only 4 and 1 publications referencing those gene IDs appearing on Google Scholar, respectively. However, a study by Miao *et al.* (2026) showed a relation between these lncRNAs and ZIC genes in neural stem cells within the human fetal spinal cord.

The two other neurogenesis-and-transcription-factor-associated modules were also investigated (GO:0003700 with *P* = 3.6 × 10^−3^ and *P* = 3.2 × 10^−2^, respectively for m13 and m8) (m13 and m8). m13 associated with the genes *EBF2*, *EBF3*, and *NEUROD4*, all of which are relevant to neural development (Garcia-Dominguez *et al.* 2003). The strongest connections in this module often involve the *ELAVL1*, *ELAVL3*, and *ELAVL4* genes, one of which is between *ELAVL2* and an unnamed lncRNA “*ENSG00000283982*” 17 kilobases away. Finally, m8 is not particularly dominated by any one set of genes, but one of the strongest connections in the module is between *SHISA9* and an unnamed lncRNA “*ENSG00000262801*” 5000 base pairs away. We could not find publications mentioning these lncRNAs in any capacity.

The hdWGCNA network was checked to see if it also identified relationships between the same lncRNAs. The lncRNA results show that the m8 and m13 lncRNAs of interest are unimportant in the hdWGCNA network: each only connected to 5 other genes, on other chromosomes. In contrast, the m90 lncRNAs of interest are both highly central to hdWGCNA’s “brown” module, being in the top 4 most central (behind *DMD* and *MIR100HG*), and each having >100 connections to other genes, including the *ZIC* genes.

## 4 Discussion

In this article, we have developed a novel and general multi-axis methodology able to produce results for large-scale scRNA-seq datasets, and proven this by applying it to a million-cell dataset. Furthermore, we show that our methodology is able to extract novel biological insights by finding promising connections between transcription factors and some poorly studied lncRNA genes. We maintain the applicability of GmGM to tensor-variate datasets, which are uncommon in bioinformatics, and multi-modal datasets. For example, GmGM is also applicable to paired scRNA+scATAC datasets, among others, and has been used on them before (Andrew *et al.* 2024).

We compared our results to hdWGCNA, demonstrating that our method is able to create networks conducive to finer clustering, albeit with a higher proportion of clusters for which no immediate biological meaning is apparent. The results from hdWGCNA lend credence to a potential ZIC-lncRNA relationship found by our method and mentioned in a study by Miao *et al.* (2026), which warrants further study. By simultaneously learning both the gene and cell graph, one gains multiple means of learning new biological insights and validating results. This is a benefit our methodology has over more traditional single-axis methodologies like hdWGCNA, which only learn gene graphs.

We chose to compare against hdWGCNA and kNN as they represent the methods used *in practice* for learning gene and cell networks, respectively. Notably, neither method learns conditional dependency networks; it is reasonable to ask to what extent the differences in our networks are down to the multi-axis nature of GmGM rather than the conditional dependency nature. This is less important from a practical perspective, in which end users of our method will primarily be concerned with *whether* it works than *why* it works. Nevertheless, the *why* can be useful in directing future methodological research. In Appendix R, available as supplementary data at *Bioinformatics* online, we perform a basic comparison of GmGM against the analogous single-axis baseline, to isolate the effect of the multi-axis methodology. We believe that conditional dependency networks are more informative than correlation networks; however, there is nothing stopping future work from applying the same Cartesian product approach to a correlation network instead.

### 4.1 Limitations

Our method has several limitations. To achieve scalability, GmGM does not use an L1 penalty, relying only on thresholding to yield a sparse graph. The development of a scalable algorithm using such a penalty would be beneficial. GmGM also makes a Gaussian (or more generally a nonparanormal) assumption, which aids scalability but is simplistic. In particular, nonparanormality does not account for “tail dependence” (the tendency for variables to be correlated in the extremes). It is possible to find genes which exhibit tail dependence, at least empirically. In concurrent work, we are investigating models that do not make such an assumption, but they are again significantly less scalable.

In this article, we proposed a method for keeping only the statistically significant edges. However, we found that the test we used was very weak, in the sense that, except for very large datasets, often no edges were found to be significant. It would be interesting for future work to develop more powerful tests; in this work, we relied on thresholding by keeping the top *k* edges per vertex, analogous to the construction of kNN networks, as we found that these networks had better properties than those found using global thresholding. This is suitable when one wants to cluster on the learned networks, as it prevents singletons, but will inflate false positives when one is interested in the edges of the network themselves.

## Supplementary Material

btag523_Supplementary_Data

## Data Availability

The neuronal differentiation scRNA-seq dataset has been made available on Zenodo: https://doi.org/10.5281/zenodo.21671352.
