# Supplementary material for: Making multi-axis Gaussian graphical models scalable to millions of cells
Source: Bioinformatics. 2026 Jul 17;42(8):btag523. doi: 10.1093/bioinformatics/btag523 (PMC13440671; doi:10.1093/bioinformatics/btag523)
Supplement: Supplementary file 1 [file btag523_supplementary_data.pdf]

Contents

A Tensor-variate notation 11
B Algorithm 12
C Evidence for the low-rank approximability assumption 13
D Choice of Hyperparameters 14
E Derivation of the probability distribution 14
F Identifiability 15
G Computational complexity 18
H Kronecker product and stridewise-blockwise trace lemmas 18
I Finding the maximum likelihood estimator 19
J Existence and uniqueness 21
K Derivation of the Fisher information 23
L Preserving input sparsity 28
M Implementation 28
N Additional experiments 29
N.1 Synthetic data 29
N.2 COIL Dataset 30
N.3 Sanity check of million-cell results 31
O Neuronal scRNA-seq dataset details 32
O.1 Culture of induced pluripotent stem cells (iPSCs) 33
O.2 iPSC-derived neural progenitor cell (NPC) differentiation 33
O.3 Cell fixation 33
O.4 Barcoding, cDNA library preparation and sequencing 33
P Neuronal dataset cell marker genes 33
Q GO terms for network metrics 33
R Neuronal scRNA-seq: comparison against a simple baseline 33
S Neuronal scRNA-seq dataset full results 34

A Tensor-variate notation

In the main paper, we focused on matrix-variate notation as that is most relevant to the majority of readers. However, our model works more generally for multi-modal tensor-variate datasets, and so here we give the notation needed to prove our results in their full generality. Figure S1 gives a graphical overview of the most complicated bit of the notation.

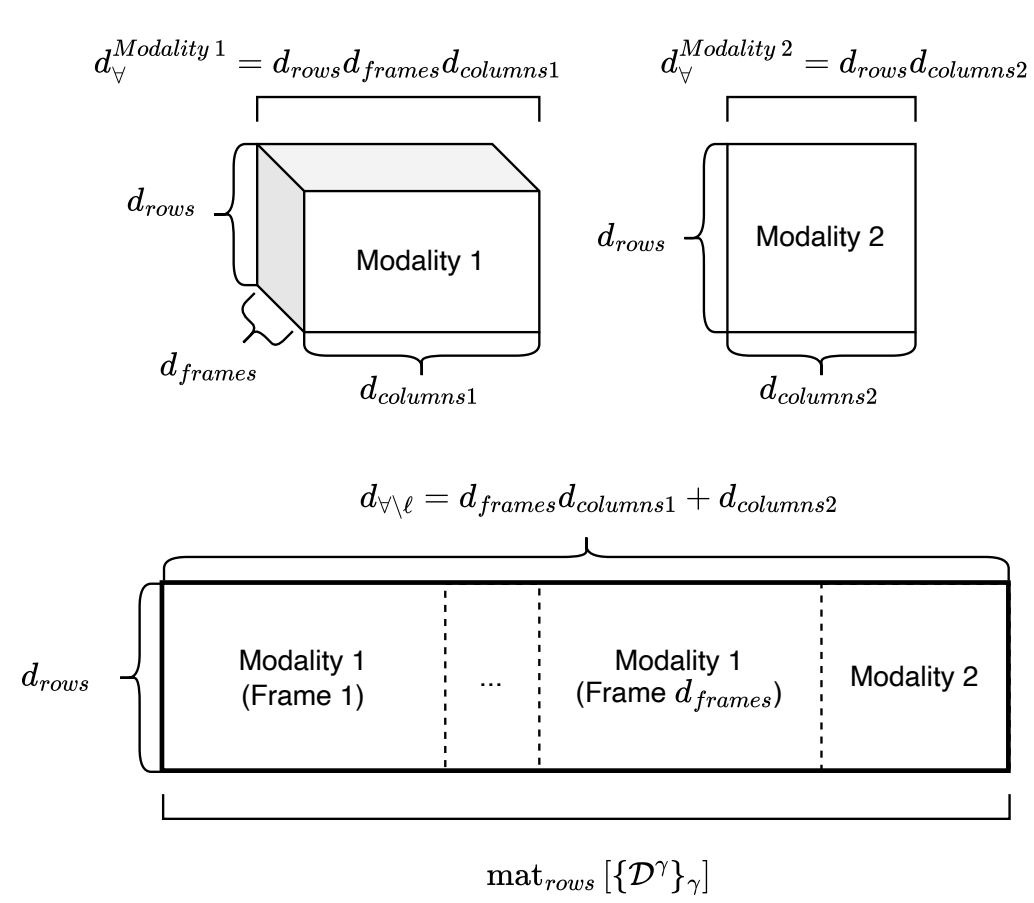

Fig. S1. An example two-modality dataset with a few relevant properties highlighted. Also contains a graphical definition of $\mathrm{mat}_\ell\left[\{\mathcal{D}^\gamma\}_\gamma\right]$ (bottom).

Axes are denoted using subscripts (often with the symbol ℓ); for example, $\mathbf{\Psi}_{\text{cells}}$ would be the precision matrix of the cells axis. Modalities will be represented with superscripts (often with the symbol γ); in a multi-patient scRNA-seq study, $\mathbf{D}^{\text{patient1}}$ would represent the cells $\times$ genes matrix of the first patient. $\ell \in \gamma$ represents the statement that an axis is recorded in a modality; in a paired scRNA/ATAC-seq dataset, "genes $\in$ scRNA-seq modality" but "genes $\notin$ scATAC-seq modality".

Let d_ℓ be the size of axis ℓ, $d_\forall^\gamma = \prod_{\ell\in\gamma} d_\ell$ be the size of modality γ, $d_{\backslash\ell}^\gamma = \frac{d_\forall^\gamma}{d_\ell}$ be the number of samples from ℓ's perspective in γ, and $d_{\backslash\ell}^\forall = \sum_{\gamma|\ell\in\gamma} d_{\backslash\ell}^\gamma$ be the number of samples from ℓ's perspective overall. r_ℓ and analogous variables are defined to represent the number of principal components chosen for axis ℓ.

$\det^\dagger$ represents the *pseudo-determinant* and vec represents vectorization. $\text{mat}_\ell\left[\mathcal{X}^\gamma\right]$ represents the 'matricization' of a tensor $\mathcal{X}^\gamma$, which reshapes the data into a $d_\ell \times d^\gamma_{\backslash\ell}$ matrix. Matricization of a dataset, $\text{mat}_\ell\left[\{\mathcal{X}^\gamma\}_\gamma\right]$, reshapes the data into a $d_\ell \times d^\forall_{\backslash\ell}$ matrix. See Figure S1 for a graphical example of these definitions. $\mathbf{S}_\ell = \text{mat}_\ell\left[\{\mathcal{X}^\gamma\}_\gamma\right] \text{mat}_\ell\left[\{\mathcal{X}^\gamma\}_\gamma\right]^T$ is the covariance matrix for axis ℓ. Its top r_ℓ eigenvectors and eigenvalues are $\mathbf{V}_\ell^{(r_\ell)}$ and $\mathbf{E}_\ell^{(r_\ell)}$, respectively.

The final definition needed is that of the stridewise-blockwise trace $\text{tr}^a_b\left[\mathbf{X}\right]$; it arises in the gradient of terms involving Kronecker products. Letting $\mathbf{J}^{(ij)}$ is a matrix of zeros except for a single 1 in position (i, j), we have:

$$\text{tr}^a_b\left[\mathbf{X}\right]_{ij} = \text{tr}\left[\mathbf{X}\left(\mathbf{I}_{a\times a} \otimes \mathbf{J}^{(ij)} \otimes \mathbf{I}_{b\times b}\right)\right]$$

B Algorithm

Algorithm 1 **The improved GmGM algorithm**

Input: Dataset $\{\mathcal{D}^\gamma\}$, minimum eigenvalues $\{\epsilon_\ell\}$, sparsity $\{n_\ell\}$, principal components $\{r_\ell\}$
Output: $\{\mathbf{\Psi}_\ell\}$

for $1 \leq \ell \leq K$
  $\mathbf{D}_\ell \leftarrow \begin{bmatrix}\text{mat}_\ell\left[\mathcal{D}^{\gamma_1}\right] & \cdots & \text{mat}_\ell\left[\mathcal{D}^{\gamma_\Gamma}\right]\end{bmatrix}$
  $\mathbf{V}_\ell^{(r_\ell)} \leftarrow$ top r_ℓ left singular vectors of $\mathbf{D}_\ell$ ▷ Theorem 1
  $\mathbf{E}_\ell^{(r_\ell)} \leftarrow$ (top r_ℓ singular values of $\mathbf{D}_\ell)^2$
end for
$\mathbf{\Lambda}_\ell^{0,(r_\ell)} \leftarrow \begin{bmatrix}\frac{1}{E^{(r_\ell)}_{\ell_{11}}} & \cdots & \frac{1}{E^{(r_\ell)}_{\ell_{r_\ell r_\ell}}}\end{bmatrix}^T$
$\mu \leftarrow 1$
while not converged
  while $\exists\gamma \sum_{\ell\in\gamma} \min\mathbf{\Lambda}_\ell^{t+1,(r_\ell)} < \epsilon_\ell$ ▷ Ensure smallest eigenvalue is at least ϵ_ℓ
    Decrease μ if needed
    for $1 \leq \ell \leq K$ ▷ Gradient descent on eigenvalues
      $\mathbf{\Lambda}_\ell^{t+1,(r_\ell)} \leftarrow \mathbf{\Lambda}_\ell^{t,(r_\ell)} - \frac{\mu}{2}\left(\mathbf{E}_\ell^{(r_\ell)} - \sum_{\gamma|\ell\in\gamma} \text{tr}^{k^\gamma_{<\ell}}_{k^\gamma_{>\ell}}\left[\left(\bigoplus_{\ell'\in\gamma} \mathbf{\Lambda}_{\ell'}^{t,(r_{\ell'})}\right)^{-1}\right]\right)$
    end for
  end while
end while
for $1 \leq \ell \leq K$
  $\mathbf{\Psi}_\ell \leftarrow \text{thresh}_{n_\ell}\left[\mathbf{V}_\ell^{(r_\ell)} \mathbf{\Lambda}_\ell^{t,(r_\ell)} \mathbf{V}_\ell^{T,(r_\ell)}\right]$ ▷ Threshold simultaneously with recomposition
end for

Our maximum likelihood estimation algorithm (Algorithm 1) can be described as: calculate each $\mathbf{S}_\ell$, partially eigendecompose them, perform gradient descent on the eigenvalues[4], eigen-recompose to get $\mathbf{\Psi}_\ell$, and then threshold to get the final solution. However, creation of $\mathbf{S}_\ell$ requires $O(d_\ell^2)$ space - the goal is to only require $O(r_\ell d_\ell)$ space. This can be avoided by noting that:

$$\begin{aligned}
\mathbf{S}_\ell &= \sum_\gamma \mathbf{S}_\ell^\gamma \\
&= \sum_\gamma \text{mat}_\ell\left[\mathcal{D}^\gamma\right] \text{mat}_\ell\left[\mathcal{D}^\gamma\right]^T \\
&= \begin{bmatrix}\text{mat}_\ell\left[\mathcal{D}^1\right] & \ldots & \text{mat}_\ell\left[\mathcal{D}^\Gamma\right]\end{bmatrix} \begin{bmatrix}\text{mat}_\ell\left[\mathcal{D}^1\right]^T \\ \ldots \\ \text{mat}_\ell\left[\mathcal{D}^\Gamma\right]^T\end{bmatrix} \\
&\triangleq \text{mat}_\ell\left[\{\mathcal{D}^\gamma\}_\gamma\right] \text{mat}_\ell\left[\{\mathcal{D}^\gamma\}_\gamma\right]^T
\end{aligned}$$

$\mathbf{S}_\ell$ can be thought of as the covariance matrix for a $d_\ell \times \sum_\gamma d^\gamma_{\backslash\ell}$ matrix "$\text{mat}_\ell\left[\{\mathcal{D}^\gamma\}_\gamma\right]$", and hence its eigenvectors are precisely the singular vectors of $\text{mat}_\ell\left[\{\mathcal{D}^\gamma\}_\gamma\right]$. This allows avoiding ever constructing $\mathbf{S}_\ell$.

Such an approach has one major drawback; the Nonparanormal Skeptic works by supplying a rank-based $\mathbf{S}_\ell$ that, intuitively, represents the correlation matrix 'as if the data had come from a Gaussian'. To both avoid quadratic memory usage *and* weaken the normality assumption, it requires a modification. COCA [Han and Liu, 2014] is used to calculate nonparanormal eigenvectors; it works by replacing $\text{mat}_\ell\left[\{\mathcal{D}^\gamma\}_\gamma\right]$ with a matrix of ranks, mapping these ranks to a normal distribution, and then finding the singular vectors of this rank matrix.

[4] The formula for the gradient of the eigenvalues is derived in Appendix I.

Creating $\boldsymbol{\Psi}_\ell$ requires some care, as if we first 'eigen-recompose' our learned eigenvectors and eigenvalues before thresholding, we will have created a very large, dense matrix as an intermediate step. To avoid this, we create $\boldsymbol{\Psi}_\ell$ edge-by-edge, never storing edges that don't meet the threshold set.

The final practical issue to surmount is that of preserving input sparsity. $\text{mat}_\ell\left[\{\mathcal{D}^\gamma\}_\gamma\right]$ has $d_\forall$ elements, but in many contexts, such as transcriptomics, the dataset will be highly sparse; the majority of the input is full of zeros. Ideally, the matrix would never be 'densified'. Using COCA, however, requires computing the matrix of ranks, which will be fully dense. If unaddressed, this would enforce an $\Omega(d_\forall)$ minimum memory requirement. **Solving this is necessary for scalability**; as will be seen in Section 3.1, our algorithm can handle very large sparse datasets; if densified, they would be in excess of 30 GB of memory. In Appendix L, we show how to solve this using a rank-one update technique.

C Evidence for the low-rank approximability assumption

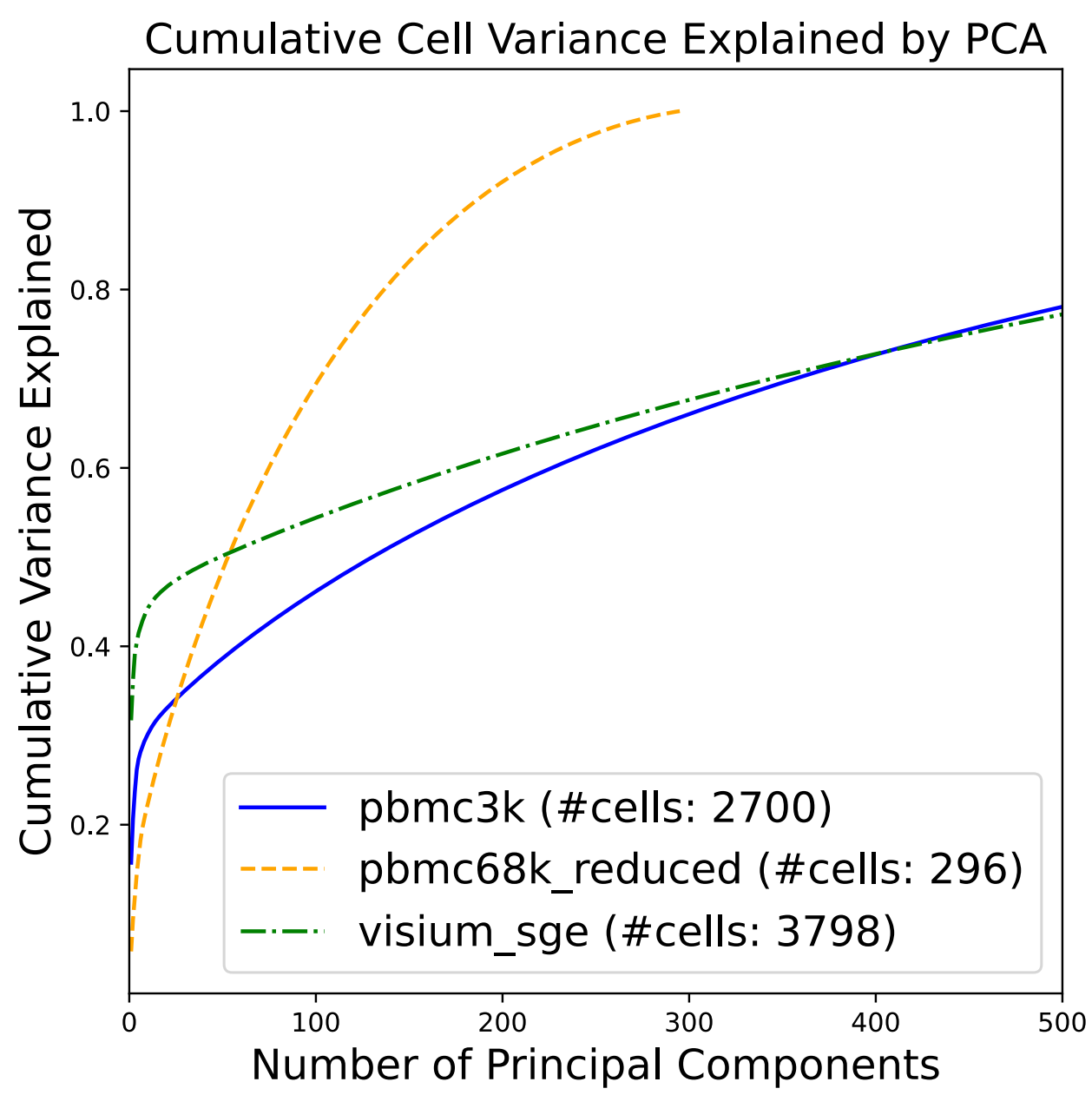

Fig. S2. The cumulative variance explained by low-rank approximations (PCA) for three scRNA-seq datasets.

The low-rank approximability assumption (Assumption 5) is frequently made in practice; whenever one performs PCA, this assumption is being made. This section aims to demonstrate the reasonability of Assumption 5. As PCA is likely to be most practitioner's primary encounter with low-rank approximations, we will frame our argument in terms of PCA.

The number of principal components to chose (and hence the rank of the low-rank approximation) is typically chosen by looking at the total explained variance of the components. An explained variance of 100% indicates perfect representation of the original matrix by the low-rank approximation. One method of picking the amount of principal components is to simply pick an amount that gives a satisfactory recovery rate, such as 60% or 80%. However, most components often represent random noise; we do not care about recovering them. An alternative selection procedure is to look for 'sharp corners' in the plot of cumulative variance, indicating the transition from informative to uninformative components. The first approach is liable to pick too many components, whereas the second approach is liable to pick too few. Finally, prior work is equivalent to keeping all the components; we only introduce Assumption 5 because keeping them all is not scalable. As such, a final perspective is to keep as many components as possible; with sufficient computing power, our approach then reduces to prior work.

Figure S2 displays the cumulative variance plot for three scRNA-seq datasets available in the ScanPy package and produced by 10X Genomics [Wolf et al., 2018]. Figure S2 shows that one does not need too many principal components to achieve good recovery; the explained variance approach requires a couple hundred components and the informative components approach requires less than a hundred for the larger datasets. This validates our use of low-rank approximations.

So far, we have justified Assumption 5 on the grounds of the dataset itself being low-rank. It is reasonable to wonder why we expect this to carry over to the graphs themselves being low-rank. There are two ways to see why this must be true.

Firstly, from a linear algebra perspective, the empirical covariance matrix $\boldsymbol{\Sigma} = \mathbf{X}\mathbf{X}^T$ is a product of (approximately) low-rank matrices $\mathbf{X}, \mathbf{X}^T$ by assumption, and hence itself must be (approximately) low rank. Thus, the (pseudo-)inverse (the precision matrix) is also low-rank. This is reflected in the structure of a Gaussian distribution - if the precision matrix is (approximately) low-rank then the resulting data drawn from it will also be (approximately) low rank.

For a more intuitive understanding, note that a low-ranked dataset implies that there are linear dependencies in the data. Thus, in some basis, there must be perfect correlations. Call the perfectly correlated objects x and y; as they are perfectly correlated, all their correlations with some third element z will be equal. Thus, there will be linear dependencies in the correlation matrix. If x and y are only approximately perfectly correlated, then their correlations with z will be approximately equal, and hence the correlation matrix will be approximately low-rank.

D Choice of Hyperparameters

Our model has two different hyperparameters, one governing the rank of the low-rank approximation and the other governing the sparsity of the resulting graphs. The choice of rank is equivalent to the choice of number of principal components, and as such there are a few different perspectives that could be taken. For example, one can choose the rank based on a preset 'explained variance' threshold, for example 80%. Alternatively, one could look for an 'elbow' in the scree plot (the plot of variance explained for each component).

A third perspective is to keep as many as possible. Prior work keeps all components: the reason this work introduces a low-rank approximation assumption is *because it is necessary for scalability*. For $d_1 \times d_2$ matrix data, we would recommend picking at most $\min(d_1, d_2)$ components under this perspective, as good algorithmic behavior is guaranteed by Theorem 2. More generally, for tensor-variate multi-modal data, we would recommend picking at most $\min\left(d^{\forall}_{\backslash\ell}\right)$.

One could also bring in prior domain knowledge to pick the rank. Intuitively, the rank represents the number of 'explanatory patterns' that the graph can be broken into (such patterns may represent communities or the local structure of a particularly influential hub node, for example). If one already has an idea as to how many independent processes might arise in the data (such as how many communities and key nodes there are), this could inform one's choice of rank.

The second hyperparameter controls the sparsity of the learned edges. If the dataset is very large, we would recommend keeping only the statistically significant edges (after adjustment). However, if the dataset is not very large, or if one is primarily interested only in clustering the network, we would recommend keeping the top k edges per vertex, where k is some small sensible default (like $k = 5$) that yields a fully connected graph. Thresholding this way guarantees there are no singletons in the graph, and is analogous to the construction of k-nearest-neighbors (kNN) graphs, which is already standard practice for cell clustering; it has also been applied to prior work on gene networks [Care et al., 2019].

E Derivation of the probability distribution

The space of all full-rank $d \times d$ matrices has dimension d^2. In contrast, the space of all rank-r $d \times d$ matrices has dimension $2dr - r^2$. Thus, any probability distribution over rank-r matrices must be degenerate, or 'singular', in the sense that the density is only positive on a subset of measure 0. First, let's consider a multivariate Gaussian of independent variables:

$$\mathbf{x} \sim \mathcal{N}\left(\mathbf{0}, \mathrm{diag}\left[\boldsymbol{\sigma}\right]\right)$$

$$\iff \qquad \mathrm{pdf}\left(\mathbf{x}\right) = \frac{\sqrt{\prod_i \frac{1}{\sigma_i}}}{(2\pi)^{\frac{r}{2}}} e^{-\frac{1}{2}\mathbf{x}^T \mathrm{diag}[\boldsymbol{\sigma}]^{-1}\mathbf{x}} \qquad (r \text{ Independent Normal Variables})$$

Suppose we then left-multiply $\mathbf{x}$ by an $r \times r$ orthonormal matrix $\mathbf{V}$ to introduce dependencies into our data while preserving Gaussianity. Letting $\mathbf{V}\mathrm{diag}\left[\boldsymbol{\sigma}\right]^{-1}\mathbf{V} = \boldsymbol{\Psi}$, we get:

$$\begin{aligned}\mathrm{pdf}\left(\mathbf{V}\mathbf{x}\right) &= \frac{\sqrt{\prod_i \frac{1}{\sigma_i}}}{(2\pi)^{\frac{r}{2}}} e^{-\frac{1}{2}\mathbf{x}^T \mathbf{V}\mathrm{diag}[\boldsymbol{\sigma}]^{-1}\mathbf{V}^T\mathbf{x}} \\ &= \frac{\sqrt{\det \boldsymbol{\Psi}}}{(2\pi)^{\frac{r}{2}}} e^{-\frac{1}{2}\mathbf{x}^T \boldsymbol{\Psi}\mathbf{x}}\end{aligned}$$

Notice that $\boldsymbol{\Psi}$ is fully general; $\mathbf{V}$ are the eigenvectors and $\boldsymbol{\sigma}^{-1}$ are the eigenvalues. To achieve a singular probability distribution, we multiply by a $d \times r$ orthonormal matrix $\mathbf{U}$ $(d > r)$ instead. The only noticeable change in the distribution's specification is the use of the pseudo-determinant (the product of only the non-zero eigenvalues of $\boldsymbol{\Psi} = \mathbf{U}\mathrm{diag}\left[\boldsymbol{\sigma}\right]\mathbf{U}^T$).

$$\mathrm{pdf}\left(\mathbf{U}\mathbf{x}\right) = \frac{\sqrt{\det^{\dagger} \boldsymbol{\Psi}}}{(2\pi)^{\frac{r}{2}}} e^{-\frac{1}{2}\mathbf{x}^T \boldsymbol{\Psi}\mathbf{x}}$$

The above formulas were for vector-variate $\mathbf{x}$; for tensor-variate $\mathcal{X}$ we can use the identity $\mathrm{vec}\left[\mathcal{X} \times_1 \mathbf{V}_1 \times_2 \cdots \times_K \mathbf{V}_K\right] = \left(\bigoplus_{\ell=K}^{1} \mathbf{V}_\ell\right)\mathrm{vec}\left[\mathcal{X}\right]$. It is then straightforward to present a Kronecker-sum-structured version of this distribution, the 'singular Kronecker-sum-structured normal distribution'. We let r_ℓ be the rank chosen for each axis, and $r^{\gamma}_{\forall}$ be the product of ranks of all axes occurring in modality γ.

$$\mathrm{pdf}_{\mathrm{normal}}(\mathcal{X}^\gamma) = \frac{\sqrt{\det^\dagger\left(\bigoplus_{\ell\in\gamma}\mathbf{\Psi}_\ell\right)}}{(2\pi)^{\frac{r_\vee^\gamma}{2}}} e^{-\frac{1}{2}\mathrm{vec}[\mathcal{X}^\gamma]^T\left(\bigoplus_{\ell\in\gamma}\mathbf{\Psi}_\ell\right)\mathrm{vec}[\mathcal{X}^\gamma]}$$
$$= \frac{\sqrt{\det^\dagger\left(\bigoplus_{\ell\in\gamma}\mathbf{\Psi}_\ell\right)}}{(2\pi)^{\frac{r_\vee^\gamma}{2}}} e^{-\frac{1}{2}\sum_{\ell\in\gamma}\mathrm{tr}[\mathbf{S}_\ell^\gamma\mathbf{\Psi}_\ell]}$$

When given a multi-modal dateset, we have:

$$\mathrm{pdf}_{\mathrm{normal}}\left(\{\mathcal{X}^\gamma\}\right) = \prod_\gamma \frac{\sqrt{\det^\dagger\left(\bigoplus_{\ell\in\gamma}\mathbf{\Psi}_\ell\right)}}{(2\pi)^{\frac{r_\vee^\gamma}{2}}} e^{-\frac{1}{2}\sum_{\ell\in\gamma}\mathrm{tr}[\mathbf{S}_\ell^\gamma\mathbf{\Psi}_\ell]}$$

F Identifiability

The Kronecker sum decomposition is typically parameterized by a non-identifiable representation of $\bigoplus_{\ell\in\gamma}\mathbf{\Psi}_\ell$. Let $\{c_\ell\}_{\ell\in\gamma}$ be a set of constants such that $\sum_{\ell\in\gamma} c_\ell = 0$, then:

$$\bigoplus_{\ell\in\gamma}(\mathbf{\Psi}_\ell + c_\ell\mathbf{I}_{d_\ell}) = \bigoplus_{\ell\in\gamma}\mathbf{\Psi}_\ell + \left(\sum_{\ell\in\gamma} c_\ell\right)\mathbf{I}_{d_\vee^\gamma} = \bigoplus_{\ell\in\gamma}\mathbf{\Psi}_\ell$$

This is not unique to the Kronecker sum model; all Kronecker-separable models experience some form of non-identifiability. However, this non-identifiability is rather benign. Firstly, it only affects the diagonals of our estimates; the off-diagonals (which encode the graph structure) are unaffected. Secondly, Greenewald et al. [2019] derived the following identifiable parameterization for the single-modality case:

$$\mathbf{\Omega}^\gamma = \left(\bigoplus_{\ell\in\gamma}\tilde{\mathbf{\Psi}}_\ell\right) + \tau^\gamma\mathbf{I}_{d_\vee^\gamma}$$
$$\textit{where}\ \ \mathrm{tr}\left[\tilde{\mathbf{\Psi}}_\ell\right] = 0$$

We can map the standard parameterization to this one with ease. As a shorthand, let $\tau_\ell = \mathrm{tr}\left[\mathbf{\Psi}_\ell\right]$:

$$\tau^\gamma = \sum_{\ell\in\gamma} d_{\setminus\ell}^\gamma \tau_\ell$$
$$\tilde{\mathbf{\Psi}}_\ell = \mathbf{\Psi}_\ell - \tau_\ell\mathbf{I}_{d_\ell}$$

However, Greenewald et al.'s parameterization does not apply to the multi-modality case. Ideally, we would wish to parameterize our dataset by the parameters $\{\tau^\gamma\}_\gamma$ and $\{\tilde{\mathbf{\Psi}}_\ell\}_\ell$, but there are linear dependencies in our parameterization which may prevent this.

Example 6. *Consider the bi-modal dataset where $\gamma_1 = (\ell_1, \ell_2)$ and $\gamma_2 = (\ell_1, \ell_2, \ell_3)$. There is a linear dependency in our trace parameters:*

$$\tau^{\gamma_1} = d_2\tau_1 + d_1\tau_2$$
$$\tau^{\gamma_2} = d_2d_3\tau_1 + d_1d_3\tau_2 + d_2d_3\tau_3$$
$$= d_3\tau^{\gamma_1} + d_2d_3\tau_3$$

In Example 6, the parameterization is still valid - we need both τ^{γ_1} and τ^{γ_2} to identify the parameterization. However, we also get the trace of the third axis, τ_3, as a byproduct. Unlike in the single modality case, in this case $\mathbf{\Psi}_3$ is fully-specified.

Example 7. *Consider the tri-modal dataset where $\gamma_1 = (\ell_1, \ell_2)$, $\gamma_2 = (\ell_3, \ell_4)$, and $\gamma_3 = (\ell_1, \ell_2, \ell_3, \ell_4)$. Then $\tau^{\gamma_3} = d_3d_4\tau^{\gamma_1} + d_1d_2\tau^{\gamma_2}$.*

Example 7 shows that Greenewald et al.'s parameterization can be over-determined in the multi-modal case. GmGM can be used on arbitrarily complicated multi-tensor-variate data; how can we know when a parameterization is over-determined or not?

Note that $\{\tau^{\gamma}\}$ are always a linear combination of τ_ℓ. Letting $d^{\gamma}_{\setminus\ell} = 0$ if $\ell \notin \gamma$, we can express this linear relationship as:

$$\begin{bmatrix} \tau^{\gamma_1} \\ \vdots \\ \tau^{\gamma_\Gamma} \end{bmatrix} = \mathbf{M}_{\boldsymbol{\gamma}} \begin{bmatrix} \tau_{\ell_1} \\ \vdots \\ \tau_{\ell_L} \end{bmatrix}$$

$$\mathbf{M}_{\boldsymbol{\gamma}} = \begin{bmatrix} d^{\gamma_1}_{\setminus\ell_1} & \cdots & d^{\gamma_1}_{\setminus\ell_L} \\ \vdots & \ddots & \vdots \\ d^{\gamma_\Gamma}_{\setminus\ell_1} & \cdots & d^{\gamma_\Gamma}_{\setminus\ell_L} \end{bmatrix}$$

Depending on the modalities and the lengths of each axis, $\mathbf{M}_{\boldsymbol{\gamma}}$ could be practically any $\Gamma \times L$ matrix with nonnegative integer coefficients, so it is hard to say much about it in general. However, we can make a few observations. In particular, $\mathbf{M}_{\boldsymbol{\gamma}}$ describes how our identifiable quantities $\{\tau^{\gamma}\}_{\gamma}$ are linked through the latent factors $\{\tau_\ell\}_\ell$. Zero-eigenvectors $\mathbf{x}$ of $\mathbf{M}_{\boldsymbol{\gamma}}$ correspond to the non-identifiabilities, as $\mathbf{M}_{\boldsymbol{\gamma}}(\mathbf{x} + \mathbf{y}) = \mathbf{M}_{\boldsymbol{\gamma}}\mathbf{y}$. Our parameter vector $\begin{bmatrix} \tau^{\gamma_1} & \cdots & \tau^{\gamma_\Gamma} \end{bmatrix}^T$ must lie in the *rowspace* of $\mathbf{M}_{\boldsymbol{\gamma}}$.

For most datasets, $\mathbf{M}_{\boldsymbol{\gamma}}$ has maximal row rank, in which case the approach of Greenewald et al. is unchanged, and we can parameterize by $\{\tau^{\gamma}\}_{\gamma}$. However, there are sometimes linear dependencies in $\{\tau^{\gamma}\}_{\gamma}$, causing us to choose a new, smaller parameterization that functions as a basis for this rowspace. Table S1 gives example parameterizations of the most common scenarios along with some unlikely-but-demonstrative ones.

In practice, the choice of parameterization is not too important; it does not have an effect on how we go about finding solutions, nor does it have an effect on the conditional dependency graph. However, it will be useful for hypothesis testing, which we will discuss in the next section.

Dataset	$\mathbf{M}_{\boldsymbol{\gamma}}$	Basis Vectors	Parameterization
$\gamma_1 = (\ell_1, \ell_2)$	$\begin{bmatrix} d_{\ell_2} & d_{\ell_1} \end{bmatrix}$	$\begin{bmatrix} \mathbf{e}_1 \end{bmatrix} = \begin{bmatrix} d_{\ell_2} & d_{\ell_1} \end{bmatrix}$	$\mathbf{\Omega}^{\gamma_1} = t_1 \mathbf{I}_{d_{\forall}^{\gamma_1}} + \bigotimes_{\ell \in \gamma_1} \tilde{\mathbf{\Psi}}_\ell$
$\gamma_1 = (\ell_1, ..., \ell_L)$	$\begin{bmatrix} d_{\setminus \ell_1} & \dots & d_{\setminus \ell_L} \end{bmatrix}$	$\begin{bmatrix} \mathbf{e}_1 \end{bmatrix} = \begin{bmatrix} d_{\setminus \ell_1} & \dots & d_{\setminus \ell_L} \end{bmatrix}$	$\mathbf{\Omega}^{\gamma_1} = t_1 \mathbf{I}_{d_{\forall}^{\gamma_1}} + \bigotimes_{\ell \in \gamma_1} \tilde{\mathbf{\Psi}}_\ell$
$\left\{ \gamma_i = (\ell_0, \ell_i) \right\}_{1 \leq i \leq \Gamma}$	$\begin{bmatrix} d_{\ell_1} & d_{\ell_0} & 0 & \dots \\ d_{\ell_2} & 0 & d_{\ell_0} & \dots \\ d_{\ell_3} & 0 & 0 & \dots \\ \vdots & \vdots & \vdots & \ddots \end{bmatrix}$	$\begin{bmatrix} \mathbf{e}_1 \\ \vdots \end{bmatrix} = \begin{bmatrix} d_{\ell_1} & d_{\ell_0} & 0 & \dots \\ d_{\ell_2} & 0 & d_{\ell_0} & \dots \\ d_{\ell_3} & 0 & 0 & \dots \\ \vdots & \vdots & \vdots & \ddots \end{bmatrix}$	$\begin{bmatrix} \mathbf{\Omega}^{\gamma_1} \\ \vdots \end{bmatrix} = \begin{bmatrix} t_1 \mathbf{I}_{d_{\forall}^{\gamma_1}} + \bigotimes_{\ell \in \gamma_1} \tilde{\mathbf{\Psi}}_\ell \\ \vdots \end{bmatrix}$
$\gamma_1 = (\ell_1, \ell_2)$ $\gamma_2 = (\ell_2, \ell_3)$ $\gamma_3 = (\ell_3, \ell_1)$	$\begin{bmatrix} d_{\ell_2} & d_{\ell_1} & 0 \\ 0 & d_{\ell_3} & d_{\ell_2} \\ d_{\ell_3} & 0 & d_{\ell_1} \end{bmatrix}$	$\begin{bmatrix} \mathbf{e}_1 \\ \mathbf{e}_2 \\ \mathbf{e}_3 \end{bmatrix} = \begin{bmatrix} d_{\ell_2} & d_{\ell_1} & 0 \\ 0 & d_{\ell_3} & d_{\ell_2} \\ d_{\ell_3} & 0 & d_{\ell_1} \end{bmatrix}$	$\begin{bmatrix} \mathbf{\Omega}^{\gamma_1} \\ \mathbf{\Omega}^{\gamma_2} \\ \mathbf{\Omega}^{\gamma_3} \end{bmatrix} = \begin{bmatrix} t_1 \mathbf{I}_{d_{\forall}^{\gamma_1}} + \bigotimes_{\ell \in \gamma_1} \tilde{\mathbf{\Psi}}_\ell \\ t_2 \mathbf{I}_{d_{\forall}^{\gamma_2}} + \bigotimes_{\ell \in \gamma_2} \tilde{\mathbf{\Psi}}_\ell \\ t_3 \mathbf{I}_{d_{\forall}^{\gamma_3}} + \bigotimes_{\ell \in \gamma_3} \tilde{\mathbf{\Psi}}_\ell \end{bmatrix}$
$\gamma_1 = (\ell_1, \ell_2)$ $\gamma_2 = (\ell_1, \ell_2, \ell_3)$ $\gamma_3 = (\ell_2, \ell_3, \ell_4)$	$\begin{bmatrix} d_{\ell_2} & d_{\ell_1} & 0 & 0 \\ d_{\ell_2} d_{\ell_3} & d_{\ell_1} d_{\ell_3} & d_{\ell_1} d_{\ell_2} & 0 \\ 0 & d_{\ell_3} d_{\ell_4} & d_{\ell_2} d_{\ell_4} & d_{\ell_2} d_{\ell_3} \end{bmatrix}$	$\begin{bmatrix} \mathbf{e}_1 \\ \mathbf{e}_2 \\ \mathbf{e}_3 \end{bmatrix} = \begin{bmatrix} d_{\ell_2} & d_{\ell_1} & 0 & 0 \\ d_{\ell_2} d_{\ell_3} & d_{\ell_1} d_{\ell_3} & d_{\ell_1} d_{\ell_2} & 0 \\ 0 & d_{\ell_3} d_{\ell_4} & d_{\ell_2} d_{\ell_4} & d_{\ell_2} d_{\ell_3} \end{bmatrix}$	$\begin{bmatrix} \mathbf{\Omega}^{\gamma_1} \\ \mathbf{\Omega}^{\gamma_2} \\ \mathbf{\Omega}^{\gamma_3} \end{bmatrix} = \begin{bmatrix} t_1 \mathbf{I}_{d_{\forall}^{\gamma_1}} + \bigotimes_{\ell \in \gamma_1} \tilde{\mathbf{\Psi}}_\ell \\ t_2 \mathbf{I}_{d_{\forall}^{\gamma_2}} + \bigotimes_{\ell \in \gamma_2} \tilde{\mathbf{\Psi}}_\ell \\ t_3 \mathbf{I}_{d_{\forall}^{\gamma_3}} + \bigotimes_{\ell \in \gamma_3} \tilde{\mathbf{\Psi}}_\ell \end{bmatrix}$
$\gamma_1 = (\ell_1, \ell_2)$ $\gamma_2 = (\ell_2, \ell_1)$	$\begin{bmatrix} d_{\ell_2} & d_{\ell_1} \\ d_{\ell_2} & d_{\ell_1} \end{bmatrix}$	$\begin{bmatrix} \mathbf{e}_1 \end{bmatrix} = \begin{bmatrix} d_{\ell_2} & d_{\ell_1} \end{bmatrix}$	$\begin{bmatrix} \mathbf{\Omega}^{\gamma_1} \\ \mathbf{\Omega}^{\gamma_2} \end{bmatrix} = \begin{bmatrix} t_1 \mathbf{I}_{d_{\forall}^{\gamma_1}} + \bigotimes_{\ell \in \gamma_1} \tilde{\mathbf{\Psi}}_\ell \\ t_1 \mathbf{I}_{d_{\forall}^{\gamma_2}} + \bigotimes_{\ell \in \gamma_2} \tilde{\mathbf{\Psi}}_\ell \end{bmatrix}$
$\gamma_1 = (\ell_1, \ell_2)$ $\gamma_2 = (\ell_3, \ell_4)$ $\gamma_3 = (\ell_1, \ell_2, \ell_3, \ell_4)$	$\begin{bmatrix} d_{\ell_2} & d_{\ell_1} & 0 & 0 \\ 0 & 0 & d_{\ell_4} & d_{\ell_3} \\ d_{\setminus \ell_1} & d_{\setminus \ell_2} & d_{\setminus \ell_3} & d_{\setminus \ell_4} \end{bmatrix}$	$\begin{bmatrix} \mathbf{e}_1 \\ \mathbf{e}_2 \end{bmatrix} = \begin{bmatrix} d_{\ell_2} & d_{\ell_1} & 0 & 0 \\ 0 & 0 & d_{\ell_4} & d_{\ell_3} \end{bmatrix}$	$\begin{bmatrix} t_1 \mathbf{I}_{d_{\forall}^{\gamma_1}} + \bigotimes_{\ell \in \gamma_1} \tilde{\mathbf{\Psi}}_\ell \\ t_2 \mathbf{I}_{d_{\forall}^{\gamma_2}} + \bigotimes_{\ell \in \gamma_2} \tilde{\mathbf{\Psi}}_\ell \\ (d_{\ell_3} d_{\ell_4} t_1 + d_{\ell_1} d_{\ell_2} t_2) \mathbf{I}_{d_{\forall}^{\gamma_2}} + \bigotimes_{\ell \in \gamma_2} \tilde{\mathbf{\Psi}}_\ell \end{bmatrix}$

Table S1. Identifiable parameterizations for several datasets. Here, t_i is the coefficient corresponding to basis vector $\mathbf{e}_i$, i.e, t_i is one of the parameters, along with $\tilde{\mathbf{\Psi}}_\ell$. Often, $t_i = \tau^{\gamma_i}$. Note that many different bases are possible; our selection here is somewhat arbitrary. As can be seen, typically $\mathbf{M}_\gamma$ itself can be used for the basis vectors, but sometimes there can be linear dependencies in the data. The first three rows in this matrix correspond to the cases most likely to arise in practice; a matrix-variate dataset, a tensor-variate dataset, and a multi-modality matrix-variate dataset with a consistently shared axis. Cells with a grey background indicate a smaller parameterization than that of Greenewald et al..

G Computational complexity

Calculating the top r_ℓ singular vectors for each axis's $d_\ell \times d_{\forall\setminus\ell}$ matrix $\mathbf{D}_\ell$ takes $O(\sum_\ell r_\ell d_{\forall|\ell})$ time and $O(\sum_\ell r_\ell d_\ell)$ space. Per iteration, the convex optimization step requires computing $\left(\bigoplus_{\ell\in\gamma} \mathbf{\Lambda}_\ell^{(r_\ell)}\right)^{-1}$, which takes $O(\sum_\gamma \prod_{\ell\in\gamma} r_\ell)$ time and space. The eigen-recomposition step takes $O(\sum_\ell r_\ell d_\ell^2)$ time, with thresholding talking $O(\sum d_\ell^2)$ time - although our implementation when not thresholding by significance takes $O(\sum_\ell d_\ell^2 \log n_\ell)$ to minimize the number of times we pass through the data. As we threshold and eigen-recompose simultaneously, it requires $O(n_\ell)$ space.

The Nonparanormal Skeptic requires ranking the data, taking $O(\sum_\ell d_{\forall|\ell} \log d_\ell)$ time and $O(sd_\forall)$ space, where s is the sparsity of the input dataset. This leads to an algorithm with the following complexity:

$$O\left(\sum_\gamma \prod_{\ell\in\gamma} r_\ell + \sum_\ell \left(r_\ell d_{\forall|\ell} + r_\ell d_\ell^2 \log n_\ell + d_{\forall|\ell} \log d_\ell\right)\right) \quad \text{(time)}$$

$$O\left(\sum_\gamma \prod_{\ell\in\gamma} r_\ell + \sum_\ell (r_\ell d_\ell + n_\ell) + sd_\forall\right) \quad \text{(space)}$$

Blue terms arise only if using the Nonparanormal Skeptic, and the grey term is a product of our implementation; it could be dropped, but would in practice make the algorithm slower. As this formula is complicated, we will give an example of a special case. In the case of matrix-variate data with $n_\ell \in O(d_\ell)$, and constant r_ℓ (the case for many real-world datasets), as well as for simplicity assuming $d = d_{\ell_1} \approx d_{\ell_2}$ and thresholding by statistical significance, these become:

$$O\left(d^2 + d^2 \log d\right) \quad \text{(time)}$$

$$O\left(d + sd^2\right) \quad \text{(space)}$$

In such a scenario, the optimal runtime would be $\Omega\left(sd^2\right)$ as it must visit every element at least once. Use of the Nonparanormal Skeptic increases the asymptotics, but as long as the dataset is sparse such an increase is modest.

H Kronecker product and stridewise-blockwise trace lemmas

This section contains the derivation of a few lemmas pertaining to Kronecker products. The stridewise-blockwise trace often appears in derivatives involving Kronecker products, and thus is integral to our method as well. The first two are known from prior work.

Lemma (Dahl et al. [2013] Lemma 1; Extraction Property)**.**

$$\mathrm{tr}\left[\left(\mathbf{I}_{a\times a} \otimes \mathbf{X} \otimes \mathbf{I}_{b\times b}\right)\mathbf{M}\right] = \mathrm{tr}\left[\mathbf{X}\mathrm{tr}_b^a\left[\mathbf{M}\right]\right]$$

Lemma (Cyclic Property [Andrew et al., 2024])**.**

$$\mathrm{tr}_b^a\left[\left(\mathbf{A}_{a\times a} \otimes I \otimes \mathbf{B}_{b\times b}\right)\mathbf{M}\right] = \mathrm{tr}_b^a\left[\mathbf{M}\left(\mathbf{A}_{a\times a} \otimes I \otimes \mathbf{B}_{b\times b}\right)\right]$$

The next lemma is a generalized version of lemmas from prior work; an 'extraction' property (a generalized Lemma 2 from Dahl et al. [2013]).

Lemma 1 (Extraction Property)**.**

$$\mathrm{tr}_b^a\left[\left(\mathbf{I}_a \otimes \mathbf{X} \otimes \mathbf{I}_b\right)\mathbf{M}\left(\mathbf{I}_a \otimes \mathbf{Y}^T \otimes \mathbf{I}_b\right)\right] = \mathbf{X}\mathrm{tr}_b^a\left[\mathbf{M}\right]\mathbf{Y}^T$$

Proof This proof follows the original by Dahl et al. [2013] closely; the only differences are that it is proven in the tensor-variate case and that $\mathbf{X}$ and $\mathbf{Y}$ are not constrained to be the same. The differences do not significantly affect the proof.

$$\begin{aligned}
\mathrm{tr}_b^a\left[\left(\mathbf{I}_a \otimes \mathbf{X} \otimes \mathbf{I}_b\right)\mathbf{M}\left(\mathbf{I}_a \otimes \mathbf{Y}^T \otimes \mathbf{I}_b\right)\right] &= \mathrm{tr}\left[\left(\mathbf{I}_a \otimes \mathbf{X} \otimes \mathbf{I}_b\right)\mathbf{M}\left(\mathbf{I}_a \otimes \mathbf{Y}^T \otimes \mathbf{I}_b\right)\left(\mathbf{I}_a \otimes \mathbf{J}^{ij} \otimes \mathbf{I}_b\right)\right]_{ij} \\
&= \mathrm{tr}\left[\left(\mathbf{I}_a \otimes \mathbf{j}^j\mathbf{X} \otimes \mathbf{I}_b\right)\mathbf{M}\left(\mathbf{I}_a \otimes \mathbf{Y}^T\mathbf{j}^{i,T} \otimes \mathbf{I}_b\right)\right]_{ij} \\
&= \mathrm{tr}\left[\left(\mathbf{I}_a \otimes \mathbf{X}_j \otimes \mathbf{I}_b\right)\mathbf{M}\left(\mathbf{I}_a \otimes \mathbf{Y}_i^T \otimes \mathbf{I}_b\right)\right]_{ij} \\
&= \mathrm{tr}\left[\left(\mathbf{I}_a \otimes \otimes \mathbf{Y}_i^T\mathbf{X}_j \otimes \mathbf{I}_b\right)\mathbf{M}\right]_{ij} \\
&= \mathrm{tr}\left[\mathbf{Y}_i^T\mathbf{X}_j\mathrm{tr}_b^a\left[\mathbf{M}\right]\right]_{ij} \\
&= \mathrm{tr}\left[\mathbf{X}_j\mathrm{tr}_b^a\left[\mathbf{M}\right]\mathbf{Y}_i^T\right]_{ij}
\end{aligned}$$

Note now that the value inside the trace is a scalar value, and hence we can drop the outer trace.

$$\mathrm{tr}_b^a\left[\left(\mathbf{I}_a \otimes \mathbf{X} \otimes \mathbf{I}_b\right)\mathbf{M}\left(\mathbf{I}_a \otimes \mathbf{Y}^T \otimes \mathbf{I}_b\right)\right] = \left[\mathbf{X}_j\mathrm{tr}_b^a\left[\mathbf{M}\right]\mathbf{Y}_i^T\right]_{ij}$$

Which leads us to the final result.

$$\mathrm{tr}_b^a\left[\left(\mathbf{I}_a \otimes \mathbf{X} \otimes \mathbf{I}_b\right)\mathbf{M}\left(\mathbf{I}_a \otimes \mathbf{Y}^T \otimes \mathbf{I}_b\right)\right] = \mathbf{X}\mathrm{tr}_b^a\left[\mathbf{M}\right]\mathbf{Y}^T$$

□

Finally, we need a new property of the stridewise-blockwise trace, the 'Downsampling Property'. This is essential to be able to work with rectangular matrices in the stridewise-blockwise trace, such as those arising from partial eigendecomposition.

Lemma 2 (Downsampling Property)**.** *Suppose* $\mathbf{U}_{a\times x}$ *and* $\mathbf{V}_{a\times x}$ *are matrices such that* $\mathbf{V}^T\mathbf{U} = \mathbf{I}_{x\times x}$*, and* $\mathbf{W}_{b\times y}$ *and* $\mathbf{X}_{b\times y}$ *are matrices such that* $\mathbf{X}^T\mathbf{W} = \mathbf{I}_{y\times y}$*. Then we have the following.*

$$\mathrm{tr}_b^a\left[\left(\mathbf{U} \otimes \mathbf{I} \otimes \mathbf{W}\right)\mathbf{M}\left(\mathbf{V} \otimes \mathbf{I} \otimes \mathbf{X}\right)^T\right] = \mathrm{tr}_y^x\left[\mathbf{M}\right]$$

Proof

$$\begin{aligned}
\mathrm{tr}_b^a\left[\left(\mathbf{U} \otimes \mathbf{I} \otimes \mathbf{W}\right)\mathbf{M}\left(\mathbf{V} \otimes \mathbf{I} \otimes \mathbf{X}\right)^T\right] &= \mathrm{tr}\left[\left(\mathbf{U} \otimes \mathbf{I} \otimes \mathbf{W}\right)\mathbf{M}\left(\mathbf{V} \otimes \mathbf{I} \otimes \mathbf{X}\right)^T\left(\mathbf{I} \otimes \mathbf{J}^{ij} \otimes \mathbf{I}\right)\right]_{ij} \\
&= \mathrm{tr}\left[\mathbf{M}\left(\mathbf{V}^T\mathbf{U} \otimes \mathbf{J}^{ij} \otimes \mathbf{X}^T\mathbf{W}\right)\right]_{ij} \\
&= \mathrm{tr}\left[\mathbf{M}\left(\mathbf{I}_{x\times x} \otimes \mathbf{J}^{ij} \otimes \mathbf{I}_{y\times y}\right)\right]_{ij} \\
&= \mathrm{tr}_y^x\left[\mathbf{M}\right]
\end{aligned}$$

□

I Finding the maximum likelihood estimator

In this section, we will derive gradients for gradient descent and prove Theorem 1. For convenience, we repeat the definition of the NLL.

$$\mathrm{NLL} = \frac{-1}{2}\sum_{\gamma}\left(\log\det\left[\mathbf{t}^T\mathbf{a}^\gamma\mathbf{I}_{d_\forall^\gamma} + \bigoplus_{\ell\in\gamma}\mathbf{\Psi}_\ell\right] - \sum_{\ell\in\gamma}\mathrm{tr}\left[\mathbf{S}_\ell^\gamma\mathbf{\Psi}_\ell\right] - \sum_{\ell\in\gamma}\frac{\mathbf{t}^T\mathbf{a}^\gamma}{L^\gamma}\mathrm{tr}\left[\mathbf{S}_\ell^\gamma\right]\right)$$

To derive the MLE eigenvectors, we proceed in a manner similar to Andrew et al. [2024]. A key difference is that we must now differentiate a log-pseudodeterminant, although it behaves analagously to the derivative of a full determinant. This is given by Theorem 2.15 of Holbrook [2018]:

$$\frac{\partial}{\partial \mathbf{A}} \text{logdet}^{\dagger}\left(\mathbf{A}\right) = \text{tr}\left[\mathbf{A}^{\dagger}\right]$$

Lemma 3. *At the maximum likelihood of the singular Kronecker-sum-structured normal distribution, we have that* $\mathbf{S}_\ell = \sum_{\gamma|\ell\in\gamma} \text{tr}_{d^\gamma_{>\ell}}^{d^\gamma_{<\ell}}\left[\left(\bigoplus_{\ell'\in\gamma}\mathbf{\Psi}_{\ell'}\right)^{\dagger}\right]$.

Proof Note that $\mathbf{\Psi}_\ell$ is symmetric, and hence $\frac{\partial f(\mathbf{\Psi})}{\partial \mathbf{\Psi}} = \text{sym}\left[\mathbf{X}\right] = \frac{\mathbf{X}+\mathbf{X}^T}{2}$, where $\mathbf{X}$ is the derivative found without taking into account symmetry [Srinivasan and Panda, 2023]. It does not affect the location of the MLE (although we include it in the derivations for completeness), but it does affect the value of the gradient, and thus will be important to account for when the gradient itself is of interest.

$$\begin{aligned}
\frac{\partial}{\partial \mathbf{\Psi}_\ell}\text{NLL} &= \text{sym}\left[\frac{\partial}{\partial \mathbf{\Psi}_\ell}\sum_{\ell'}\frac{1}{2}\text{tr}\left[\mathbf{S}_{\ell'}\mathbf{\Psi}_{\ell'}\right] - \sum_{\gamma}\left(\frac{\partial}{\partial \mathbf{\Psi}_{\ell_{ij}}}\frac{1}{2}\text{logdet}^{\dagger}\left[\bigoplus_{\ell'\in\gamma}\mathbf{\Psi}_{\ell'}\right]_{ij}\right)\right] \\
&= \text{sym}\left[\sum_{\gamma}\frac{1}{2}\mathbf{S}^{\gamma}_{\ell} - \frac{1}{2}\sum_{\gamma|\ell\in\gamma}\text{tr}\left[\left(\bigoplus_{\ell'\in\gamma}\mathbf{\Psi}_{\ell'}\right)^{\dagger}\frac{\partial}{\partial \mathbf{\Psi}_{\ell_{ij}}}\bigoplus_{\ell'\in\gamma}\mathbf{\Psi}_{\ell'}\right]_{ij}\right]
\end{aligned}$$

It is also not too hard to show that $\frac{\partial}{\partial \mathbf{\Psi}_{\ell_{ij}}}\bigoplus_{\ell'\in\gamma}\mathbf{\Psi}_{\ell'} = \mathbf{I}_{d^\gamma_{<\ell}}\otimes\mathbf{J}^{ij}_{d_\ell\times d_\ell}\otimes\mathbf{I}_{d^\gamma_{>\ell}}$ (not accounting for symmetry).

$$\begin{aligned}
\frac{\partial}{\partial \mathbf{\Psi}_{\ell_{ij}}}\bigoplus_{\ell'\in\gamma}\mathbf{\Psi}_{\ell'} &= \frac{\partial}{\partial \mathbf{\Psi}_{\ell_{ij}}}\sum_{\ell'\in\gamma}\mathbf{I}_{d^\gamma_{<\ell'}}\otimes\mathbf{\Psi}_{\ell'}\otimes\mathbf{I}_{d^\gamma_{>\ell'}} \\
&= \mathbf{I}_{d^\gamma_{<\ell}}\otimes\frac{\partial}{\partial \mathbf{\Psi}_{\ell_{ij}}}\mathbf{\Psi}_{\ell}\otimes\mathbf{I}_{d^\gamma_{>\ell}} \\
&= \mathbf{I}_{d^\gamma_{<\ell}}\otimes\mathbf{J}^{ij}_{d_\ell\times d_\ell}\otimes\mathbf{I}_{d^\gamma_{>\ell}}
\end{aligned}$$

By the definition of the stridewise-blockwise trace, we have that:

$$\frac{\partial}{\partial \mathbf{\Psi}_\ell}\text{NLL} = \text{sym}\left[\sum_{\gamma}\frac{1}{2}\mathbf{S}^{\gamma}_{\ell} - \frac{1}{2}\sum_{\gamma|\ell\in\gamma}\text{tr}_{d^\gamma_{>\ell}}^{d^\gamma_{<\ell}}\left[\left(\bigoplus_{\ell'\in\gamma}\mathbf{\Psi}_{\ell'}\right)^{\dagger}\right]\right]$$

Now, note that the maximum likelihood corresponds to the point where $\frac{\partial}{\partial \mathbf{\Psi}_\ell}\text{NLL}$ is 0.

$$\mathbf{S}_\ell = \sum_{\gamma|\ell\in\gamma}\text{tr}_{d^\gamma_{>\ell}}^{d^\gamma_{<\ell}}\left[\left(\bigoplus_{\ell'\in\gamma}\mathbf{\Psi}_{\ell'}\right)^{\dagger}\right]$$

□

Theorem (Theorem 1)**.** *Let the rank-*r_ℓ *partial eigendecomposition of* $\mathbf{S}_\ell$ *be* $\mathbf{V}^{(k)}_\ell\mathbf{E}^{(r_\ell)}_\ell\mathbf{V}^{(r_\ell),T}_\ell$*, with* $r_\ell \geq \max\left(\text{rank}\left[\mathbf{S}_\ell\right], \text{rank}\left[\mathbf{\Psi}_\ell\right]\right)$*. Then* $\mathbf{V}^{(r_\ell)}_\ell$ *are also eigenvectors of* $\mathbf{\Psi}_\ell$*.*

Proof Suppose we had a rank-r_ℓ partial eigendecomposition yielding a $d_\ell\times r_\ell$ matrix of eigenvectors $\mathbf{V}^{(r_\ell)}_\ell$; in this case, $\mathbf{V}^{(r_\ell),T}_\ell\mathbf{V}^{(r_\ell)}_\ell$ is still the identity matrix (albeit of a smaller dimension); this will allow us to use Lemma 2 to 'downsample' the stridewise-blockwise trace.

We will use $\mathbf{V}^{\gamma}_{<\ell}$ as a shorthand for $\bigotimes_{\ell'\in\gamma|\ell'<^\gamma\ell}\mathbf{V}^{(r'_\ell)}_{\ell'}$ (with $\mathbf{V}^{\gamma}_{>\ell}$ defined analogously).

With this in mind, note that by Lemma 3 we have that:

$$\mathbf{S}_\ell = \sum_{\gamma|\ell\in\gamma} \mathrm{tr}^{d^\gamma_{<\ell}}_{d^\gamma_{>\ell}}\left[\left(\bigoplus_{\ell'\in\gamma}\mathbf{\Psi}_{\ell'}\right)^\dagger\right]$$
$$= \sum_{\gamma|\ell\in\gamma} \mathrm{tr}^{d_{<\ell}}_{d^\gamma_{>\ell}}\left[\left(\mathbf{V}^\gamma_{<\ell}\otimes\mathbf{V}^{(r_\ell)}_\ell\otimes\mathbf{V}^\gamma_{>\ell}\right)\left(\bigoplus_{\ell'\in\gamma}\mathbf{\Lambda}^{(r_{\ell'})}_{\ell'}\right)^\dagger\left(\mathbf{V}^\gamma_{<\ell}\otimes\mathbf{V}^{(r_\ell)}_\ell\otimes\mathbf{V}^\gamma_{>\ell}\right)^T\right]$$
$$= \mathbf{V}^{(r_\ell)}_\ell\sum_{\gamma|\ell\in\gamma} \mathrm{tr}^{k^\gamma_{<\ell}}_{k^\gamma_{>\ell}}\left[\left(\bigoplus_{\ell'\in\gamma}\mathbf{\Lambda}^{(r_{\ell'})}_{\ell'}\right)^\dagger\right]\mathbf{V}^{(r_\ell),T}_\ell \qquad \text{(Extraction and Downsampling Properties.)}$$

The $\mathbf{V}^\gamma_{<\ell}$ and $\mathbf{V}^\gamma_{>\ell}$ matrices depend on γ as the precise tensor they are in affects which ℓs are present and in what order. Thankfully, they disappear due to the downsampling property of sb-trace, allowing us to express our equation as the product of orthogonal matrices $\mathbf{V}^{(r_\ell)}_\ell, \mathbf{V}^{(r_\ell),T}_\ell$ and a diagonal matrix $\sum_{\gamma|\ell\in\gamma}\mathrm{tr}^{k^\gamma_{<\ell}}_{k^\gamma_{>\ell}}\left[\left(\bigoplus_{\ell'\in\gamma}\mathbf{\Lambda}^{(r_{\ell'})}_{\ell'}\right)^\dagger\right]$. Thus, this is clearly a partial eigendecomposition of $\mathbf{S}_\ell$, completing the proof. □

Theorem 1 establishes that we can solve for the eigenvectors of each $\mathbf{\Psi}_\ell$ analytically. No such expression for the eigenvalues exist, but it is not too hard to find them with gradient descent. The proportion of time this estimation takes is negligible, due to cost of partial eigendecomposition and the comparatively small dimension of the eigenvalues ($O(\sum_\ell d_\ell)$ parameters rather than $O(\sum_\ell d_\ell^2)$). We derive the gradient below:

Proposition. *The gradient of the* NLL *with respect to the eigenvalues is given by* $\frac{1}{2}\left(\mathbf{E}^{(r_\ell)}_\ell - \sum_{\gamma|\ell\in\gamma}\mathrm{tr}^{k^\gamma_{<\ell}}_{k^\gamma_{>\ell}}\left[\left(\bigoplus_{\ell'\in\gamma}\mathbf{\Lambda}^{(r_{\ell'})}_{\ell'}\right)^\dagger\right]\right)$.

Proof As mentioned earlier, we will use the functional invariance of the MLE to change the problem from finding when $\frac{\partial}{\partial\mathbf{\Psi}_\ell}\mathrm{NLL}=0$ to one of finding when:

$$\frac{\partial}{\partial\mathbf{V}^{(r_\ell)}_\ell}\mathrm{NLL}=0$$
$$\frac{\partial}{\partial\mathbf{\Lambda}^{(r_\ell)}_\ell}\mathrm{NLL}=0$$

We already know the conditions on $\mathbf{V}^{(r_\ell)}_\ell$ at the MLE, thanks to Theorem 1, so we will focus on $\mathbf{\Lambda}^{(r_\ell)}_\ell$. We follow a similar derivation to Lemma 3.

$$\frac{\partial}{\partial\mathbf{\Lambda}_\ell}\mathrm{NLL} = \frac{\partial}{\partial\mathbf{\Lambda}^{(r_\ell)}_\ell}\sum_{\ell'}\frac{1}{2}\mathrm{tr}\left[\mathbf{S}_{\ell'}\mathbf{\Psi}_{\ell'}\right] - \sum_\gamma\left(\frac{\partial}{\partial\mathbf{\Lambda}^{(r_\ell)}_{\ell_{ii}}}\frac{1}{2}\mathrm{logdet}^\dagger\left[\bigoplus_{\ell'\in\gamma}\mathbf{\Psi}_{\ell'}\right]_{ii}\right)$$
$$= \frac{\partial}{\partial\mathbf{\Lambda}^{(r_\ell)}_\ell}\sum_{\ell'}\frac{1}{2}\mathrm{tr}\left[\mathbf{E}^{(r_{\ell'})}_{\ell'}\mathbf{\Lambda}^{(r_{\ell'})}_{\ell'}\right] - \sum_\gamma\left(\frac{\partial}{\partial\mathbf{\Lambda}^{(r_\ell)}_{\ell_{ii}}}\frac{1}{2}\mathrm{logdet}^\dagger\left[\bigoplus_{\ell'\in\gamma}\mathbf{\Lambda}^{(r_{\ell'})}_{\ell'}\right]_{ii}\right)$$
$$= \sum_\gamma\frac{1}{2}\mathbf{E}^{(r_\ell),\gamma}_\ell - \frac{1}{2}\sum_{\gamma|\ell\in\gamma}\mathrm{tr}\left[\left(\bigoplus_{\ell'\in\gamma}\mathbf{\Lambda}^{(r_{\ell'})}_{\ell'}\right)^\dagger\frac{\partial}{\partial\mathbf{\Lambda}^{(r_\ell)}_{\ell_{ii}}}\bigoplus_{\ell'\in\gamma}\mathbf{\Lambda}^{(r_{\ell'})}_{\ell'}\right]_{ii}$$
$$= \frac{1}{2}\left(\mathbf{E}^{(r_\ell)}_\ell - \sum_{\gamma|\ell\in\gamma}\mathrm{tr}^{k^\gamma_{<\ell}}_{k^\gamma_{>\ell}}\left[\left(\bigoplus_{\ell'\in\gamma}\mathbf{\Lambda}^{(r_{\ell'})}_{\ell'}\right)^\dagger\right]\right)$$

In the derivation, we made use of the fact that $\mathbf{S}_\ell$ and $\mathbf{\Psi}_\ell$ had the same eigenvectors at the MLE, the cyclic property of the trace, and the fact that pseudodeterminants only depend on the nonzero eigenvalues. □

J Existence and uniqueness

It is easy to see that our objective is convex; unstructured Gaussian precision matrix estimation is strictly convex, and the space of Kronecker-sum-decomposable matrices is also convex. In this section, we will further prove the existence and uniqueness of solutions (Theorem 2).

Lemma 4 (Existence)**.** *There exists a minimizer of the negative log likelihood in the domain* $\mathbf{\Lambda}_\ell^{(r_\ell)} \geq \epsilon_\ell$ *(Assumption 4), where* ϵ_ℓ *is some strictly positive constant (for each* ℓ*), as long as* $\mathbf{E}_\ell^{(r_\ell)} \in \mathbb{R}_{r_\ell}^{++}$ *(i.e.* $r_\ell \leq \operatorname{rank}[\mathbf{S}_\ell]$*).*

Proof First, note that $\bigtimes_\ell \bigtimes_{1 \leq i \leq r_\ell} [\epsilon_\ell, \infty]$ is a compact subset of the extended reals, and thus a minimum to any continuous function must exist on this set. As it is convex, it will be a global minimum. However, the minimum could exist at ∞, i.e., only in the limit. To rule out this case, we will establish an upper bound on the solution.

Recall that any minimum must satisfy $\mathbf{E}_\ell^{(r_\ell)} = \sum_{\gamma | \ell \in \gamma} \operatorname{tr}_{k_{>\ell}^\gamma}^{k_{<\ell}^\gamma}\left[\left(\oplus_{\ell' \in \gamma} \mathbf{\Lambda}_{\ell'}^{(r_{\ell'})}\right)^\dagger\right]$ (for each ℓ). It will be helpful to see what this formula equates to elementwise:

$$
\begin{aligned}
\mathbf{E}_{\ell_{ii}}^{(r_\ell)} &= \sum_{\gamma | \ell \in \gamma} \operatorname{tr}_{d_{>\ell}^\gamma}^{d_{<\ell}^\gamma}\left[\left(\oplus_{\ell' \in \gamma} \mathbf{\Lambda}_{\ell'}^{(r_{\ell'})}\right)^\dagger\right]_{ii} \\
&= \sum_{\gamma | \ell \in \gamma} \operatorname{tr}\left[\left(\oplus_{\ell' \in \gamma} \mathbf{\Lambda}_{\ell'}^{(r_{\ell'})}\right)^\dagger \left(\mathbf{I}_{r_{<\ell}^\gamma} \otimes \mathbf{J}^{ii} \otimes \mathbf{I}_{r_{>\ell}^\gamma}\right)\right] \\
&= \sum_{\gamma | \ell \in \gamma} \sum_{\mathbf{j} \text{ indexing all non-}\ell \text{ axes}} \frac{1}{\lambda_{\ell_i} + \sum_{\ell'} \lambda_{\ell'_{j_{\ell'}}}}
\end{aligned}
$$

Thus, every element of $\mathbf{E}_\ell^{(r_\ell)}$ has a corresponding element of $\mathbf{\Lambda}_\ell^{(r_\ell)}$ that appears in all summands composing it, and vice versa. Suppose $\lambda_{\ell_i} = \frac{\sum_\gamma d_{\setminus \ell}^\gamma}{\mathbf{E}_{\ell_{ii}}}$ (for a given ℓ, and every i). Note that if for all other ℓ', $\lambda_{\ell'_{j_{\ell'}}}$ were 0, then the sum of these λ_ℓ terms would sum to exactly $\mathbf{E}_{\ell_{ii}}$. However, as $\mathbf{\Lambda}_\ell^{(r_\ell)} \geq \epsilon_\ell$, each reciprocal is smaller than the case where they are 0; thus, they cannot add up to $\mathbf{E}_{\ell_{ii}}$.

As λ_{ℓ_i} increases above this bound, the problem only becomes more severe. This is easy to see by checking the gradient, which indicates that the negative log-likelihood increases as one increases λ_{ℓ_i}.

$$
\begin{aligned}
\frac{\partial}{\partial \mathbf{\Lambda}_{\ell_{ii}}^{(r_\ell)}} \mathrm{NLL} &= \frac{1}{2}\left(\mathbf{E}_{\ell_{ii}} - \sum_{\gamma | \ell \in \gamma} \sum_{\mathbf{j} \text{ indexing all non-}\ell \text{ axes}} \frac{1}{\lambda_{\ell_i} + \sum_{\ell'} \lambda_{\ell'_{j_{\ell'}}}}\right) \\
&> \frac{1}{2}\left(\mathbf{E}_{\ell_{ii}} - \mathbf{E}_{\ell_{ii}}\right) \\
&= 0
\end{aligned}
$$

This implies that the minimum must occur between $\frac{\sum_\gamma d_{\setminus \ell}^\gamma}{\mathbf{E}_{\ell_{ii}}} \geq \lambda_{\ell_{ii}} \geq \epsilon_\ell$. Clearly, this rules out the unsavory case of the minimum occurring in the limit. This completes the proof. □

We already saw in Section F that the diagonals of $\mathbf{\Psi}_\ell$ are not identifiable. However, this non-identifiability is only an effect of our parameterization of the full precision matrices $\{\mathbf{\Omega}^\gamma\}$; it is reasonable to ask if the solution is unique up to this non-identifiability; Lemma 5 shows that this is true.

Lemma 5 (Uniqueness up-to Identifiability)**.** *The solution of our optimization problem is unique, except for the non-identifiability in the diagonals.*

Proof As mentioned earlier, we can frame our optimization problem as the strictly convex problem of unstructured precision matrix estimation (as in the graphical lasso problem), subject to a (convex) Kronecker-sum-decomposability constraint. As the problem is strictly convex, there is a unique solution. Thus, any non-uniqueness must come from our parameterization of Kronecker-sum-decomposable matrices. □

We now tie these two lemmas together to complete the statement of Theorem 2.

Theorem (Theorem 2)**.** *There exists a unique MLE (up to the non-identifiability of the diagonals of the Kronecker sum) that estimates the precision matrix of the singular Kronecker-sum-structured normal distribution, as long as* $r_\ell \leq \operatorname{rank}[\mathbf{S}_\ell]$*, subject to Assumption 4.*

Proof This follows directly from Lemmas 4 and 5. □

K Derivation of the Fisher information

This section contains the derivation of the Fisher Information, which is far too long and tedious to put in the main paper. For convenience, we repeat the definition of the NLL.

$$\mathrm{NLL} = \frac{-1}{2}\sum_{\gamma}\left(\log\det\left[\mathbf{t}^T\mathbf{a}^\gamma\mathbf{I}_{d^\gamma_\forall} + \bigoplus_{\ell\in\gamma}\mathbf{\Psi}_\ell\right] - \sum_{\ell\in\gamma}\mathrm{tr}\left[\mathbf{S}^\gamma_\ell\mathbf{\Psi}_\ell\right] - \sum_{\ell\in\gamma}\frac{\mathbf{t}^T\mathbf{a}^\gamma}{L^\gamma}\mathrm{tr}\left[\mathbf{S}^\gamma_\ell\right]\right)$$

Lemmas 6, 7, and 8 contain the calculations that derive the Hessian of the NLL.

Lemma 6. $\frac{\partial}{\partial\psi_{\ell_{ij}}}\mathrm{NLL} = -\frac{1}{2}\mathrm{sym}\left[\sum_{\gamma|\ell\in\gamma}\mathrm{tr}^{d^\gamma_{<\ell}}_{d^\gamma_{>\ell}}\left[\left(\mathbf{t}^T\mathbf{a}^\gamma\mathbf{I}_{\mathbf{d}^\gamma_\forall} + \bigoplus_{\ell\in\gamma}\mathbf{\Psi}_\ell\right)^{-1}\right]_{ij} - \mathbf{S}_{\ell_{ij}}\right]$

Proof This follows from Lemma 3. □

Lemma 7.

$$\frac{\partial}{\partial t_i}\mathrm{NLL} = \sum_\gamma -\frac{a^\gamma_i}{2}\mathrm{tr}\left[\left(\mathbf{t}^T\mathbf{a}^\gamma\mathbf{I}_{d^\gamma_\forall} + \bigoplus_{\ell\in\gamma}\mathbf{\Psi}_\ell\right)^{-1}\right] + \frac{a^\gamma_i}{2}\mathrm{tr}\left[\mathbf{S}^\gamma\right]$$

$$\frac{\partial}{\partial t_i\partial t_j}\mathrm{NLL} = \sum_\gamma \frac{a^\gamma_i a^\gamma_j}{2}\mathrm{tr}\left[\left(\mathbf{t}^T\mathbf{a}^\gamma\mathbf{I}_{d^\gamma_\forall} + \bigoplus_{\ell\in\gamma}\mathbf{\Psi}_\ell\right)^{-2}\right]$$

$$\frac{\partial}{\partial t_i\partial\psi_{\ell_{jk}}}\mathrm{NLL} = \mathrm{sym}\left[\sum_{\gamma|\ell\in\gamma}\frac{a^\gamma_i}{2}\mathrm{tr}^{d^\gamma_{<\ell}}_{d^\gamma_{>\ell}}\left[\left(\mathbf{t}^T\mathbf{a}^\gamma\mathbf{I}_{d^\gamma_\forall} + \bigoplus_{\ell\in\gamma}\mathbf{\Psi}_\ell\right)^{-2}\right]\right]_{jk}$$

Proof First, observe the following:

$$\begin{aligned}\frac{\partial}{\partial t_i}\log\det\left[\mathbf{t}^T\mathbf{a}^\gamma\mathbf{I}_{d^\gamma_\forall} + \bigoplus_{\ell\in\gamma}\mathbf{\Psi}_\ell\right] &= \mathrm{tr}\left[\left(\mathbf{t}^T\mathbf{a}^\gamma\mathbf{I}_{d^\gamma_\forall} + \bigoplus_{\ell\in\gamma}\mathbf{\Psi}_\ell\right)^{-1}\frac{\partial}{\partial t_i}\left(\mathbf{t}^T\mathbf{a}^\gamma\mathbf{I}_{d^\gamma_\forall} + \bigoplus_{\ell\in\gamma}\mathbf{\Psi}_\ell\right)\right] \\ &= \mathrm{tr}\left[\left(\mathbf{t}^T\mathbf{a}^\gamma\mathbf{I}_{d^\gamma_\forall} + \bigoplus_{\ell\in\gamma}\mathbf{\Psi}_\ell\right)^{-1}a^\gamma_i\mathbf{I}_{d^\gamma_\forall}\right] \\ &= a^\gamma_i\mathrm{tr}\left[\left(\mathbf{t}^T\mathbf{a}^\gamma\mathbf{I}_{d^\gamma_\forall} + \bigoplus_{\ell\in\gamma}\mathbf{\Psi}_\ell\right)^{-1}\right]\end{aligned}$$

Furthermore, note that:

$$\begin{aligned}\frac{\partial}{\partial t_i}\sum_{\ell\in\gamma}\frac{\mathbf{t}^T\mathbf{a}^\gamma}{L^\gamma}\mathrm{tr}\left[\mathbf{S}^\gamma_\ell\right] &= \sum_{\ell\in\gamma}\frac{a^\gamma_i}{L^\gamma}\mathrm{tr}\left[\mathbf{S}^\gamma_\ell\right] \\ &= \frac{a^\gamma_i}{L^\gamma}\mathrm{tr}\left[\mathbf{S}^\gamma\right]\end{aligned}$$

Putting these together yields the first claim:

$$\frac{\partial}{\partial t_i}\mathrm{NLL} = \sum_\gamma -\frac{a^\gamma_i}{2}\mathrm{tr}\left[\left(\mathbf{t}^T\mathbf{a}^\gamma\mathbf{I}_{d^\gamma_\forall} + \bigoplus_{\ell\in\gamma}\mathbf{\Psi}_\ell\right)^{-1}\right] + \frac{a^\gamma_i}{2}\mathrm{tr}\left[\mathbf{S}^\gamma\right]$$

Differentiating a second time results in the following:

$$\frac{\partial}{\partial t_i \partial t_j}\mathrm{NLL} = \sum_\gamma \frac{a_i^\gamma}{2}\mathrm{tr}\left[\left(\mathbf{t}^T\mathbf{a}^\gamma\mathbf{I}_{d_\forall^\gamma} + \bigoplus_{\ell\in\gamma}\mathbf{\Psi}_\ell\right)^{-1}\left(a_i^\gamma \mathbf{I}_{d_\forall^\gamma}\right)\left(\mathbf{t}^T\mathbf{a}^\gamma\mathbf{I}_{d_\forall^\gamma} + \bigoplus_{\ell\in\gamma}\mathbf{\Psi}_\ell\right)^{-1}\right]$$
$$= \sum_\gamma \frac{a_i^\gamma a_j^\gamma}{2}\mathrm{tr}\left[\left(\mathbf{t}^T\mathbf{a}^\gamma\mathbf{I}_{d_\forall^\gamma} + \bigoplus_{\ell\in\gamma}\mathbf{\Psi}_\ell\right)^{-2}\right]$$

Likewise, differentiating w.r.t. $\psi_{\ell_{jk}}$ gives us the final part of our result. We'll delay the symmetrization step until the end to prevent the equation running off the right margin of the page.

$$\frac{\partial^{\text{nosym}}}{\partial t_i \partial \psi_{\ell_{jk}}}\mathrm{NLL} = \sum_{\gamma|\ell\in\gamma} \frac{a_i^\gamma}{2}\mathrm{tr}\left[\left(\mathbf{t}^T\mathbf{a}^\gamma\mathbf{I}_{d_\forall^\gamma} + \bigoplus_{\ell\in\gamma}\mathbf{\Psi}_\ell\right)^{-1}\left(\mathbf{I}_{d_{<\ell}^\gamma}\otimes\mathbf{J}^{jk}\otimes\mathbf{I}_{d_{>\ell}^\gamma}\right)\left(\mathbf{t}^T\mathbf{a}^\gamma\mathbf{I}_{d_\forall^\gamma} + \bigoplus_{\ell\in\gamma}\mathbf{\Psi}_\ell\right)^{-1}\right]$$
$$= \sum_{\gamma|\ell\in\gamma} \frac{a_i^\gamma}{2}\mathrm{tr}_{d_{>\ell}^\gamma}^{d_{<\ell}^\gamma}\left[\left(\mathbf{t}^T\mathbf{a}^\gamma\mathbf{I}_{d_\forall^\gamma} + \bigoplus_{\ell\in\gamma}\mathbf{\Psi}_\ell\right)^{-2}\right]_{jk}$$
$$\frac{\partial}{\partial t_i \partial \psi_{\ell_{jk}}}\mathrm{NLL} = \mathrm{sym}\left[\sum_{\gamma|\ell\in\gamma} \frac{a_i^\gamma}{2}\mathrm{tr}_{d_{>\ell}^\gamma}^{d_{<\ell}^\gamma}\left[\left(\mathbf{t}^T\mathbf{a}^\gamma\mathbf{I}_{d_\forall^\gamma} + \bigoplus_{\ell\in\gamma}\mathbf{\Psi}_\ell\right)^{-2}\right]\right]_{jk}$$

This completes the proof. □

Lemma 8.

$$\frac{\partial^{\text{nosym}}}{\partial \psi_{\ell^1_{i^1j^1}}\partial \psi_{\ell^2_{i^2j^2}}}\mathrm{NLL} = \frac{1}{2}\sum_{\gamma|\ell^1,\ell^2\in\gamma}\mathrm{tr}_{d_{>\ell^1}^\gamma}^{d_{<\ell^1}^\gamma}\left[(\mathbf{\Omega}^\gamma)^{-1}\left(\mathbf{I}_{d_{<\ell^2}^\gamma}\otimes\mathbf{J}^{i^2j^2}\otimes\mathbf{I}_{d_{>\ell^2}^\gamma}\right)(\mathbf{\Omega}^\gamma)^{-1}\right]_{i^1j^1}$$

Proof This follows from Lemma 6. First we will compute the unsymmetrized derivative.

$$\frac{\partial^{\text{nosym}}}{\partial \psi_{\ell^1_{i^1j^1}}\partial \psi_{\ell^2_{i^2j^2}}}\mathrm{NLL} = -\frac{1}{2}\frac{\partial}{\partial \psi_{\ell^2_{i^2j^2}}}\sum_{\gamma|\ell^1\in\gamma}\mathrm{tr}_{d_{>\ell^1}^\gamma}^{d_{<\ell^1}^\gamma}\left[\left(\mathbf{t}^T\mathbf{a}^\gamma\mathbf{I}_{\mathbf{d}_\forall^\gamma} + \bigoplus_{\ell\in\gamma}\mathbf{\Psi}_{\ell^1}\right)^{-1}\right]_{i^1j^1}$$
$$= -\frac{1}{2}\frac{\partial}{\partial \psi_{\ell^2_{i^2j^2}}}\sum_{\gamma|\ell^1\in\gamma}\mathrm{tr}_{d_{>\ell^1}^\gamma}^{d_{<\ell^1}^\gamma}\left[(\mathbf{\Omega}^\gamma)^{-1}\right]_{i^1j^1}$$
$$= \frac{1}{2}\sum_{\gamma|\ell^1,\ell^2\in\gamma}\mathrm{tr}_{d_{>\ell^1}^\gamma}^{d_{<\ell^1}^\gamma}\left[(\mathbf{\Omega}^\gamma)^{-1}\left(\mathbf{I}_{d_{<\ell^2}^\gamma}\otimes\mathbf{J}^{i^2j^2}\otimes\mathbf{I}_{d_{>\ell^2}^\gamma}\right)(\mathbf{\Omega}^\gamma)^{-1}\right]_{i^1j^1}$$

If we let the unsymmetrized derivative be $\mathbf{X}_{\ell^1_{i^1j^1}\ell^2_{i^2j^2}}$, the symmetrized version is:

$$\frac{\mathbf{X}_{\ell^1_{i^1j^1}\ell^2_{i^2j^2}} + \mathbf{X}_{\ell^1_{j^1i^1}\ell^2_{i^2j^2}} + \mathbf{X}_{\ell^1_{i^1j^1}\ell^2_{j^2i^2}} + \mathbf{X}_{\ell^1_{j^1i^1}\ell^2_{j^2i^2}}}{4}$$

□

With these, we can derive **F**. As the derivation of inter-axis connections in **F** is complicated, and will be aided by the following lemma, which is just an application of the fact that the trace distributes over Kronecker products.

Lemma 9. *Let $\{\mathbf{M}_\ell\}_\ell$ be a subset of matrices where some subset X has the property that, for all $\ell \in X$, $\mathbf{M}_\ell = \mathbf{I}_{d_\ell\times d_\ell}$, where d_ℓ represents the size of the ℓth element, and, for all $\ell \notin X$, $\mathbf{M}_\ell = \mathbf{J}^{i_\ell j_\ell}_{d_\ell\times d_\ell}$ (the matrix of zeros except at with a 1 at position (i_ℓ, j_ℓ)).*

Then $\mathrm{tr}\left[\bigotimes_\ell \mathbf{M}_\ell\right] = \left(\prod_{\ell\in X} d_\ell\right)\left(\prod_{\ell\notin X}\delta_{i_\ell j_\ell}\right)$.

Proof

$$\begin{aligned}
\operatorname{tr}\left[\bigotimes_{\ell}\mathbf{M}_\ell\right] &= \prod_{\ell}\operatorname{tr}\left[\mathbf{M}_\ell\right] \\
&= \prod_{\ell\in X}\operatorname{tr}\left[\mathbf{M}_\ell\right]\prod_{\ell\notin X}\operatorname{tr}\left[\mathbf{M}_\ell\right] \\
&= \left(\prod_{\ell\in X} d_\ell\right)\prod_{\ell\notin X}\operatorname{tr}\left[\mathbf{M}_\ell\right] \\
&= \left(\prod_{\ell\in X} d_\ell\right)\left(\prod_{\ell\notin X}\delta_{i_\ell j_\ell}\right)
\end{aligned}$$

□

We are now equipped with all the tools we need to prove Lemma 10, the values of **F** under the null hypothesis.

Lemma 10. *Under the null hypothesis where* $\mathbf{\Omega}^\gamma = \frac{1}{(\sigma^\gamma)^2}\mathbf{I}_{d^\gamma_\forall}$, *we have the following:*

$$\begin{aligned}
\mathbf{F}_{t_i t_j} &= \sum_\gamma \frac{d^\gamma_\forall (\sigma^\gamma)^4}{2}\left(\mathbf{a}^\gamma\mathbf{a}^{\gamma,T}\right)_{ij} \\
\mathbf{F}_{t_i\psi_{\ell_{jk}}} &= \sum_{\gamma|\ell\in\gamma}\frac{a^\gamma_i d^\gamma_\forall (\sigma^\gamma)^4}{2d_\ell}\delta_{jk} \\
\mathbf{F}_{\psi_{\ell^1_{i^1j^1}}\psi_{\ell^2_{i^2j^2}}} &= \frac{1}{2}\sum_{\gamma|\ell^1,\ell^2\in\gamma}(\sigma^\gamma)^4\frac{d^\gamma_\forall}{d_{\ell^1}d_{\ell^2}}\delta_{i^2j^2}\delta_{i^1j^1} && (\ell^1\neq\ell^2) \\
\mathbf{F}_{\psi_{\ell^1_{i^1j^1}}\psi_{\ell^2_{i^2j^2}}} &= \frac{1}{2}\sum_{\gamma|\ell^1,\ell^2\in\gamma}(\sigma^\gamma)^4\frac{d^\gamma_\forall}{d_\ell}\left(\delta_{i^1j^1i^2j^2} + (1-\delta_{i^1j^1i^2j^2})\frac{\delta_{i^1i^2}\delta_{j^1j^2}}{2}\right) && (\ell^1=\ell^2)
\end{aligned}$$

Proof

$$\begin{aligned}
\frac{\partial}{\partial t_i\partial t_j}\text{NLL} &= \sum_\gamma\frac{a^\gamma_i a^\gamma_j}{2}\operatorname{tr}\left[\left(\mathbf{\Omega}^\gamma\right)^{-2}\right] \\
&= \sum_\gamma\frac{a^\gamma_i a^\gamma_j d^\gamma_\forall(\sigma^\gamma)^4}{2} \\
&= \sum_\gamma\frac{d^\gamma_\forall(\sigma^\gamma)^4}{2}\left(\mathbf{a}^\gamma\mathbf{a}^{\gamma,T}\right)_{ij} \\
\frac{\partial}{\partial t_i\partial\psi_{\ell_{jk}}}\text{NLL} &= \operatorname{sym}\left[\sum_\gamma\frac{a^\gamma_i}{2}\operatorname{tr}^{d^\gamma_{<\ell}}_{d^\gamma_{>\ell}}\left[\left(\mathbf{\Omega}^\gamma\right)^{-2}\right]_{jk}\right] \\
&= \operatorname{sym}\left[\sum_\gamma\frac{a^\gamma_i d^\gamma_\forall(\sigma^\gamma)^4}{2d_\ell}\left[\mathbf{I}_{d^\gamma_{\setminus\ell}}\right]_{jk}\right] \\
&= \frac{1}{2}\sum_\gamma\frac{a^\gamma_i d^\gamma_\forall(\sigma^\gamma)^4}{2d_\ell}\delta_{jk} + \frac{a^\gamma_i d^\gamma_\forall(\sigma^\gamma)^4}{2d_\ell}\delta_{kj} \\
&= \sum_\gamma\frac{a^\gamma_i d^\gamma_\forall(\sigma^\gamma)^4}{2d_\ell}\delta_{jk}
\end{aligned}$$

$$\begin{aligned}
\frac{\partial^{\text{nosym}}}{\partial\psi_{\ell^1_{i^1j^1}}\partial\psi_{\ell^2_{i^2j^2}}}\text{NLL} &= \frac{1}{2}\sum_{\gamma|\ell^1\in\gamma}\operatorname{tr}^{d^\gamma_{<\ell^1}}_{d^\gamma_{>\ell^1}}\left[\left(\mathbf{\Omega}^\gamma\right)^{-1}\left(\mathbf{I}_{d^\gamma_{<\ell^2}}\otimes\mathbf{J}^{i^2j^2}\otimes\mathbf{I}_{d^\gamma_{>\ell^2}}\right)\left(\mathbf{\Omega}^\gamma\right)^{-1}\right]_{i^1j^1} \\
&= \frac{1}{2}\sum_{\gamma|\ell^1\in\gamma}(\sigma^\gamma)^4\operatorname{tr}^{d^\gamma_{<\ell^1}}_{d^\gamma_{>\ell^1}}\left[\left(\mathbf{I}_{d^\gamma_{<\ell^2}}\otimes\mathbf{J}^{i^2j^2}\otimes\mathbf{I}_{d^\gamma_{>\ell^2}}\right)\right]_{i^1j^1}
\end{aligned}$$

Let us focus on what happens to the stridewise-blockwise trace term under algebraic manipulations.

$$\mathrm{tr}_{d^{\gamma}_{>\ell^1}}^{d^{\gamma}_{<\ell^1}}\left[\left(\mathbf{I}_{d^{\gamma}_{<\ell^2}}\otimes\mathbf{J}^{i^2j^2}+\mathbf{J}^{j^2i^2}\otimes\mathbf{I}_{d^{\gamma}_{>\ell^2}}\right)\right]_{i^1j^1}=\mathrm{tr}\left[\left(\mathbf{I}_{d^{\gamma}_{<\ell^2}}\otimes\mathbf{J}^{i^2j^2}\otimes\mathbf{I}_{d^{\gamma}_{>\ell^2}}\right)\left(\mathbf{I}_{d^{\gamma}_{<\ell^1}}\otimes\mathbf{J}^{i^1j^1}\otimes\mathbf{I}_{d^{\gamma}_{>\ell^1}}\right)\right]$$

We now split the $(\psi_{\ell^1_{i^1j^1}},\psi_{\ell^2_{i^2j^2}})$ computation into two cases, one in which $\ell=\ell^1=\ell^2$ and another where $\ell^1\neq\ell^2$. In both cases we use Lemma 9.

Case 1: $\ell^1\neq\ell^2$

$$\mathrm{tr}_{d^{\gamma}_{>\ell^1}}^{d^{\gamma}_{<\ell^1}}\left[\left(\mathbf{I}_{d^{\gamma}_{<\ell^2}}\otimes\mathbf{J}^{i^2j^2}\otimes\mathbf{I}_{d^{\gamma}_{>\ell^2}}\right)\right]_{i^1j^1}=\frac{d^{\gamma}_{\forall}}{d_{\ell^1}d_{\ell^2}}\delta_{i^2j^2}\delta_{i^1j^1}$$

Case 2: $\ell=\ell^1=\ell^2$

$$\begin{aligned}\mathrm{tr}_{d^{\gamma}_{>\ell}}^{d^{\gamma}_{<\ell}}\left[\left(\mathbf{I}_{d^{\gamma}_{<\ell}}\otimes\mathbf{J}^{i^2j^2}\otimes\mathbf{I}_{d^{\gamma}_{>\ell}}\right)\right]_{i^1j^1}&=\mathrm{tr}\left[\left(\mathbf{I}_{d^{\gamma}_{<\ell}}\otimes\mathbf{J}^{i^2j^2}\otimes\mathbf{I}_{d^{\gamma}_{>\ell}}\right)\left(\mathbf{I}_{d^{\gamma}_{<\ell}}\otimes\mathbf{J}^{i^1j^1}\otimes\mathbf{I}_{d^{\gamma}_{>\ell}}\right)\right]\\&=\mathrm{tr}\left[\left(\mathbf{I}_{d^{\gamma}_{<\ell}}\otimes\mathbf{J}^{i^2j^2}\mathbf{J}^{i^1j^1}\otimes\mathbf{I}_{d^{\gamma}_{>\ell}}\right)\right]\\&=\frac{d^{\gamma}_{\forall}}{d_{\ell}}\left(\mathrm{tr}\left[\mathbf{j}^{i^2}\mathbf{j}^{j^2,T}\mathbf{j}^{i^1}\mathbf{j}^{j^1,T}\right]\right)\\&=\frac{d^{\gamma}_{\forall}}{d_{\ell}}\left(\mathrm{tr}\left[\mathbf{j}^{j^2,T}\mathbf{j}^{i^1}\mathbf{j}^{j^1,T}\mathbf{j}^{i^2}\right]\right)\\&=\frac{d^{\gamma}_{\forall}}{d_{\ell}}\left(\delta_{j^2i^1}\delta_{j^1i^2}\right)\end{aligned}$$

This is the nonsymmetric derivative. Symmetricizing requires replacing $\delta_{j^2i^1}\delta_{j^1i^2}$ with the average over all symmetries, swapping i^1 and j^1 and swapping i^2 and j^2. These symmetries do not affect the different axis case, but do affect the same-axis case.

One of the symmetries of $\delta_{j^2i^1}\delta_{j^1i^2}$ is $\delta_{i^1i^2}\delta_{j^1j^2}$, which we will rewrite as $\delta_{(i^1,j^1)(i^2,j^2)}$ to make it more clear what is being compared.

Suppose $i^1=j^1$ is diagonal. Then this is only true for $i^1=i^2=j^1=j^2$, which if true under one symmetry then it is true under all symmetries.

Suppose now that neither are diagonal, and recall we required $i\leq j$. Then, any symmetries that swap the ordering of i^1 and j^1 will never be satisfied, unless they also swap i^2 and j^2. This lets us rewrite the same-axis case as:

$$\delta_{i^1j^1i^2j^2}+(1-\delta_{i^1j^1i^2j^2})\frac{\delta_{i^1i^2}\delta_{j^1j^2}}{2}$$

This completes the proof.

□

Recall that the only independent parameters are $\mathbf{t}$ and $\psi_{\ell_{ij}}$ for $i\leq j$ and $(i,j)\neq(1,1)$. There are three types of parameters here; $\mathbf{t}$, diagonal $\psi_{\ell_{ii}}$, and off-diagonal $\psi_{\ell_{ij}}$. Of particular interest to us are the interactions between off-diagonal elements $\psi_{\ell_{ij}}$ (where $\delta_{ij}=0$). It is clear they cannot interact with $\mathbf{t}$ terms due to the δ_{ij} term in its formula. They also cannot interact with any $\psi_{\ell'_{i'j'}}$ in different axes, since the $\delta_{ij}\delta_{i'j'}$ term is only nonzero for the diagonal elements. Finally, the δ terms governing interactions within the same axis are only nonzero when $(i,j)=(i',j')$ (self-interactions) or $(i,j)=(j',i')$, which for off-diagonal elements can never be true due to the $i\leq j$ requirement.

	$\mathbf{t}$	Diagonal ψ_{ii}	Off-diagonal ψ_{ij}
$\mathbf{t}$	Yes[a]	Yes	No
Diagonal ψ_{ii}	Yes	Only in different axes	No
Off-diagonal ψ_{ij}	No	No	No

Table S2. Allowed interactions between parameters in **F**.

[a] This depends on $\{\mathbf{a}^{\gamma}\}$. For most datasets, the most natural choice of identifiable parameterization results in the **t**-**t** interactions forming a diagonal matrix (none of the **t** interact).

For all possible interactions, consult Table S2 - these can be derived by investigating the δ terms in the formula for **F**. The fact that off-diagonal elements do not interact with anything in **F** is quite significant, as seen by the following corollary to Lemma 10.

Corollary 1. *Suppose we order* $\mathbf{F}$ *such that the first rows/columns represent* $\mathbf{t}$ *terms, the next represent diagonal* ψ_{ii} *terms, and the last represent the off-diagonal terms. Then, we have the following:*

$$\mathbf{F} = \begin{bmatrix} \mathbf{A} & \mathbf{0} \\ \mathbf{0} & \mathbf{B} \end{bmatrix}$$

$\mathbf{B}$ *represents the off-diagonal terms, and* ***is a diagonal matrix****. This allows a convenient expression for* $\mathbf{F}^{-1}$*, by inverting blockwise.*

We will now give the example of a dataset $\gamma_1 = (\ell_1, \ell_2), \gamma_2 = (\ell_1, \ell_3)$ where $d_{\ell_1} = 3$ and $d_{\ell_2} = d_{\ell_3} = 2$. Note that $\mathbf{a}^{\gamma_i} = \begin{bmatrix} \delta_{1i} & \delta_{2i} \end{bmatrix}$ in this case (and most cases). This leads to $\mathbf{F}_{\mathbf{t},\mathbf{t}}$ being proportional to the identity matrix. If we let $A^\gamma = \frac{d_\forall^\gamma (\sigma^\gamma)^4}{2}$, then it becomes easy to express the matrix. Exact derivations are left as an exercise to the reader.

$$\begin{array}{c} \\ \\ \sum_\gamma A^\gamma \end{array}
\begin{array}{ccccccccccc|c}
t_1 & t_2 & \ell_1\,(2,2) & \ell_1\,(3,3) & \ell_2\,(2,2) & \ell_3\,(2,2) & \ell_1\,(1,2) & \ell_1\,(1,3) & \ell_1\,(2,3) & \ell_2\,(1,2) & \ell_3\,(1,2) & \\
\hline
1 & 0 & \frac{1}{d_{\ell_1}} & \frac{1}{d_{\ell_1}} & \frac{1}{d_{\ell_2}} & \frac{1}{d_{\ell_3}} & 0 & 0 & 0 & 0 & 0 & t_1 \\
0 & 1 & \frac{1}{d_{\ell_1}} & \frac{1}{d_{\ell_1}} & \frac{1}{d_{\ell_2}} & \frac{1}{d_{\ell_3}} & 0 & 0 & 0 & 0 & 0 & t_2 \\
\frac{1}{d_{\ell_1}} & \frac{1}{d_{\ell_1}} & \frac{1}{d_{\ell_1}} & 0 & \frac{1}{d_{\ell_1 \ell_2}} & \frac{1}{d_{\ell_1 \ell_3}} & 0 & 0 & 0 & 0 & 0 & \ell_1\,(2,2) \\
\frac{1}{d_{\ell_1}} & \frac{1}{d_{\ell_1}} & 0 & \frac{1}{d_{\ell_1}} & \frac{1}{d_{\ell_1 \ell_2}} & \frac{1}{d_{\ell_1 \ell_3}} & 0 & 0 & 0 & 0 & 0 & \ell_1\,(3,3) \\
\frac{1}{d_{\ell_2}} & \frac{1}{d_{\ell_2}} & \frac{1}{d_{\ell_1} d_{\ell_2}} & \frac{1}{d_{\ell_1} d_{\ell_2}} & \frac{1}{d_{\ell_2}} & \frac{1}{d_{\ell_2 \ell_3}} & 0 & 0 & 0 & 0 & 0 & \ell_2\,(2,2) \\
\frac{1}{d_{\ell_3}} & \frac{1}{d_{\ell_3}} & \frac{1}{d_{\ell_1} d_{\ell_3}} & \frac{1}{d_{\ell_1} d_{\ell_3}} & \frac{1}{d_{\ell_2} d_{\ell_3}} & \frac{1}{d_{\ell_3}} & 0 & 0 & 0 & 0 & 0 & \ell_3\,(2,2) \\
0 & 0 & 0 & 0 & 0 & 0 & \frac{1}{2d_{\ell_1}} & 0 & 0 & 0 & 0 & \ell_1\,(1,2) \\
0 & 0 & 0 & 0 & 0 & 0 & 0 & \frac{1}{2d_{\ell_1}} & 0 & 0 & 0 & \ell_1\,(1,3) \\
0 & 0 & 0 & 0 & 0 & 0 & 0 & 0 & \frac{1}{2d_{\ell_1}} & 0 & 0 & \ell_1\,(2,3) \\
0 & 0 & 0 & 0 & 0 & 0 & 0 & 0 & 0 & \frac{1}{2d_{\ell_2}} & 0 & \ell_2\,(1,2) \\
0 & 0 & 0 & 0 & 0 & 0 & 0 & 0 & 0 & 0 & \frac{1}{2d_{\ell_3}} & \ell_3\,(1,2)
\end{array}$$

With everything in place, we can now define the hypothesis test.

Theorem (Theorem 3)**.** *Under the null hypothesis* $\boldsymbol{\Omega}^\gamma = \frac{1}{(\sigma^\gamma)^2} \mathbf{I}_{d_\forall^\gamma}$*, we have the following distribution for each* $\psi_{\ell_{ij}}$ *independently:*

$$\sqrt{\frac{\sum_{\gamma | \ell \in \gamma} d_\forall^\gamma (\sigma^\gamma)^4}{d_\ell}} \frac{\psi_{\ell_{ij}}}{2} \dot{\sim} \mathcal{N}(0, 1)$$

Proof As noted in Corollary 1, the edge-edge interactions in $\mathbf{F}$ form a diagonal matrix, and there are no cross-terms connecting edges to diagonal and $\mathbf{t}$ parameters. Thus, inversion is simple.

By Lemma 10, each edge-edge precision term for axis ℓ in $\mathbf{F}$ can be expressed as $\sum_{\gamma | \ell \in \gamma} \frac{d_\forall^\gamma (\sigma^\gamma)^4}{4 d_\ell}$. In $\mathbf{F}^{-1}$, this becomes $\frac{4 d_\ell}{\sum_{\gamma | \ell \in \gamma} d_\forall^\gamma (\sigma^\gamma)^4}$.

Thus, under the null hypothesis, we have that:

$$\psi_{\ell_{ij}} \dot{\sim} \mathcal{N}\left(0, \frac{4 d_\ell}{\sum_{\gamma | \ell \in \gamma} d_\forall^\gamma (\sigma^\gamma)^4}\right)$$

$$\sqrt{\frac{\sum_{\gamma | \ell \in \gamma} d_\forall^\gamma (\sigma^\gamma)^4}{d_\ell}} \frac{\psi_{\ell_{ij}}}{2} \dot{\sim} \mathcal{N}(0, 1)$$

□

This completes the derivations. While deriving such a test was tedious, it gives us an interpretable way to set the threshold for our recovered graphs, by choosing to only keep statistically significant edges!

L Preserving input sparsity

The final practical issue to surmount is that of preserving input sparsity. $\text{mat}_\ell\left[\{\mathcal{D}^\gamma\}_\gamma\right]$ has $d_\forall$ elements, but in many contexts, such as transcriptomics, the dataset will be highly sparse; the majority of the input is full of zeros. Ideally, we would never 'densify' the matrix. Using COCA, however, requires computing the matrix of ranks, which will be fully dense. If unaddressed, this would enforce an $\Omega(d_\forall)$ minimum memory requirement. We will demonstrate the problem with an example, where Φ represents the cumulative density function of the normal distribution.

Example 8 (Demonstration that ranking densifies data)**.**

$$\begin{bmatrix} 3 & 0 & -2 & 0 & 4 \\ 0 & -1 & -3 & 0 & -2 \end{bmatrix} \to \begin{bmatrix} 4 & 2 & 1 & 2 & 5 \\ 4 & 3 & 1 & 4 & 2 \end{bmatrix} \qquad \text{(rank data)}$$

$$\to \begin{bmatrix} \Phi^{-1}\left(\frac{4}{6}\right) & \Phi^{-1}\left(\frac{2}{6}\right) & \Phi^{-1}\left(\frac{1}{6}\right) & \Phi^{-1}\left(\frac{2}{6}\right) & \Phi^{-1}\left(\frac{5}{6}\right) \\ \Phi^{-1}\left(\frac{4}{6}\right) & \Phi^{-1}\left(\frac{3}{6}\right) & \Phi^{-1}\left(\frac{1}{6}\right) & \Phi^{-1}\left(\frac{4}{6}\right) & \Phi^{-1}\left(\frac{2}{6}\right) \end{bmatrix} \qquad \text{(map to normal)}$$

$$\approx \begin{bmatrix} 0.43 & -0.43 & -0.97 & -0.43 & 0.97 \\ 0.43 & 0 & -0.97 & 0.43 & -0.43 \end{bmatrix}$$

The exact values depend on how you handle ties when ranking data, but the general idea remains the same regardless. The trick to avoiding the densification is to realize that, in each row, all zero values get mapped to the same final value. We can rewrite our example as:

$$\begin{bmatrix} 0.43 + 0.43 & 0 & -0.97 + 0.43 & 0 & 0.97 + 0.43 \\ 0 & -0.43 & -0.97 - 0.43 & 0 & -0.43 - 0.43 \end{bmatrix} + \begin{bmatrix} -0.43 \\ 0.43 \end{bmatrix} \mathbf{1}_{1\times 5}$$

Let z_i be the normally-distributed value that the zeros in row i get mapped to. We can express our transformation more generally:

$$\begin{bmatrix} \mathbf{x}_1 \\ \dots \\ \mathbf{x}_a \end{bmatrix} = \begin{bmatrix} \mathbf{x}_1 - z_1 \\ \dots \\ \mathbf{x}_a - z_a \end{bmatrix} + \mathbf{z}\mathbf{1}^T$$

Crucially, we have expressed our transformed data as the sum of a matrix with the same sparsity structure as our original data, and a rank-one matrix. If we compute the SVD on the first matrix, we can then use a rank-one update algorithm to calculate the SVD of the sum of the two matrices [Brand, 2006]. It is not difficult to work out $\mathbf{z}$, and hence we can perform this procedure without ever densifying our input matrix.

M Implementation

The algorithm is implemented in Python, using Numpy, Numba, SciPy, and Dask [Harris et al., 2020, Lam et al., 2015, Virtanen et al., 2020, Rocklin, 2015]. The experiments were run on a 13-inch MacBook Pro with an M1 chip and 8GB RAM. 23GB of hard disk storage were available when running the experiments; the million cell experiment (Section 3.1) could not fit in 8GB of RAM, and thus much of the free hard disk space was used as swap memory. The algorithm itself is given in Appendix B. Table 1 describes the expected memory requirements of the algorithm across a variety of sizes.

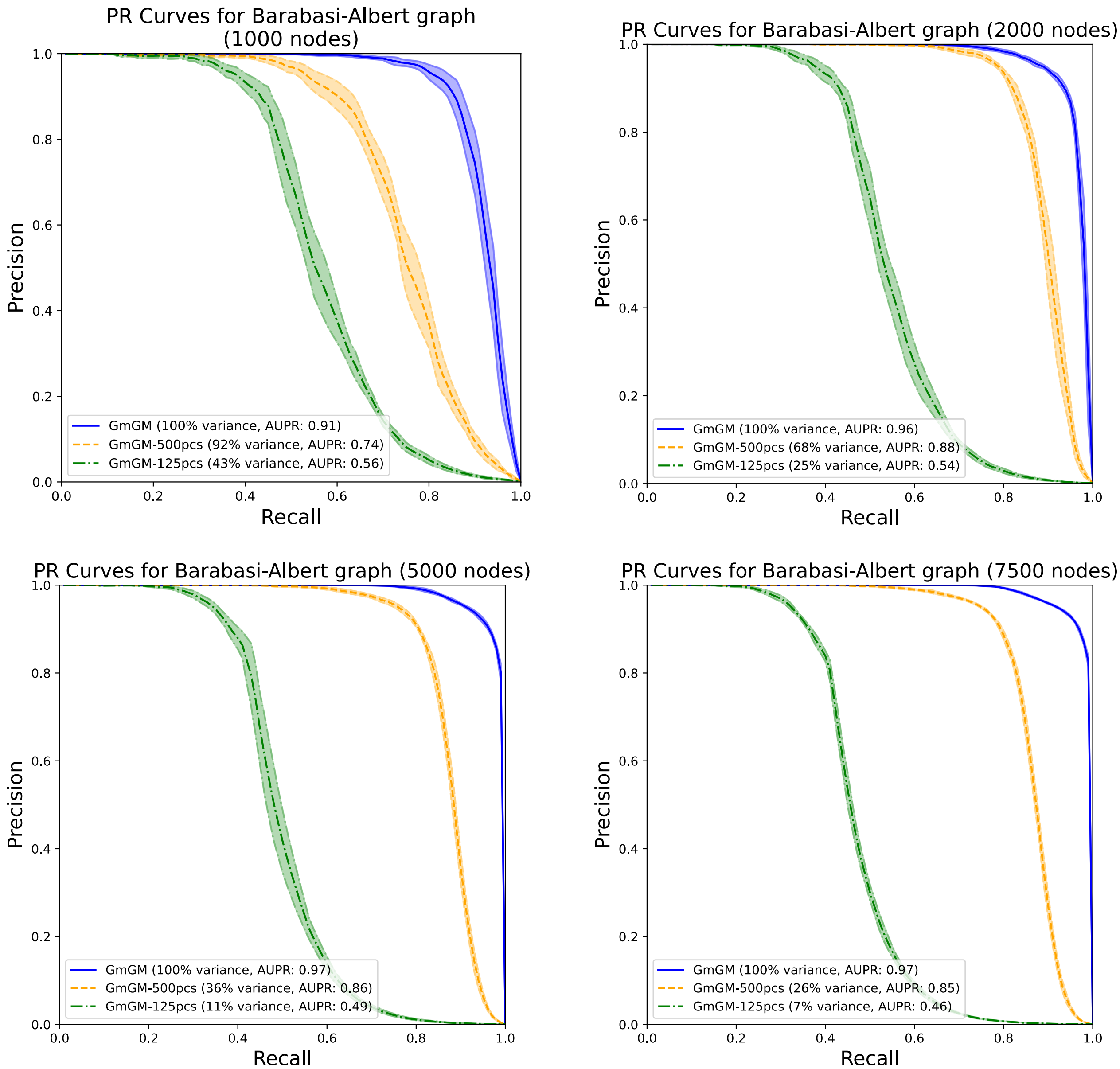

Fig. S3. Precision-recall curves, with AUPR reported, for $n \in \{1000, 2000, 5000, 7500\}$ datasets of shape $n \times n$ whose graphs from from a Barabasi-Albert distribution. We compare the full-rank GmGM algorithm, along with our proposed algorithm with 500 principal components and a more extreme 125 principal components. We see that 500 principal components still yield good results as the dataset size increases.

N Additional experiments

N.1 Synthetic data

In this section and in the main paper, we generated ground truth graphs from the Barabasi-Albert distribution. We chose this distribution because it is a scale-free distribution; many real-world networks, such as social networks and gene regulatory networks, are approximately scale-free [Barabási and Albert, 1999]. We then generated synthetic data using these graphs and the Kronecker-sum-structured normal distribution.

To test performance, and area-under-PR curves (AUPR) for GmGM and our proposed modifications to GmGM; when an algorithm correctly identifies an edge, it is considered a true positive. When comparing to prior work, we used their default convergence tolerance and max iterations.

We report our PR curves and their AUPRs in Figure S3. It is clear that, for a fixed amount of components, the AUPR stays roughly the same as the axis size increases. This is true even as the percentage of variance explained decreases. There is a clear trade-off between the performance of the method and the number of principal components chosen. Keeping 500 components still

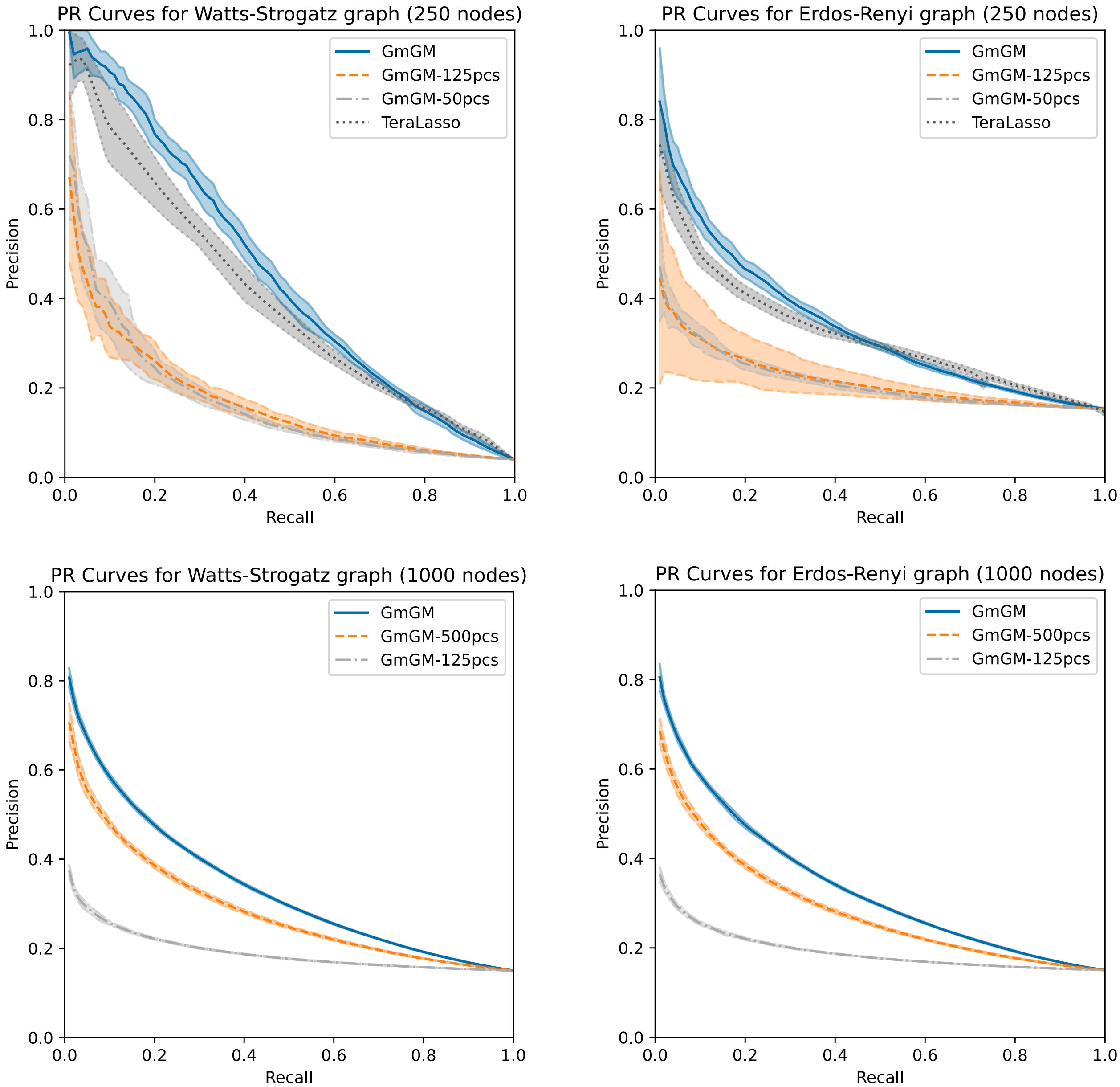

Fig. S4. Precision-recall curves for 250×250 (top) and 1000×1000 (bottom) datasets generated from either the Watts-Strogatz model (left, via Python iGraph: `ig.Graph.Watts_Strogatz(1, numnodes, 75, 0.8)`) or the Erdos-Renyi model (right, `ig.Graph.Erdos_Renyi(n=numnodes, p=0.15)`). TeraLasso is compared against for the smaller networks to show that poorer performance on these networks is not specific to GmGM.

has high performance even as the percent of variance explained decreases; this shows the method is suitable on problem sizes that full-rank methods such as GmGM simply cannot handle.

We also briefly investigate the performance of our model under different generative models (Figure S4). Neither of these models generate power-law networks; Erdos-Renyi graphs have each edge being fully independent and identically distributed, whereas Watts-Strogatz graphs allow for clusters to form. The performance of our method is poorer on these, but importantly this is true **even when one does not use the low-rank approximation**, and also **true for TeraLasso**.

N.2 COIL Dataset

It is difficult to test on real-world data, as ground truth graphs are often unknown. This is perhaps why the COIL-20 video dataset [Nene et al., 1996] has become a standard test for multi-axis models.

The dataset consists of 20 videos of 72 frames and 128x128 pixels. Each video consists of a simple object rotating 360 degrees, and thus the frame graph can reasonably expected to correspond to this circular structure. To test this, we considered the dataset

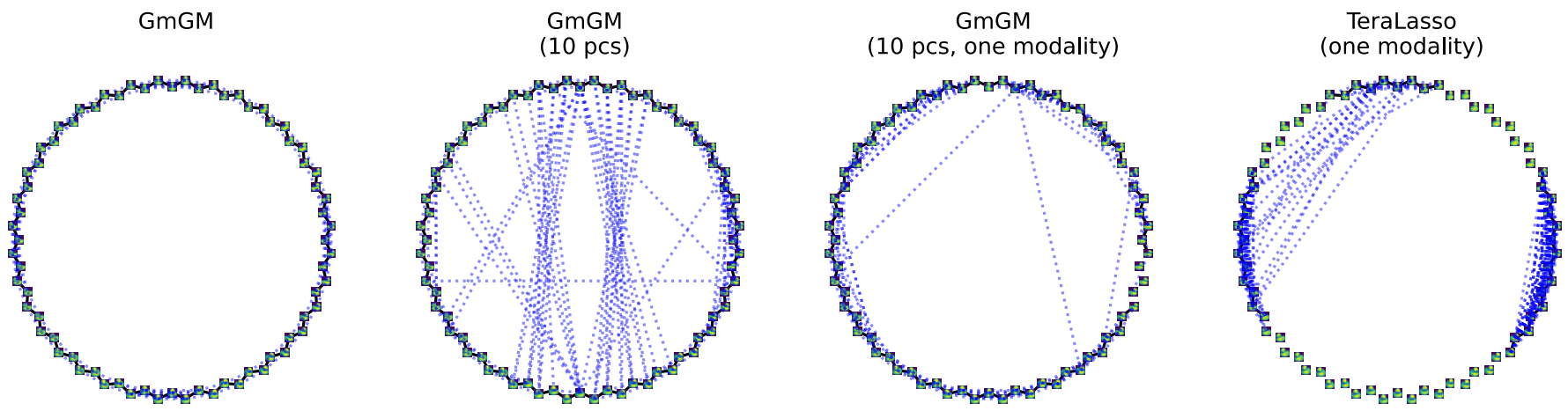

Fig. S5. A comparison of several methods to find the frame graph.

Connections	Adjacent	One off	Two off	Three off	More	Total	Method
GmGM	72	72	25	4	0	173	p=0.05
GmGM (10pcs)	72	64	13	0	51	200	p=0.05
GmGM (10pcs, one modality)	66	58	44	13	19	200	Thresholding
TeraLasso (one modality)	33	31	26	22	85	197[a]	Thresholding

[a] We were not able to get exactly 200 edges

Table S3. The performance of several methods on reconstructing the frame graph of COIL-20 videos. The adjacent column denotes how many edges were between adjacent frames (the 'correct' edges), whereas the later columns denote how many edges exist between frames i and $i \pm (n+1)$ for various n. The More column summarizes all connections where $n > 3$.

Connections	Precision/Recall (exact)	Precision/Recall (one off)	Precision/Recall (two off)	Precision/Recall (three off)
GmGM	**41.6%/100%**	**83.2%/100%**	**97.6%/78.2%**	**100%**/60%
GmGM (10pcs)	36%/**100%**	60%/94.4%	74.5%/69%	74.5%/51.7%
GmGM (10pcs, one modality)	33%/91.7%	62%/86%	84%/77.8%	90.5%/**62.8%**
TeraLasso (one modality)	16.8%/45.8%	32.5%/44.4%	45.7%/41.7%	56.7%/38.9%

Table S4. The precisions and recalls for each algorithm, when we consider correct edges to be those between edges i and $i \pm (n+1)$, for $n = 0$ ('exact'), $n = 1$ ('one off'), $n = 2$ ('two off'), and $n = 3$ ('three off'). In each column, the best performing model's results were given in bold for each metric. **Bold numbers** represent the best performance overall, and blue numbers represent the best performance when restricted to one modality.

to have the form $\left\{\gamma_i = (\ell_{\text{frame}}, \ell_{\text{row}(i)}, \ell_{\text{col}(i)})\right\}_i$, that is, that there is a singular frame graph describing the rotational structure of the images, and then individual row and column graphs for each object (since the structure of each object may be different). In other words, the dataset had 20 modalities, each of size 72x128x128. TeraLasso can only handle a single modality, so to compare against it we ran our algorithm another time, this time on only one modality (the duck object, "obj1"). We compared the results using all principal components and when using only the top 10.

In the aforementioned experiments, we kept only the edges that were statistically significant to the 5% level after applying the Bonferroni correction. No edges were statistically significant, so instead we kept the same amount of edges as were found to be significant when considering the whole dataset (200). We then did the same with TeraLasso.

The results can be compared visually in Figure S5. These show that GmGM over all modalities does the best, which is to be expected - it uses the most information and does not make a low-rank assumption. TeraLasso cannot handle data of this form; however, even if we restrict our data to only a single modality and 10 principal components, our method performs best. Numerical values are given in Tables S3 and S4; we can see that, when considering statistically significant edges in the whole dataset, our method finds all the correct edges (even with only 10 principal components). The false positives it finds are typically close-to-correct, which is reasonable.

N.3 Sanity check of million-cell results

At the beginning of this paper, we promised scalability to a million cells. Here, we will use a 1,248,980 cell, 36,571 gene PBMC dataset by Yazar et al. [2022]. For preprocessing, we removed any cells and genes that had 0 counts everywhere. Then, we kept

Algorithm	Cluster	Biological Process	p-value
GmGM (p=0.05)	1 (67 genes)	cell-cell adhesion	1.0×10^{-9}
		apoptotic process	3.1×10^{-9}
		positive regulation of cellular process	5.1×10^{-9}
	2 (65 genes)	protein maturation	5.6×10^{-7}
		response to endoplasmic reticulum stress	1.3×10^{-6}
		organonitrogen compound metabolic process	9.1×10^{-6}
	3 (60 genes)	immune response	9.5×10^{-15}
		leukocyte mediated immunity	3.8×10^{-14}
		immune system process	8.7×10^{-14}
	4 (43 genes)	GO:0048523	4.7×10^{-5}
		GO:0051172	6.8×10^{-5}
		GO:0031324	8.9×10^{-5}
	5 (27 genes)	positive regulation of immune system process	8.3×10^{-15}
		regulation of immune response	2.3×10^{-14}
		GO:0002504	2.4×10^{-14}
	6 (14 genes)	intracellular sequestering of iron ion	8.1×10^{-3}
		sequestering of iron ion	1.1×10^{-2}
		negative regulation of actin filament polymerization	1.3×10^{-2}

Table S5. GO terms with long names have been replaced with their GO ID; most were of the form "negative regulation of cellular/nitrogen compound (metabolic) process". Immune-related GO terms have been colored blue. These were identified using the Python API of the GProfiler [Kolberg et al., 2023] functional enrichment tool. All clusters are represented in this table; the remaining genes were all singletons in the GmGM network. The reason there are so few clusters compared to the lncRNA experiment is because we kept only the statistically significant edges, rather than the top 5 edges per vertex as in the lncRNA experiment.

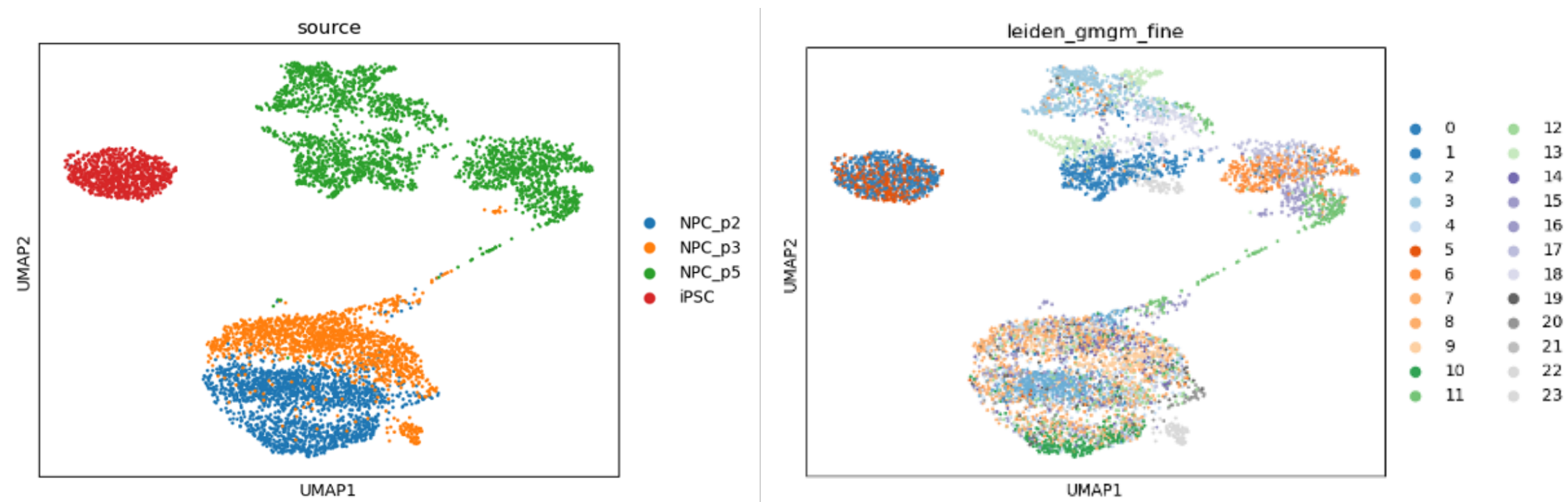

Fig. S6. (Left) The four timepoints of the data, displayed as a UMAP plot. The timpoints are labeled (in order): 'iPSC', 'NPC_p2', 'NPC_p3', and 'NPC_p5'. (Right) The cell clusters after applying Leiden to GmGM's output graph. Each of these clusters is then assigned a cell type, yielding the assignment shown in Figure S7.

only the top 2,200 highly variable genes. No cells had uniformly 0 counts, so the post-processing dataset size contained 1,249,980 cells and 2200 genes.

Due to the size of the dataset, we kept only the top 50 principal components (∼50% of the explained variance). We keep only the statistically significant edges and use the Nonparanormal Skeptic. Overall, 5890 edges were statistically significant. We do not compare to prior work as this dataset is too large for them.

We then investigated the GO terms of the clusters of our graphs. If our graph were nonsense, we would expect there to be no significant GO terms associated with any clusters. However, in Table S5 we can see that this is not the case; we find six clusters in total, each corresponding to meaningful groupings of genes. These include immune-related groupings (which we would expect and hope to find in a PBMC dataset, which contain mostly immune cells). There were no other clusters; all remaining genes had no statistically significant edges and hence were singletons.

This example ran in only a couple of minutes - faster than would be predicted from the runtimes in Figure 2, likely due to the high sparsity of the dataset[5].

O Neuronal scRNA-seq dataset details

The dataset consists of four separate timepoints; the original iPSC cells, as well as cells taken from passages 2, 3, and 5 ('p2', 'p3', and 'p5'); these are shown graphically in Figure S6. In total, there are 6876 cells and 29,324 genes. The dataset was generated in-house; the following subsections describe how this was done.

[5] Figure 2 gives performance on dense datasets, whereas our algorithm, unlike prior work, benefits substantially from sparsity in the dataset as well. This is because there are many efficient partial eigendecomposition routines for sparse data.

Cell type	Marker genes
induced pluripotent stem cells	NANOG, POU5F1, LIN28A, DPPA4, TDGF1, ZSCAN10, UTF1
neural progenitors	PAX6, HES1, NES1, MSI1, VIM, FABP7
cortical neurons	TBR1, BCL11B, MAP2, RBFOX3, SLC17A7, SYT1, GRIN2B
astrocytes	NFIA, SOX9, GFAP, AQP4, S100B, ALDH1L1, GLUL, SLC1A3, SLC1A2
oligodendrocytes	SOX10, MYRF, MBP, MOG, PLP1, MAG, CNP
neural ectoderm	SOX1, ZIC1, OTX2, LHX2, EMX2, DACH1, SIX3

Table S6. Marker genes for various neuronal cell types.

O.1 Culture of induced pluripotent stem cells (iPSCs)

STEMCELL Technologies's SCTi003-A line (STEMCELL Technologies; #200-0511) were cultured in complete mTeSR plus media (STEMCELL Technologies) on Matrigel® coated (Corning) 6-well plates (Helena Biosciences) and passaged at 1:40-1:60 split ratio with ReLeSR (STEMCELL Technologies) at approximately 70% confluency. Cultures were regularly tested for pluripotency by immunocytochemistry and mycoplasma using MycoStrip lateral flow tests (InvivoGen).

O.2 iPSC-derived neural progenitor cell (NPC) differentiation

Neural induction of iPSCs was performed using the STEMdiff SMADi Neural Induction Kit (STEMCELL Technologies) according to the manufacturer's monolayer protocol. iPSCs were dissociated with Accutase (Sigma) and resuspended as a single-cell suspension in DMEM/F12 (Thermo Fisher Scientific) for counting. 2.5×10^6 cells were pelleted at 300g for 5 minutes and resuspended in Neural Induction + SMADi Medium (NIM; STEMCELL Technologies) supplemented with 10 µM Y-27632 onto hESC-qualified Matrigel®-coated 6-well plates. Medium was replaced daily for 6–9 days, after which cells were passaged using Accutase and re-plated at 2×10^6 cells per well. This induction–passage cycle was repeated until passage 3, at which point cells were re-plated at 1.25×10^6 cells per well and subsequently maintained in STEMdiff Neural Progenitor Medium (STEMCELL Technologies) for all following passages.

O.3 Cell fixation

iPSCs were dissociated with Accutase at low passage and approximately 70% confluency prior to fixation. NPCs were similarly lifted with Accutase on their designated passage day; for example, p2 NPCs were collected at passage 2 of differentiation. This procedure was performed for p2, p3, and p5 NPCs. Following cell counting, 1×10^6 to 2×10^6 cells were fixed using the Evercode Cell Fixation v2 kit (Parse Biosciences) according to the manufacturer's user manual (v2.1.1). Final post-fixation yields ranged from 7×10^5 to 1×10^6 cells, which were subsequently carried forward to barcoding.

O.4 Barcoding, cDNA library preparation and sequencing

Approximately 7×10^4 per sample were processed for barcoding and library preparation using the Evercode WT kit (v2.2.1) according to the manufacturers protocol (Parse Biosciences). Nine libraries were generated and assessed for fragment size and quality using an Agilent TapeStation prior to sequencing. The libraries were subsequently pooled and sequenced across two P4 flow cells on an Illumina NextSeq2000 using a 2x200bp paired end cycle. The resulting fastq files were processed using Trailmaker (Parse Biosciences) to generate gene read-counts per cell per timepoint. This count matrix is what was passed into GmGM.

P Neuronal dataset cell marker genes

We used the marker genes given in Table S6 to score each cluster. A cell cluster was considered statistically significantly associated with a gene if the gene expression was significantly different in that cluster compared to others; cell clusters were then assigned the cell type corresponding to the one whose marker genes were the most commonly significantly differentially expressed.

Q GO terms for network metrics

In Figure 3, we picked which GO terms in two groups - those which are important processes dominant in all cells, and those which are part of the development of a key component found in neurons. For each GO term category (such as 'DNA replication' or 'axon development'), we selected the GO term most relevant to the process, all GO terms that are annotated as *regulates*, *positively_regulates*, *negatively_regulates*, or *occurs_in* the original term, and then any children and descendents of those GO terms via *part_of* or *is_a* relations. The precise GO terms are given in Table S7.

R Neuronal scRNA-seq: comparison against a simple baseline

In Section 3, we compared our work against hdWGCNA and kNN; hdWGCNA is a state-of-the-art methodology and kNN is the default cell network methodology in major workflows such as scanpy [Wolf et al., 2018], and thus comparing against them allows practicing bioinformaticians to understand the merits of our methodology relative to what they are already using. However, there are many factors differentiating GmGM and hdWGCNA/kNN, not just the multi-axis nature of our algorithm. The equivalent single-axis estimator to GmGM is the maximum likelihood estimator of the precision matrix, which under a low rank assumption is

Reason	Category	Fundamental GO terms	Regulators/Occurs-In
Fundamental Process	DNA Replication	GO:0006260	GO:0008156, GO:0045740, GO:0006275
Fundamental Process	Cell Cycle	GO:0007049	GO:0045786, GO:0045787, GO:0051726
Fundamental Process	Translation	GO:0006412	GO:0032062, GO:0017148, GO:0140244, GO:0140245, GO:0140243, GO:0099578, GO:0099577, GO:0032055, GO:0043557, GO:0043555, GO:0045727, GO:1902010, GO:0006417, GO:0032061, GO:0032056, GO:0099547, GO:0036490, GO:0036493, GO:0043556, GO:0032939
Fundamental Process	Splicing	GO:0008380	GO:0033119, GO:0033120, GO:0042484
Neurogenesis	Nervous System Development	GO:0007399	GO:0051961, GO:0051960, GO:0051962
Neurogenesis	Synapse Organization	GO:0050808, GO:0045202	GO:0140241, GO:0099574, GO:0140243, GO:1905809, GO:0050807
Neurogenesis	Dendrite Development	GO:0016358, GO:0030425	GO:0050773, GO:2000171, GO:1900006
Neurogenesis	Axon Development	GO:0061564, GO:0030424	N/A
Neurogenesis	Myelination	GO:0042552, GO:0043209	GO:0031642, GO:0031641, GO:0031643, GO:0019911

Table S7. GO term choices for metric comparison in Figure 3. Does not show *is_a* or *part_of* descendants, which were also included.

Cell Type	Clusters (Overexpressed gene modules in parentheses)
iPSC	0 (m1), 5 (m1)
Neural ectoderm	1 (m90,m9); 3 (m6,m90,m118,m30); 6 (m14,m11,m90,m30); 15 (m13)
NPC	2 (m25); 4 (m11,m19,m51,m25,m50,m62); 7 (m11,m50,m91); 8 (m11); 9 (m10); 10 (m14,m51); 12 (m11,m91,m50,m25,m51,m62); 14 (m110,m91,m11,m50,m20,m51,m10,m62,m25,m12); 16 (m51,m8); 17 (m16,m90,m62,m14); 19 (m11); 20 (m50); 21 (m11,m91,m62,m25,m51,m50); 23 (m14)
Astrocyte	18 (m90,m3,m13); 22 (m9)
Cortical Neuron	11 (m3,m13,m27)
Astrocyte or neural ectoderm	13 (m90,m2,m6)

Table S8. The overexpressed modules for each GmGM cell cluster, using the marker genes from Appendix P's Table S6 to identify cell types. Each cluster had many statistically significantly overexpressed modules; here, we are only showing the most extremely overexpressed modules. Expression significance was calculated using a t-test, with p-values adjusted for multiple hypothesis testing by Benjamini-Hochberg.

the following, for data matrix $\mathbf{X}$ and pseudo-inverse $(\cdot)^\dagger$: $\left(\mathbf{X}\mathbf{X}^T\right)^\dagger$. Formally, this arises if one keeps all assumptions that GmGM makes and additionally enforces $\mathbf{\Psi}^{(genes)}$ to be $\mathbf{0}$. For cell network comparison, we thresholded this in the same manner as for GmGM (keeping the top 5 edges per vertex), and used Leiden clustering with a resolution chosen to yield the same amount of clusters as we had for GmGM (resolution=2.15).

Using the same marker list as before, we found that 21 of the 23 clusters were not significantly associated with *any* marker gene, representing 97% of all cells. This abysmal performance validates the multi-axis approach. It should be noted that there are more advanced single-axis estimators, such as the graphical lasso [Friedman et al., 2008], which is similar to this estimator but fit with an L1 penalty, which would likely perform better than this baseline estimator. The equivalent multi-axis work to the graphical lasso would be TeraLasso [Greenewald et al., 2019]. As we have seen in Figure 2, TeraLasso is not scalable to large datasets; for example, it takes a minute to fit 500-node networks, whereas our proposed GmGM variant takes less than a second.

S Neuronal scRNA-seq dataset full results

In this section, we give a breakdown of our gene modules and cell clusters. The GmGM cell clusters (and their links to the GmGM gene modules) are given in Table S8, the hdWGCNA gene modules are given in Table S9, and the GmGM gene modules are given in Table S10; there are no hdWGCNA cell clusters as hdWGCNA does not create a cell network. More detailed information for the modules, including GO IDs and adjusted p values for all the top GO terms, are available on the GitHub repository for this paper.

For the gene modules, we defined 'interesting' modules which satisfied one of the following criteria: high Moran's I (>0.3), high lncRNA percentage (>0.3), being related to transcription factors, being significantly expressed in a cell cluster, or being significantly expressed only in a specific passage.

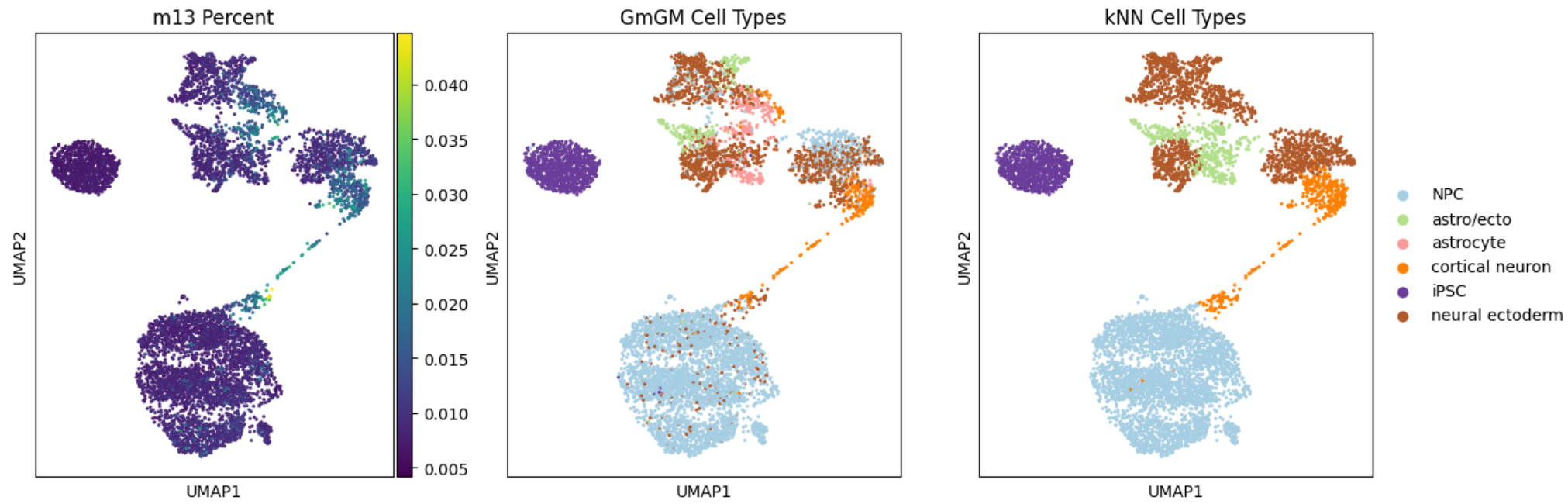

Fig. S7. (left) The average gene expression of the genes in GmGM module m13; it tends to express itself only in and around cells that are identified as cortical neurons. (middle) The cell type classification produced by GmGM using the marker genes from Appendix P. NPC stands for 'neural progenitor cell', and 'astro/ecto' denotes cells which could not be distinguished between astrocyte and neural ectoderm with the markers given. The non-NPC cells found by GmGM in the large NPC cluster (blue, bottom) correspond to the 'early differentiators' discussed in the text. (right) The cell type classification produced by kNN using the same marker genes as GmGM. (all) UMAP plots have been used for visualization; the overexpression of m13 in the suspected cortical neuron cells has also been validated statistically (Table S8).

Module	Top 3 genes by centrality	Select GO terms	Size	Annotation	Moran's I
Unassigned	N/A	N/A	14405	A catch-all module.	0.56
Grey	TSPAN6, CIBAR1, C3orf62	DNA-binding transcription factor activity, regulation of biological process	6399	Transcription factor module, only expressed in non-iPSCs.	0.99
Turquoise	LINC00678, LNCPRESS2, ESRG	gene expression, biological regulation, RNA binding	6146	Only expressed in iPSCs.	1.04
Blue	SOX6, ZBTB20, ENSG00000258631	nervous system development, neuron projection	903	Only expressed in non-iPSCs.	1.01
Brown	DMD, MIR100HG, ENSG00000286757	anatomical structure development, cell differentiation, cilium organization	559	Expressed only in a subset of p5s.	0.96
Yellow	HMGCS1, SCD, FADS1	cell differentiation, system development	409	Only expressed in p2/p3s.	0.94
Green	ECT2, SMC4, KIF20B	cell cycle, chromosome segregation, cell division	262	Cell cycle module.	0.83
Red	CFTR, SORCS3, PDGFD	regulation of biological process, nervous system development, cell differentiation	241	Only expressed in a subset of p5s.	0.91

Table S9. Every hdWGCNA module, in order of size. Moran's I is a statistic that expresses the tendency of 'nearby' cells to have a similar average expression of a given module. In this case, 'nearby' is defined by the Euclidean distance of gene expression vectors of each cell.

Every module of hdWGCNA was 'interesting'; in contrast, only 39 GmGM modules were. Interesting modules are likely indicative of some true biological process; for example, GmGM modules m5, m7, and m117 are all interesting, and based on the GO terms represent the cell cycle, the ribosome, and mitochondrial genes, respectively.

Module	Top 3 genes by centrality	Select GO terms	Size	Annotation	Moran's I
m0	DYNAP, DENRP3, ENSG00000250855	N/A	9047	A catch-all module.	0.15
**m1**	**FOXH1**, LAD1, FLT1	N/A	1756	Only expresses in iPSCs.	1.07
**m2**	CROCC2, ADGB, LMNTD1	cilium movement, cilium organization, axoneme assembly	600	Only expressed in a subset of p5s.	0.82
**m3**	SLC17A6, STMN2, **LHX9**	nervous system development, synaptic signalling, neuron projection morphogenesis	433	Only expresses in potential cortical neurons.	0.83
**m4**	ENSG00000288903, CARMN, CPED1	extracellular matrix organization, cell surface receptor signaling pathway	319	Only expressed in a subset of p5s.	0.71
**m5**	SGO2, KIF14, CCNB1	many cell-cycle related GO terms	315	Only expressed in a subset of p2/p3/p5s.	0.82
**m6**	PDZRN4, SGPP2, CT75	N/A	313	Only expresses in a subset of p4s, high amount of lncRNA, includes and is highly correlated with expression of GRM8.	0.94
**m7**	RPS6, RPL7A, RPS8	Ribosome-related GO terms	303	Ribosomal gene module.	0.77
**m8**	ANKRD1, SERPINE1, COL8A1	angiogenesis, vasculature development, DNA-binding transcription factor activity	300	Next three most central genes are all unnamed lncRNA; mostly expressed in p2s/p3s. One of three transcription-factor-related clusters.	0.58
**m9**	SPARCL1, SORCS3, CA12	branching morphogenesis of an epithelial tube, branching involved in ureteric bud morphogenesis, mesonephric tubule morphogenesis	276	Only expressed in a subset of p5 cells. Contains and correlates strongly with SORCS3.	0.89
**m10**	CDKN1A, INPP5D, ENSG00000251095	cell death/apoptosis GO terms	262	Not expressed in p5s.	0.57
**m11**	MALAT1, MT-RNR1, MT-RNR2	neurogenesis	251	Not expressed in iPSCs.	0.90
**m12**	BRIP1, DTL, ATAD2	DNA replication, DNA metabolic process, various cell-cycle terms	249	Only expressed in a subset of cells.	0.66
**m13**	**EBF2**, **NEUROD4**, **EBF3**	nervous system development, neurogenesis, DNA-binding transcription factor activity	239	One of three transcription-factor-related clusters, only expresses in potential cortical neurons.	0.70
**m14**	MIR9-2HG, **MEF2C-AS2**, WNT7A	nervous system development, cell differentiation, neurogenesis	212	Only expresses in a subset of p2/p3/p5 cells. Contains and correlates with he expression of MIR9-2HG. High lncRNA percentage.	0.76
m15	SEC24B, USB8, ZNF106	localization, nucleic acid biosynthetic process	204	N/A	0.04

Table S10 (*multi-page*)**.** Every GmGM module, in order of size. Bold genes either are, are antisense to, or differential transcripts of transcription factors. Select GO terms being N/A does not necessarily indicate a lack of GO terms, merely a lack of ones we felt worth highlighting. Bold modules were considered 'interesting'.

Module	Top 3 genes by centrality	Select GO terms	Size	Annotation	Moran's I
**m16**	LINC02306, ENSG00000253693, LINC03000	nervous system development, neurogenesis	203	Only expressed in a subset of p5s.	0.74
**m17**	CCT2, CCDC25, WDR1	N/A	201	N/A	0.39
m18	CSNK2A1, DDR1, GDI1	N/A	199	N/A	0.08
**m19**	TXNL1, TMEM178A, FXYD6	N/A	198	Significantly overexpressed in cell cluster 4.	0.40
**m20**	PRANCR, C1QTNF7-AS1, ENSG00000250195	N/A	198	Not expressed in iPSCs. High lncRNA percentage.	0.42
m21	MED27, MBTD1, **MGA**	N/A	186	N/A	0.06
m22	CLCN3, PHF20L1, **ZNF532**	N/A	184	N/A	0.11
**m23**	GDF7, **PAX3**, SV2C	anatomical structure morphogenesis, glutamatergic synapse	181	Only expressed in a subset of non-iPSC cells.	0.64
**m24**	CDC20B, CCNO, **FOXN4**	centriole assembly, centriole replication, de novo centriole assembly involved in multi-ciliated epithelial cell differentiation	180	Only expressed in a subset of p5 cells.	0.53
**m25**	NOS2, AGBL1, **BARHL1**	N/A	178	Mostly expressed in p2/p3 cells, some in p5s.	0.85
m26	LENG8, 7SK, ATN1	N/A	177	N/A	0.18
**m27**	**TBR1**, ISLR2, **LHX1-DT**	neuron projection morphogenesis, telencephalon development, synapse organization, dendrite, neurogenesis	177	Only expresses in potential cortical neurons.	0.76
m28	MTOR, PIN4, INO80D	nucleic acid metabolic process, nucleobase-containing compound metabolic process	175	N/A	0.23
m29	**UBP1**, INPP4A, PRKCSH	N/A	174	N/A	0.12
**m30**	RSPO2, **LMX1A**, DYNLRB2-AS1	regulation of cell differentiation, regulation of signal transduction	174	Expressed in all non-iPSC cells, more expression in a subset of p5 cells.	0.86
m31	CAPN2, EXOC6, BDNF-AS	gene expression	172	N/A	0.07
m32	BMP2K, FARP2, TAF2	gene expression, RNA biosynthetic process	171	N/A	0.08
m33	**E2F5**, CCDC136, PREPL	gene xpression	171	N/A	0.08
m34	CPSF7, FGGY, SLC20A2	transcription by RNA polymerase II, regulation of transcription by RNA polymerase II	170	N/A	0.07
m35	ARHGAP35, MAPKAP1, ORC2	N/A	169	N/A	0.05
m36	ZNF84, KDM5C, EXOC3	positive regulation of post-translational protein modification	169	N/A	0.05
m37	RPAP3, SAFB, PEX14	N/A	168	N/A	0.10
m38	RAD50, SFSWAP, TBCA	MLL3/4 complex	167	N/A	0.06
m39	DDX21, ENSG00000258131, LARP4	N/A	167	N/A	0.06
m40	RHEB, LINC00632, PRDX3	N/A	166	N/A	0.07
m41	MTREX, IP6K1, MED4	gene expression, localization, translation	164	N/A	0.16

Table S10 (*multi-page*)**.** Every GmGM module, in order of size. Bold genes either are, are antisense to, or differential transcripts of transcription factors. Select GO terms being N/A does not necessarily indicate a lack of GO terms, merely a lack of ones we felt worth highlighting. Bold modules were considered 'interesting'.

Module	Top 3 genes by centrality	Select GO terms	Size	Annotation	Moran's I
**m42**	CLEC2A, ENSG00000289578, NECTIN3-AS1	N/A	164	Expressed only in a subset of p2/p3 cells, high lncRNA percentage.	0.48
m43	PSMB7, KATNIP, SOS2	inositol phosphate metabolic process, "phosphotransferase activity, phosphate group as acceptor"	163	N/A	0.13
m44	MAPK8IP3, MED1, ANAPC1	Glycosylphosphatidylinositol (GPI)-anchor biosynthesis	163	N/A	0.09
m45	DZIP1, USP7, **AHDC1**	N/A	163	N/A	0.10
m46	GBF1, CERK, **MRTFA**	mitochondrion	161	N/A	0.08
m47	TARBP1, GCFC2, ESYT2	N/A	158	N/A	0.05
m48	SRP14, CEBPZ, CTTNBP2NL	RNA biosynthetic process, RNA metabolic process	158	N/A	0.14
m49	SLC38A6, MIER1, ICE2	proteolysis	157	N/A	0.11
**m50**	HMGCS1, MSMO1, HMGCR	sterol biosynthetic process, lipid biosynthetic process	157	Primarily expressed in p2/p3s, some expression in iPSCs and a subset of p5s.	0.61
**m51**	ENSG00000258312, MAMDC2, CCN1	hippo signaling, cellular response to stimulus	156	Only expressed in p2/p3s.	0.76
m52	SPG11, SSR1, SNX4	N/A	155	N/A	0.05
m53	UFL1-AS1, RGS12, MYH9	N/A	155	N/A	0.07
m54	TBC1D16, PRR14L, CMSS1	N/A	154	N/A	0.08
m55	WASL, DDAH2, PCGF3-AS1	gene expression	N/A	152	0.04
m56	XIST, LINC01515, HERC2P3	N/A	151	N/A	0.16
m57	RPS6KC1, PPFIA1, MCPH1	N/A	150	N/A	0.12
m58	CUL4B, QARS1, **PRDM4**	methylation	148	N/A	0.04
m59	LDAH, FUBP1, KLHDC10	N/A	147	N/A	0.05
m60	IFT81, GANAB, TNFAIP3	immune system response, immune system process	147	N/A	0.18
m61	ZNF782, ENSG00000263551, SLCO6A1	N/A	146	N/A	0.05
**m62**	CTNNA2, SMOC1, **SIM2**	neuron projection morphogenesis	145	Expressed in all non-iPSC cells.	0.80
**m63**	ATG5, ERCC5, PCBP1-AS1	RNA processing, RNA metabolic process, gene expression	144	Expressed in all cells, decreasing through passages.	0.34
m64	COPA, DHX57, MAPK1	N/A	144	N/A	0.06
m65	NDFIP1, SMURF1, TMEM245	gene expression, RNA metabolic processing, lncRNA processing	143	N/A	0.09
m66	RRAS2, FAM228B, IREB2	N/A	142	N/A	0.07
m67	CDK12, IARS1, ATP6V1D	tRNA metabolic process, RNA processing	141	N/A	0.10
m68	SENP5, AMOTL1, ATE1	N/A	140	N/A	0.16
m69	BMERB1, VPS4A, RIMKLB	N/A	140	N/A	0.12
m70	RICTOR, ZCCHC7, IGSF1	N/A	139	N/A	0.16
**m71**	ENSG00000197585, ENSG00000237461, KYAT3	N/A	139	High lncRNA percentage.	0.26
m72	SLC25A46, LUC7L2, COX16	N/A	139	N/A	0.08
m73	GARRE1, HSPA8, AKAP8L	N/A	138	N/A	0.17
m74	PPP2R2A, PGM3, MFSD8	N/A	138	N/A	0.05
m75	ZBED5, MRE11, TZNRD1	mitochondrial membrane organization	137	N/A	0.09
m76	RBM5, LAMP2, SCFD2	N/A	136	N/A	0.03
m77	NUMA1, NCK1, CFAP418-AS1	succinate dehydrogenase (quinone) activity	132	N/A	0.04
m78	UBE2D2, **ATF6**, SECISBP2L	N/A	132	N/A	0.09

Table S10 (*multi-page*)**.** Every GmGM module, in order of size. Bold genes either are, are antisense to, or differential transcripts of transcription factors. Select GO terms being N/A does not necessarily indicate a lack of GO terms, merely a lack of ones we felt worth highlighting. Bold modules were considered 'interesting'.

Module	Top 3 genes by centrality	Select GO terms	Size	Annotation	Moran's I
m79	NEK9, HYCC1, **ZNF577**	N/A	132	N/A	0.09
m80	WDR70, CTBP2, **FOXO3**	serotonergic synapse, gultamatergic synapse	131	N/A	0.10
m81	RHBDD1, BDP1, CDC14A	transcription factor TFIIIB complex	129	N/A	0.07
m82	ABL1, RAPH1, PIGU	N/A	127	N/A	0.07
m83	ENSG00000285713, RNF214, AMBRA1	N/A	127	N/A	0.04
m84	HADHA, TMSB10, ACTB	N/A	126	N/A	0.33
m85	DNAJB14, HFM1, INTU	positive regulation of protein-containing complex disassembly	125	N/A	0.06
m86	ZSWIM5, UBL3, PPM1A	N/A	124	N/A	0.08
m87	ARMCX4, BSDC1, DCTN5	DNA-templated transcription, regulation of DNA-templated transcription	121	N/A	0.09
m88	GALNT13, TP53BP2, ANAPC10	N/A	121	N/A	0.06
m89	KDELR2, MEMO1, AIG1	N/A	120	N/A	0.06
**m90**	**NFIA**, ENSG00000243620, **NFIB**	nervous system development, neuron differentiation, neurogenesis, DNA-binding transcription factor activity	120	One of the three transcription factor modules, highly expressed in p5s.	0.93
**m91**	ADAMTS18, **GLIS3**, LGR5	cell adhesion, cell junction assembly, long-term synapse potentiation	119	Not expressed in iPSCs, **NR2F1** and related genes are very central to the module as well.	0.85
m92	ATG2B, ALG11, MICU3	N/A	116	N/A	0.05
m93	CDC42SE2, RAB2A, FAM222B	N/A	115	N/A	0.16
m94	AHCTF1, CHIC1, DIS3L2	N/A	115	N/A	0.11
m95	AGO4, PLEKHA8, PHF6	N/A	115	N/A	0.18
m96	NUMB, DLG3, UBE2B	regulation of primary metabolic process, regulation of macromolecule metabolic process	113	N/A	0.07
m97	ARL13B, IFRD1, DPH6	N/A	108	N/A	0.07
m98	EML4, ELMO2, ANKRD6	N/A	107	N/A	0.10
m99	PRKCI, PTTG1IP, CCDC57	"hydrolase activity, acting on esther bonds"	104	N/A	0.04
**m100**	**LMX1B**, C8orf34, FZD10	N/A	103	Only expressed in a subset of p5 cells.	0.68
m101	CALM3, PRKRA, ENSG00000285920	N/A	102	N/A	0.09
m102	DDX18, ENSG00000291130, **ZBTB25**	rRNA metabolic process, rRNA processing	101	N/A	0.22
m103	TMEM168, NUP50, CCDC93	N/A	100	N/A	0.11
**m104**	**ETV5**, LEMD1, TPRKB	negative regulation of lens fiber cell differentiation, regulation of lens fiber cell differentiation, negative regulation of ERK1 and ERK2 cascade	96	Expressed in all cells, but expression much higher in subset of p5 cells.	0.25
**m105**	H1-5, H1-2, H2AC8	nucleosome assembly, chromatin remodeling, protein-DNA complex organization	95	Present in all passages, but only in a subset of each.	0.55
**m106**	SUPT20H, ATP6V1C1, ENSG00000289349	N/A	95	High lncRNA percent.	0.06

Table S10 (*multi-page*)**.** Every GmGM module, in order of size. Bold genes either are, are antisense to, or differential transcripts of transcription factors. Select GO terms being N/A does not necessarily indicate a lack of GO terms, merely a lack of ones we felt worth highlighting. Bold modules were considered 'interesting'.

Module	Top 3 genes by centrality	Select GO terms	Size	Annotation	Moran's I
m107	STX16, CTNND1, OBSCN	N/A	95	N/A	0.05
m108	SEC22A, TIAL1, SNHG29	nucleic acid biosynthetic process	94	N/A	0.08
m109	NUP107, DNAJB6, NBR1	N/A	91	N/A	0.08
**m110**	LINC02646, DIRC3, PGK1	pyruvate metabolic process, monosaccharide metabolic process, HIF1-signaling pathway, Glycolosis / Gluconeogenesis	91	Not expressed in p5s.	0.38
m111	TMTC1, ATXN1, KRAS	N/A	85	N/A	0.08
m112	KLHL7, CASTOR3P, AP2A2	N/A	85	N/A	0.09
**m113**	CDH18, NAV3, SIPA1L3	N/A	84	Expressed in all cells, but more so in certain subsets.	0.47
m114	MCPH1-AS1, BCL9, CRYZL1	N/A	78	N/A	0.09
m115	SMARCB1, CLEC2D, PRKD1	N/A	74	N/A	0.08
m116	SCP2, GSPT1, ZMAT1	N/A	63	N/A	0.04
**m117**	MT-CYB, MT-ND2, MT-ATP6	oxidative phosphorylation, ATP synthesis coupled electron transport, NADH dehydrogenase activity	48	Consists of mitochondrial genes. Expressed in all cells, less in p5s.	0.35
**m118**	ENSG00000227681, SAMD5, OSBPL1A	N/A	37	Expressed only in a subset of non-iPSC cells (all passages).	0.69

Table S10 (*multi-page*)**.** Every GmGM module, in order of size. Bold genes either are, are antisense to, or differential transcripts of transcription factors (such as **LHX-DT**). Select GO terms being N/A does not necessarily indicate a lack of GO terms, merely a lack of ones we felt worth highlighting. Bold modules were considered 'interesting'.

=2
